# Association between firearms and mortality in Brazil, 1990 to 2017: a global burden of disease Brazil study

**DOI:** 10.1186/s12963-020-00222-3

**Published:** 2020-09-30

**Authors:** Deborah Carvalho Malta, Adauto Martins Soares Filho, Isabella Vitral Pinto, Maria Cecília de Souza Minayo, Cheila Marina Lima, Ísis Eloah Machado, Renato Azeredo Teixeira, Otaliba Libânio Morais Neto, Roberto Marini Ladeira, Edgar Merchan-Hamann, Maria de Fatima Marinho de Souza, Cíntia Honório Vasconcelos, Carlos Cezar Flores Vidotti, Ewerton Cousin, Scott Glenn, Catherine Bisignano, Adrienne Chew, Antonio Luiz Ribeiro, Mohsen Naghavi

**Affiliations:** 1 Nursing School, Universidade Federal de Minas Gerais, Belo Horizonte, Brazil; 2 Ministry of Health of Brazil, Brasilia, Brazil; 3 Graduate Program in Public Health, School of Medicine, Universidade Federal de Minas Gerais, Belo Horizonte, Brazil; 4 Sergio Arouca National School of Public Health, Fundação Oswaldo Cruz, Rio de Janeiro, Brazil; 5 School of Medicine, Universidade Federal de Ouro Preto, Ouro Preto, Brazil; 6 Institute of Tropical Pathology and Public Health, Universidade Federal de Goiás, Goiânia, Brazil; 7 Fundação Hospitalar do Estado de Minas Gerais, Belo Horizonte, Brazil; 8 Universidade de Brasília, Brasilia, Brazil; 9 Postgraduate Program in Epidemiology, Universidade Federal do Rio Grande do Sul, Porto Alegre, Brazil; 10 Institute for Health Metrics and Evaluation, University of Washington, Seattle, WA USA; 11 Telehealth Center, Hospital das Clínicas and Internal Medicine Department, School of Medicine, Universidade Federal de Minas Gerais, Belo Horizonte, Brazil

**Keywords:** Brazil, Firearms, Homicide, Mortality, Epidemiology

## Abstract

**Background:**

Brazil leads the world in number of firearm deaths and ranks sixth by country in rate of firearm deaths per 100,000 people. This study aims to analyze trends in and burden of mortality by firearms, according to age and sex, for Brazil, and the association between these deaths and indicators of possession and carrying of weapons using data from the global burden of diseases, injuries, and risk factors study (GBD) 2017.

**Methods:**

We used GBD 2017 estimates of mortality due to physical violence and self-harm from firearms for Brazil to analyze the association between deaths by firearms and explanatory variables.

**Results:**

Deaths from firearms increased in Brazil from 25,819 in 1990 to 48,493 in 2017. Firearm mortality rates were higher among men and in the 20–24 age group; the rate was 20 times higher than for women in the same age group. Homicide rates increased during the study period, while mortality rates for suicides and accidental deaths decreased. The group of Brazilian federation units with the highest firearm collection rate (median = 7.5) showed reductions in the rate of total violent deaths by firearms. In contrast, the group with the lowest firearm collection rate (median = 2.0) showed an increase in firearm deaths from 2000 to 2017. An increase in the rate of voluntary return of firearms was associated with a reduction in mortality rates of unintentional firearm deaths (*r* = −0.364, *p* < 0.001). An increase in socio-demographic index (SDI) was associated with a reduction in all firearm death rates (*r* = −0.266, *p* = 0.008). An increase in the composite index of firearms seized or collected was associated with a reduction in rates of deaths by firearm in the subgroup of females, children, and the elderly (*r* = −0.269, *p* = 0.005).

**Conclusions:**

There was a change in the trend of firearms deaths after the beginning of the collection of weapons in 2004. Federation units that collected more guns have reduced rates of violent firearm deaths.

## Background

The global burden of diseases, injuries, and risk factors study (GBD) estimated the occurrence of 251,000 firearms deaths globally in 2016, resulting from homicides, suicides, and unintentional causes [1]. Half of these deaths occurred in six countries: Brazil (43200), the USA (37200), Mexico (15400), Colombia (13300), Venezuela (12800), and Guatemala (5090). Thus, Brazil accounted for about one-sixth of all firearms deaths [1]. There are large global differences in rates, ranging from 0.2 deaths per 100,000 inhabitants in Singapore in 2016 to 39.4 deaths per 100,000 in El Salvador during the same period [1], which may be partly explained by differences in socioeconomic standing, inequalities, cultural perspectives, and availability of weapons [2]. High rates of gunshot homicide are concentrated in the Americas, in a belt that runs from Mexico to Brazil (including the Caribbean). The region has been associated with drug cartels as well as the manufacture and sale of firearms and their illegal trade [3–5]. The illegal trade in firearms is one of the main determinants of the complex problem of interpersonal violence [2, 6, 7].

Global studies indicate that high mortality rates from violence by firearm are intrinsically related to greater availability of weapons [1, 8]. Other factors associated with firearm mortality are the caliber of the firearm, which determines the lethality of the weapon [9]; the existence of groups linked to the drug trade, arms, theft of goods, and control of territories; and the consumption of alcohol and drugs [10–13]. A study shows that in Australia, after the law that banned possession of weapons and the campaign for the voluntary return of arms, in 1997, the number of deaths from firearms was reduced and collective massacre by firearms no longer occurred [14]. In 2000, the South Africa Firearms Control Act was approved, prohibiting the possession of weapons except under specific conditions. Law enforcement studies have pointed out that there was a 13.5% reduction in rates of violent deaths per year after the arms control law; more than 4500 deaths were prevented in five South African cities between 2001 and 2005 [15].

In 2003, the Brazilian Congress approved the Disarmament Act, which expanded regulations on the registration, possession, carrying, and sale of firearms and ammunition, and was also defined by the voluntary return of firearms and ammunition [16]. In 2005, there was a referendum to ban the marketing of firearms and ammunition. After strong lobbying by pro-firearms groups, including congressmen, ruralists, and the armaments industry, the marketing of firearms and ammunition remained legal, with restrictions [16]. In January 2019, the new Brazilian government published a decree amending the rules and expanding the possibility of possessing weapons in homes and commercial establishments throughout the country [17].

Access to firearms is a necessary precondition for firearm injury to occur [1], making it important to monitor the new measures in Brazil. Thus, this study aims to analyze the trend in mortality by firearms by age and sex, for states, and nationally for Brazil, and the association between these deaths and indicators of possession and carrying of weapons.

## Methods

### Overview

This study used estimates from the global burden of diseases, injuries, and risk factors study (GBD) 2017, using methodology devised by the Institute for Health Metrics and Evaluation (IHME), University of Washington, USA [15]. GBD uses globally comparable data, adjusted for underreporting, age, and numerous other variables, and includes estimates for Brazil and its component federation units. Each edition of GBD incorporates new data sources and methodological advances in relation to previous editions [18].

The GBD 2017 database contains data from vital records for specific causes of death by firearm and also includes verbal autopsy data, censuses, surveys, hospital data, police records, and forensic medical services for some injuries [18]. Specific data sources used in the estimates can be seen using the GBD data tool: http://ghdx.healthdata.org/gbd-2017/data-input-sources. The main source of mortality data in Brazil was the death registration database of the Ministry of Health’s Mortality Information System. http://datasus.saude.gov.br/informacoes-de-saude/tabnet/estatisticas-vitais.

GBD methodology included standardizing methods to adjust the data for completeness and quality of the underlying cause of death, including ill-defined causes, also known as “garbage codes” [1]. To define the country’s performance, a star rating system (maximum 5) was created by GBD in 2016, which awarded stars proportionally to the percentage of well-certified deaths throughout the time series. In this calculation, the following parameters were considered: completeness of the record of the cause of death; fraction of deaths not classified as major garbage codes; and fraction of deaths classified as attributed to detailed causes for GBD. Brazil was certified with four stars and some federation units with five stars [18], 12 federative units located in north and northeast of Brazil were classified with three stars and the Federal District was classified with five stars.

For estimates of external causes, which include homicides, suicides, and unintentional accidents, the redistribution of the “garbage codes” for the defined cause groups was performed. Details of clustering of causes using the International Classification of Diseases (ICD) ninth and tenth revisions were previously described [19]. GBD 2017 used ICD-9 and ICD-10: in ICD-9, E000–E999, and codes 800–999; in ICD-10, subgroups V01 to Y98 of chapter XX and subgroups S00 to T98 of chapter XIX. The development and documentation of GBD 2017 follow the guidelines for accurate and transparent health estimates reporting (GATHER) [20].

In this study, we present, based on data from GBD 2017, firearm mortality rates by sex, age group, and percentage change for Brazil. For the three outcomes related to firearms deaths, we compared differences between 1990 and 2017, by absolute numbers and standardized rates per 100,000 inhabitants, for Brazil and its 27 states, considering the Federal District as a state to analyze the subnational level in this study.

### Statistics

For the regression analysis, the dependent variables were subgroups of firearm death rates per 100,000 inhabitants for unintentional firearms deaths, suicides, firearm suicides, homicides, firearm homicides, and firearm-related deaths. Firearm mortality rates were also calculated separately for the population subgroup females of all ages and males under 15 and over 60 years, because that group is considered vulnerable.

The explanatory variables were constituted by the following indexes: Socio-demographic Index (SDI)—a composite index that combines information on per capita income, average years of schooling in the population older than 15 years, and fertility rate under age 25. This index has a value that varies between 0 (representing the lowest level of development, lowest per capita income, the lowest level of schooling, and highest fertility rate); and 1 (representing the highest per capita income, highest level of schooling, and lowest fertility rate). SDI was also used as a categorical variable: low SDI, low-middle SDI (reference), high-middle SDI, and high SDI. These were defined according to the quartile of SDI in 27 Brazilian states in the period 2000–2016.

The following indexes, obtained from police records, are used to measure the number of firearms available in the population: (b1) rate of voluntary return of firearms to the authorities, (b2) rate of police records for illegal carrying of firearms, (b3) rate of police records for narcotics trafficking, (b4) rate of police-registered incidents involving civilian possession and use of narcotics, (b5) composite index of firearms seized or collected that combines rates b1 and b2 [= 1/(b1 + b2)].

The indexes (b1 to b5) collectively constitute a proxy measure for the number of firearms in a population, as there are no complete data on the total number of weapons circulating in Brazil, since many weapons are not registered because they are smuggled into the country or are possessed by traffickers. Studies show that official records on legal possession of legal weapons are insufficient and most often incomplete [1, 8].

The sources referring to (b1), rate of voluntary return of firearms to the authorities (presented as number of firearms collected per 100,000 civilians), were obtained through the Law of Access to Information as part of the National Campaign for Disarmament and analyzed for the period 2005–2017. Proxy indexes for carrying weapons are detailed in the supplementary material.

The Pearson correlation coefficient was applied to analyze the association between firearm deaths and the explanatory variables for the period from 2005 to 2016, controlled by the variable year. Sample size was not always 27 states because data were not available for all federation units and all years of the period. The Spearman correlation coefficient was calculated for the SDI categorical variables.

Multiple linear regression analysis was performed for each type of firearm death using each explanatory variable described above. The goal was to create a standard model of explanation for differing rates of firearm deaths using the stepwise method. Explanatory variables that showed potential for multicollinearity, and confirmed in the regression, were excluded from the models tested. Due to data availability, two data periods were tested, 2005–2016 and 2013–2016. The assumptions of adequacy of the models were observed in the graphical analysis of the residuals (normal probability and homoscedasticity), including the Kolmogorov-Smirnov test of the residual standardization, and independence of errors (Durbin Watson’s chart and statistics).

Finally, comparative analyses were performed for two groups of federation units, according to rates of voluntary return of firearms. First, we calculated the third quartile for all values of federated units from 2000 to 2017, which was 6 arms collected per 100,000 inhabitants. Then the 27 states were divided into two groups according to the median of voluntary return rate from 2005 to 2016: the federation units group with the highest voluntary return of arms (median > 6 per 100,000 inhabitants), and the federation units group with the lowest voluntary return of arms (median < 6 per 100,000 inhabitants). The median rates of mortality due to firearm homicides as well as all violent deaths by firearms (the collective term for physical violence by firearm, self-harm by firearm including suicide, and unintentional injury by firearm) for each of these groups of federation units (major and minor arms deliveries) were analyzed for the period 2000–2017.

The GBD Brazil study was approved by the Research Ethics Committee of the Federal University of Minas Gerais (CAAE number Project—62803316.7.0000.5149). The GBD study used only non-nominal aggregate secondary data in accordance with Brazilian Resolution No. 510 of 7 April 2016, about research ethical standards [21].

### Role of the funding source

The funders of the study had no role in study design, data collection, data analysis, data interpretation, or the writing of the report. All authors had full access to the data in the study and had final responsibility for the decision to submit for publication.

## Results

**Fig. 1:**
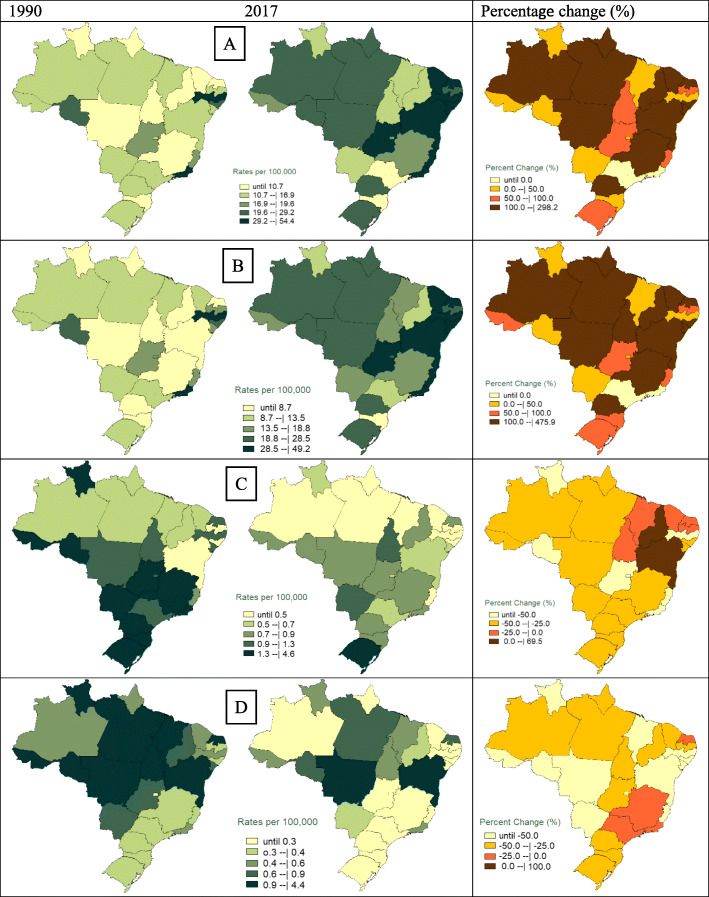
Mortality rates related to firearms (per 100,000), annualized rate of change between 1990 and 2017 federation units and Brazil, 1990–2017, (**a**) total by firearm, (**b**) homicide by firearm, (**c**) suicide by firearm, (**d**) unintentional death by firearm

Figure 1 and supplemental table 1 show that firearms deaths increased from 25,819 (95% uncertainty interval [UI] 24,750.3–28,736.1) to 48,493 (42,750.8–50,042.4) from 1990 to 2017, with a stable age-standardized mortality rate of 17.5 (16.5–19.8) to 21.5 (18.6–22.6) per 100,000 and an increase in firearm homicides from 14.8 (14.4–20.4) per 100,000 in 1990 to 20.4 (17.7–21.1) per 100,000 in 2017. Suicides involving firearms decreased from a mortality rate of 1.7 (1.4–2.3) to 0.7 (0.5–1.1) per 100,000, and unintentional firearms deaths decreased from 0.9 (0.8–1.0) to 0.4 (0.4–0.5 ) per 100,000.

**Fig. 2:**
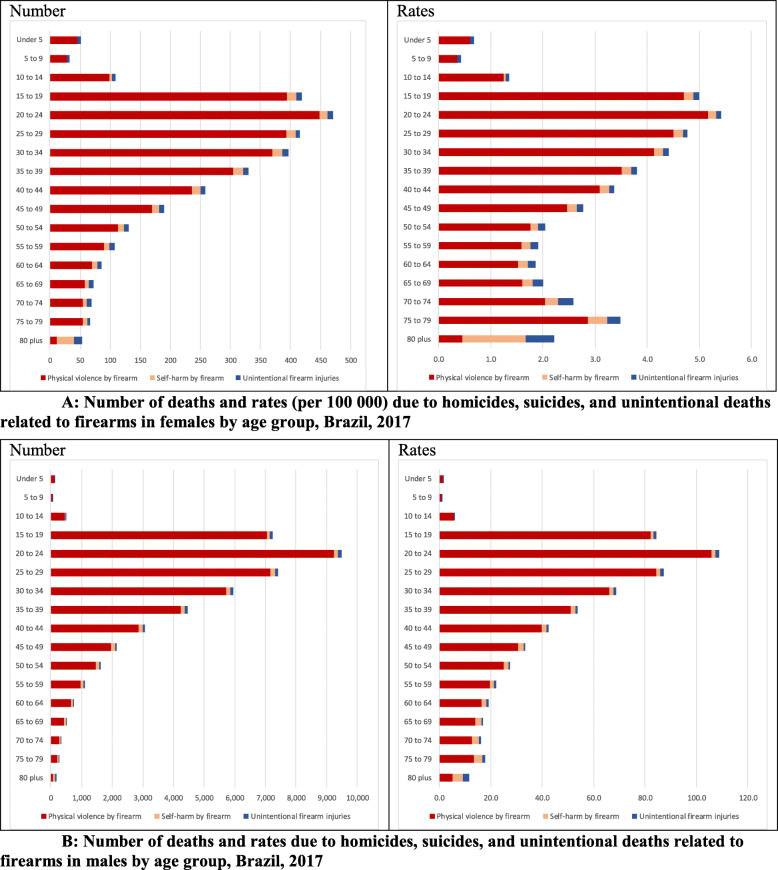
Number of deaths and rates (per 100 000) due to homicides, suicides, and unintentional deaths related to firearms by age group **a**) in females and **b**) in males, Brazil, 2017

**Fig. 3:**
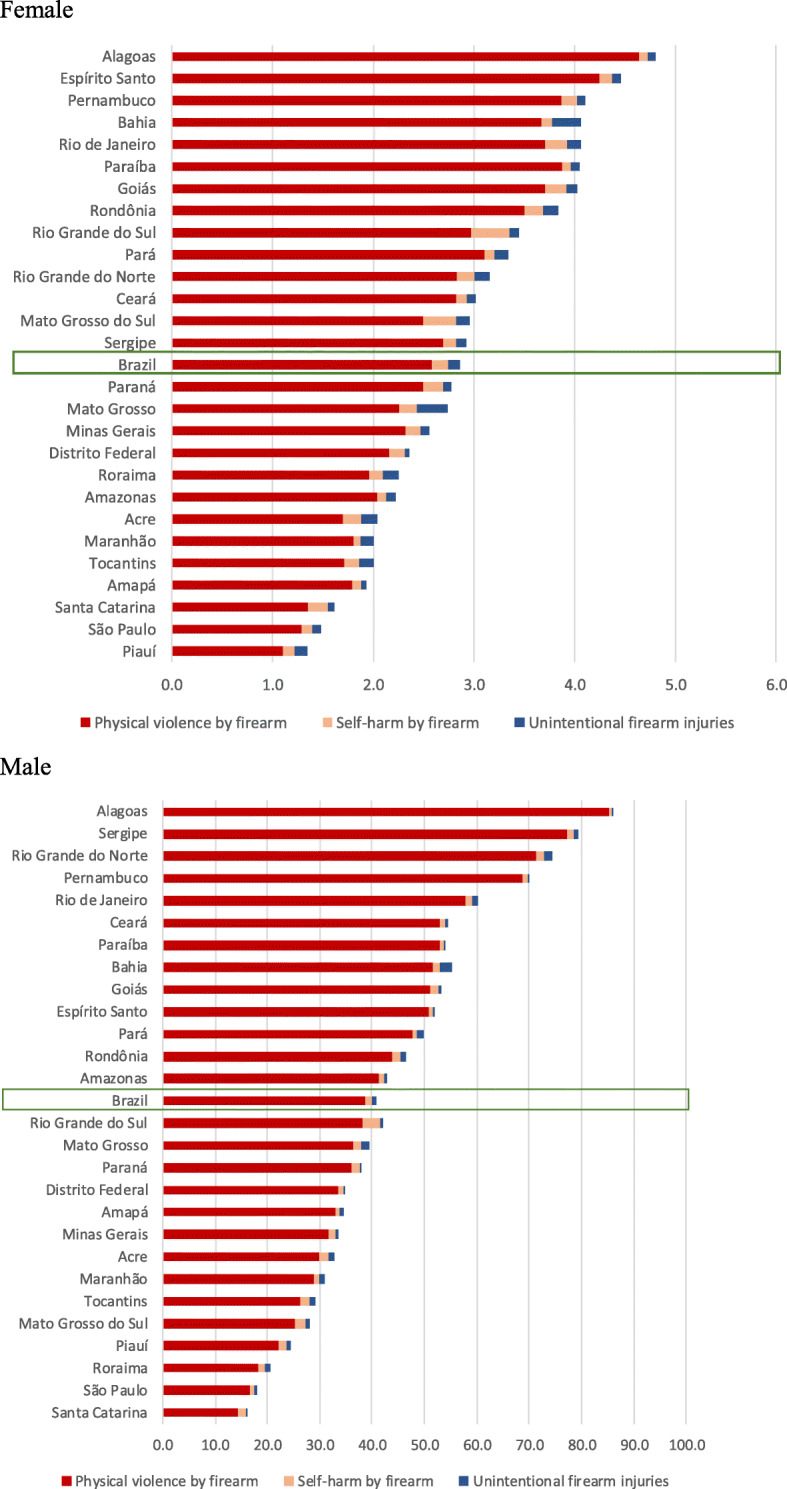
Mortality rates (per 100 000) related to firearms according to federation units and sex, Brazil, 2017

The firearm-related death rates per 100,000 inhabitants were higher in males than females in 2017. Homicides were concentrated in young males aged 20–24 (rate = 105.8 per 100,000), 25–29 years (rate = 84.3), and 15–19 years (rate = 82.2). The rate of firearm deaths among males aged 20–24 years old was 20.35 times higher than that among females in the same age group, for whom it was 5.2 deaths per 100,000. Rates declined with increasing age. Suicide and unintentional firearm deaths were also higher among males; rates of firearm-related suicide increased with age, reaching higher rates among the elderly (Fig. 2a and b). Rates of firearm death were higher among males than females in 2017, not just at the national level but in all Brazilian federation units (Fig. 3).

**Fig. 4:**
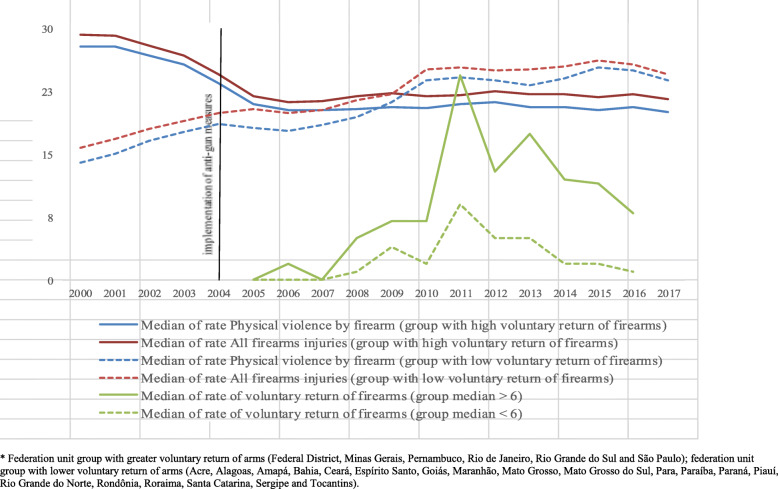
Evolution of median rates of total violent deaths related to firearms and firearm homicides, and median rates of voluntary return of firearms, according to two groups of federation units: the group with the highest rate of voluntary return of firearms and the group with the lowest rate of voluntary return of firearms, Brazil, 2000–2017

Figure 4 shows the evolution of the medians of the rate of firearm homicides and rate of total violent deaths by firearms in each group of federation units according to the rate of voluntary return of firearms in the period 2000–2017. The group of Brazilian federation units with the highest firearm collection rate (median = 7.5, interquartile range 3.5–12.5) showed reductions in the rate of total violent deaths by firearms (annualized percent change—APC = −1.5) and firearms homicides (APC = −1.6) throughout the period. Brazil saw its most significant reductions in these causes of death between 2003 and 2005 (APC of −9.0 and −9.1), coinciding with the beginning of the implementation of disarmament measures. In the following years, mortality rates from these causes continued to decline, although with lower intensity (APC = −0.1; −0.4). The group with the lowest firearm collection rate (median = 2.0 per 100,000, inhabitants, interquartile range 0.5–4.5) experienced a trend of increasing death rates (APC of 3.3 and 4.1 for total violent deaths by firearms and firearm homicides, respectively) from 2000 to 2017, although there was a deceleration in firearm homicide rates (APC = 1.4) in the post-implementation period of the disarmament statute (2003–2005). In 2017, federation units with the highest voluntary return of firearms to the authorities exhibited lower rates of violent deaths due to physical violence (20.8) and all firearms injuries (22.5) than federation units with low voluntary return (26.7 and 28.3 due to physical violence and all firearms injuries, respectively).

**Fig. 5:**
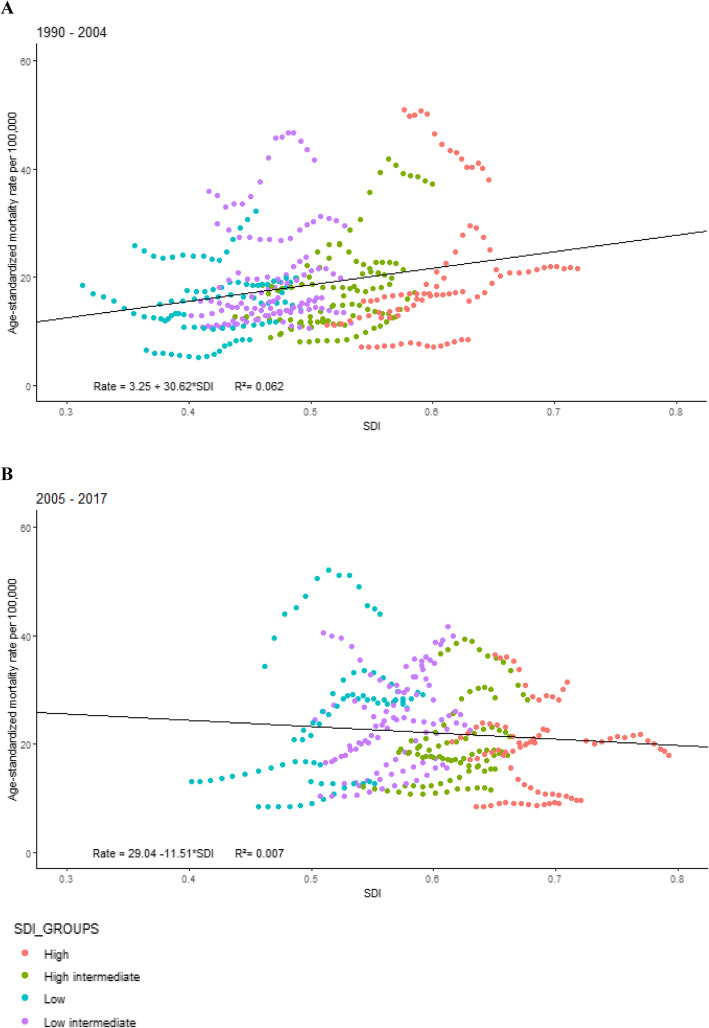
Relationship between age-standardized mortality rate due to firearms and SDI in the Brazilian federation units, (**a**) 1990–2004 and (**b**) 2005–2017

Figure 5 compares the relationship between age-standardized mortality rates due to firearms and SDI level by the Brazilian state. In the period from 1990 to 2004, mortality rates increased in most federation units; federation units with low and low-middle SDI showed higher and increasing rates, while federation units with high and high-middle SDI presented lower rates and also increasing rates. In the period from 2005 to 2017, after the beginning of the voluntary return of firearms to the authorities, the pattern reversed, with great declines in the federation units with better socio-demographic status (high and high-middle SDI); the decline was more rapid in Rio de Janeiro, São Paulo, and the Federal District, while increases occurred in lower SDI federation units, but not as quickly.

Table 1 shows that an increase in the composite index of firearms seized or collected (b5) was associated with a reduction in firearm deaths in the subgroup of vulnerable females, children, and the elderly (*r* = −0.269, *p* = 0.005). An increase in the rate of voluntary return of arms (b1) was associated with a reduction in unintentional firearm deaths (*r* = −0.364, *p* < 0.001). An increase in SDI was associated with a reduction in the total firearm death rate (*r* = −0.266, *p* = 0.008), firearm homicide rate (*r* = −0.266, *p* = 0.008), and unintentional firearm death rate (*r* = −0.316, *p* = 0.001). SDI subgroup level (low, low-middle, high-middle, and high) was correlated with the rate of firearm deaths for almost all specific causes of firearm deaths; however, low SDI federation units were associated with lower total rates of firearms deaths (*r* = −0.366, *p* < 0.001) and rates of firearm homicides (*r* = −0.391, *p* < 0.001), but with the highest unintentional rates of firearm deaths (*r* = 0.471, *p* < 0.001). The final models produced in the multiple linear regression analysis for each outcome that presented statistical significance were (i) total violent firearm deaths in the vulnerable subgroup (females, children, elderly) from 2005 to 2016 (*p* = 0.004, *R*^2^ = 0.089); (ii) unintentional firearm deaths (*p* < 0.001, *R*^2^ = 0.144); (iii) total violent firearm deaths (*p* = 0.008, *R*^2^ = 0.060); and (iv) firearm homicides (*p* = 0.008, *R*^2^ = 0.060) in the period 2013–2016 (Table 2). Details of rates of total violent deaths related to firearms and firearms homicides and rates of voluntary return of firearms in Brazil are available in supplemental table 2 and 3. The graphical and statistical analysis of the residues assures the adequacy of the model, taking into account the regression assumptions (supplemental figure 1).

**Table 1:** Correlation between rates of violence by firearms and proxy indexes for carrying weapons, controlled by year, of the Brazilian federation units, 2005-2016

	Unintentional firearm deaths	Suicide	Firearm suicide	Homicide	Firearm homicide	Firearm-related death	Firearm death for vulnerable subgroup (female, elderly, child)
	Correlation (*p* value)						
SDI	−0.316 (0.001)	0.249 (0.013)	0.329 (0.001)	−0.327 (0.001)	−0.267 (0.008)	−0.266 (0.008)	−0.018 (0.741)*
Voluntary return of firearms	−0.364 (< 0.001)	0.115 (0.257)*	0.269 (0.007)	−0.199 (0.049)	−0.064 (0.526)*	0.064 (0.526)*	0.122 (0.229)*
Illegal carrying of firearms	−0.146 (0.149)*	0.109 (0.284)*	0.230 (0.022)	−0.005 (0.957)*	0.019 (0.852)*	0.025 (0.807)*	0.220 (0.028)
Possession and use of narcotics	−0.247 (0.014)	0.174 (0.085)*	0..370 (< 0.001)	−0.319 (0.001)	−0.254 (0.011)	−0.250 (0.013)	0.015 (0.882)*
Narcotics trafficking	−0.262 (0.009)	0.182 (0.071)*	0.282 (0.005)	−0.308 (0.002)	−0.230 (0.022)	−0.229 (0.023)	0.082 (0.420)*
Composite index^a^	0.204 (0.035)	0.023 (0.817)*	−0.126 (0.195)*	−0.016 (0.872)*	−0.058 (0.555)*	−0.058 (0.525)*	−0.269 (0.005)
SDI low	0.471 (< 0.001)	−0.309 (< 0.001)	0.001 (0.964)*	−0.317 (< 0.001)	−0.391 (< 0.001)	−0.366 (< 0.001)	0.070 (0.206)*
SDI middle high	−0.214 (< 0.001)	0.231 (< 0.001)	0.039 (0.214)*	0.222 (< 0.001)	0.233 (< 0.001)	0.221 (< 0.001)	−0.036 (0.524)*
SDI high	−0.379 (< 0.001)	0.089 (0.004)	−0.053 (0.090)*	0.084 (0.007)	0.185 (< 0.001)	0.166 (< 0.001)	−0.083 (0.135)*

*Not significant (*p* > 0.05)

^a^[= 1/(voluntary return of firearms+illegal carrying of firearms)]

**Table 2:** Variables associated with rates of violent firearm death in the federation units of Brazil (coefficients of the multiple linear regression final model)

Model	Unstandardized coefficients		Standardized coefficients	*t*	Sig.	95% confidence interval for *B*		Collinearity statistics		*R* square (adjusted)	Durbin-Watson	ANOVA	Kolmogorov-Smirnov *Z* (*p* value)
	*B*	Std error	Beta			Lower bound	Upper bound	Tolerance	VIF				
Dependent variable: all firearm injuries in the vulnerable subgroup (women, children, elderly) (2005-2016)													
(Constant)	4.967	0.253		19.654	< 0.001	4.465	5.469						
Composite index^a^	−16.877	5.607	−0.297	−3.010	< 0.001	−28.005	−5.750	0.946	1.057				
SDI high	−0.73802	0.326	−0.223	−2.266	0.030	−1.385	−0.092	0.946	1.057	0.107 (0.089)	1.689	0.004	0.855 (0.458)
Dependent variable: unintentional firearm injuries (2013-2016)													
(Constant)	1.048	0.240		4.374	< 0.001	0.572	1.523						
Voluntary return of firearms	−0.011	0.004	−0.268	−2.719	0.008	−0.019	−0.003	0.891	1.122				
SDI	−0.895	0.393	−0.224	−2.276	0.025	−1.675	−0.115	0.891	1.122	0.161 (0.144)	2.181	0.000	1.262 (0.083)
Dependent variable: all firearms injuries (2013-2016)													
(Constant)	52.057	9.805		5.309	< 0.001	32.599	71.515						
SDI	−42.267	15.631	−0.264	−2.704	0.008	−73.286	−11.248	1.000	1.000	0.069 (0.060)	2.238	0.008	0.812 (0.524)
Dependent variable: physical violence by firearm (2013-2016)													
(Constant)	51.290	9.931		5.164	< 0.001	31.581	70.998						
SDI	−42.928	15.832	−0.264	−2.711	0.008	−74.347	−11.510	1.000	1.000	0.070 (0.060)	2.236	0.008	0.932 (0.350)

^a^[= 1/(voluntary return of firearms+illegal carrying of firearms)]

## Discussion

In 2017, there were more than 45,000 deaths from firearms in Brazil, and more than half were among males between the ages of 15 and 29 years. From 1990 to 2017 there was an increase in firearm homicides and a reduction in suicides and accidental deaths by firearms. Homicides increased in most federation units, and the highest rates were observed in the federation units of Alagoas, Sergipe, and Rio Grande do Norte. The federation units with significant reductions in rates were Rio de Janeiro and São Paulo. In the period 1990 to 2004, firearms death rates increased in most federation units. In the period from 2005 to 2017, after the voluntary return of firearms to the authorities, the pattern reversed, with large declines in the federation units with the highest SDI; while firearms death rates in federation units with low SDI also declined, though not so rapidly.

Analyses showed that firearm death rates in the vulnerable group (women, children, the elderly) decreased with increases in voluntary return of firearms to the authorities and with increases in SDI.

Brazil stands out as one of the most violent countries in the world. It ranks first in absolute number of deaths by firearms and sixth in firearms mortality, behind several countries in Central America and the Caribbean, with death rates from firearms per 100,000 inhabitants such as those in El Salvador (43.1), Venezuela (42.1), Guatemala (29.6), Honduras (23.5), Jamaica (23.5), and Colombia (22.4). Previous research has suggested that such high firearms mortality rates may be associated with trafficking of drugs such as cocaine, illegal markets, and homicides [10, 22].

Controlling the possession of firearms is an important constraint on the occurrence of injury and death by firearms. In Brazil, high rates of possession and carrying of firearms and young males living in areas with poor socio-demographic conditions are strong facilitators of the occurrence of such deaths [23]. Prior research has found that restricting the availability and use of firearms can significantly reduce firearm deaths [14, 15]. However, monitoring and evaluating these interventions is still a challenge in Brazil due to a lack of information that would make it possible to establish baselines and monitor weapons nationally and in critical areas. In addition, the entry of illegal weapons is not measured. Estimating the total number of weapons held by the civilian population is a challenging task that has yielded inaccurate and unreliable results. It is a challenge to account for the number of weapons circulating in the country among civilians, since not all weapons are legally registered, and methods for estimating the number of illegal firearms in circulation, smuggled into the country, or in the possession of militia or traffickers are inadequate [2]. The lack of quality information makes it difficult to accurately estimate firearm availability and thus does little to subsidize public security policies and interventions.

Firearms collection may have reduced homicide rates. Federation units with higher rates of firearm collection decreased their rates (São Paulo, Rio de Janeiro, Distrito Federal, others), while federation units with lower rates of firearm collection experienced an increase in firearm homicide rates. Similarly, positive outcomes have been observed in countries that applied regulatory measures for the use of firearms, such as Australia [1, 2, 14], South Africa [15], and Colombia [24, 25]. Japan, which banned the possession of firearms and rifles in 1945, stands out as having one of the lowest firearm homicide rates in the world, at 0.03 per 100,000 inhabitants [1]. A 1997 study investigating the impact of these measures on indicators of violence found that state gun control laws in the USA had a much smaller effect on firearms than socioeconomic variables did [21]. This study did point out that systems of law vary widely between states, and if there were a more uniform arms control law in the USA, the impact could have been stronger [21].

In Brazil, authors have pointed to the importance of voluntary arms collection measures in reducing firearm deaths [2, 26, 27]. The reduction of firearm homicides in the last decade in São Paulo and of suicides in the state of Rio de Janeiro may be associated with firearms delivery programs created through the Disarmament Act [26, 27]. Cerqueira [2] has argued that fewer weapons lead to less crime, and therefore disarmament policies can reduce violent crime. Brazil’s experience of a reduction in firearm deaths and hospitalizations following firearm control and restriction legislation in 2003 supports this argument [26].

Control of the use of firearms by civilians may reduce certain rates of violence in Brazil. For every ten weapons taken from circulation, it has been estimated that more than two lives have been spared [24]. Concern over firearm violence and death in Brazil has led to changes beyond national legislation. In 2013 at the United Nations, Brazil and 60 other countries signed the Arms Trade Treaty to regulate the international arms trade in order to reduce the number of firearms diverted to illegal trafficking. This regulation, which Brazil has adhered to, is important because the country’s arms industry is the fourth largest exporter of light weapons in the world, according to the Small Arms Survey. It is ahead of countries such as Israel, Austria, and Russia. According to the Brazilian Army, which was responsible for controlling exports from 2005 to 2010, Brazil exported 4,482,874 firearms [8] and sold them to countries in Africa and Asia. The Brazilian gun industry moves approximately $100 million a year [8].

Our study also shows the impact of SDI on the reduction of homicides and accidental deaths, which can be explained by the multi-causal nature of violence and by the intimate association between poverty, social inequalities, family disintegration, and domestic violence, as well as by the absence and/or precariousness of social policies and public security and in the existing barriers to access to justice [12, 28, 29].

Suicides were more frequent among the elderly and in federation units of the south and southeast regions, particularly in the state of Rio Grande do Sul, whose population contains a higher percentage of elderly people. Suicides have been declining in the country, but other countries have also observed concentrations of suicide among their elderly populations [1].

Globally, deaths from firearms are higher among males than females [1], and higher among young adults than other age groups. This pattern occurs in Brazil and is associated with social and economic inequalities [1, 28]. The main victims of firearm violence are young, poor, less-educated, black, unemployed, or inhabitants of the outskirts of the cities, which are generally degraded areas with low cohesion and social support [28, 30]. Previous research has also found that young adult males have greater exposure to high-risk situations such as alcohol abuse, drug trafficking, and drug use [31], and are more likely to have criminal records [8, 32]. Prior studies point to the association between illicit drug use and homicide [22, 31]. Other factors associated with firearm violence are lack of investment in public security, police violence, and conflicts over land disputes and areas of agricultural frontiers [33].

Federation units in the north and northeast regions contributed most to the increase in homicide. From 2000 to 2015, upward trends in homicide rates in the country were at the expense of growth in small and medium-sized municipalities [34]. Situations such as clashes over agrarian issues and territorial conflict over assignment of indigenous lands and quilombolas have led to increases in violent deaths [35]. Organized crime and disputes between armed factions have increased violence in northeast federation units like Ceará and Rio Grande do Norte [36].

Many Brazilian cities bordering other countries, such as at the triple border of Brazil, Paraguay, and Argentina, have become critical territories for violence, as they serve as entry corridors for drugs and weapons [28, 30]. This may explain the increase in rates of firearm mortality in border federation units such as Paraná and Acre. In contrast, lower rates are observed in Southeastern federation units [28] like São Paulo and Rio de Janeiro, which can be explained by greater investments in public security as well as social policies, economic improvement, employment, and income in these regions [28, 37].

Although firearm mortality rates were much higher among men than women, Brazil also has notably high rates of femicides, murders of females because of their gender, ranking fifth in the world for rates of these occurrences. Female murder victims follow a similar profile to male victims as being young, black, single, less educated, and/or murdered due to their status as a woman [38].

It is therefore essential to monitor the United Nations Sustainable Development Goals (SDGs), which advocate a new model for global development that prioritizes peace [39]. Brazil is a signatory to the goal of “peaceful and inclusive societies for sustainable development, providing access to justice for all, and creating effective, accountable, and inclusive institutions at all levels” [39]. The SDGs are the first universal structure to make explicit the connection between violence, conflict, and development, stating that “development that cannot be achieved without peace and security is unsustainable.” It has among the indicators to be measured intentional killings and direct deaths from conflicts [39]. The SDGs thus demand an unprecedented global shift in attention to reducing violent deaths as a means of facilitating global development [39].

Contrary to the commitment of the SDGs [39], Brazil’s federal government relaxed regulations on the possession of weapons in the country in January 2019 [17]. Therefore, it is essential moving forward to monitor the increase in the possession of weapons by the civilian population and the possible increase in violent deaths in the country.

This study had several limitations. One limitation is that data on the number of circulating weapons are not available, necessitating the use of proxy measures; however, these indicators depend on the performance of the public security system in reducing the occurrence of violence. In order to circumvent this limitation, a group of indicators that together represent an approximation of the number of circulating weapons, in particular a composite index, the voluntary return of firearms, and illegal carrying of firearms, as described in the methods [27]. Since this is an observational study, inference on causal relations is always limited and subject to confounding by unmeasured factors. Strong cultural pressures to avoid legal implications of homicide and the stigma associated with suicide homicides and suicides may be misclassified as unintentional firearm injury deaths [40], specially when intent is not clear. This fact can also impact the garbage code redistribution process of undetermined intention deaths, specially in states where SIM present lower quality of causes of death.

## Conclusions

This study concludes that Brazilian federation units with more weapons collected reduced their mortality rates, confirming the importance of the disarmament statute approved in 2003. Public policies of disarmament of the civilian population should be reinforced since they can reduce the number of violent deaths in Brazil and other Latin American countries. Rigorous measures to combat smuggling and illegal commerce of arms, as well as reliable registration of the available firearms, are also useful and necessary measures. It is essential to monitor the Brazilian government’s policies aimed at facilitating access to firearms in the country and how they could impact rates of firearm mortality.

## Supplementary information

**Additional file 1: Figure S1.** Residual graphs for each outcome of violent firearms deaths in the federation units, Brazil, 2013–2016. Residual graphs of multiple linear regression models for deaths due to unintentional, physical violence and all firearms injuries for all ages and for total population and for vulnerable subgroup (females, children, elderly) in Brazil, 2013–2016.

**Additional file 2: Table S1.** Mortality related to firearms in absolute numbers, age-standardised mortality rate (per 100,000), annualised rate of change between 1990 and 2017, for Brazil and by state, for total by firearm, homicide by firearm, suicide by firearm, and unintentional death by firearm, 1990–2017.

**Additional file 3: Table S2.** Median rates of total violent deaths related to firearms and firearms homicides, and median rates of voluntary return of firearms, according to two groups of states, the group with the highest rate of voluntary return of firearms and the group with the lowest rate of voluntary return of firearms, Brazil, 2000–2017.

**Additional file 4: Table S3.** Rate per year of indicators, for Brazil and by state, 1990 to 2017. Legend: * [=1/(voluntary return of firearms+illegal carrying of firearms)].

## Data Availability

Cause of death data analyzed in this study are available in the GBD data tool: http://ghdx.healthdata.org/gbd-2017/data-input-sources. The main source of mortality data in Brazil was the death registration database of the Ministry of Health’s Mortality Information System: http://datasus.saude.gov.br/informacoes-de-saude/tabnet/estatisticas-vitais. Data related to the number of firearms available in the population are available through the DataCrime portal, the Getúlio Vargas Foundation, and the Brazilian Yearbook of Public Security, based on information from the State Secretariats of Public Security and/or Social Defense, the Brazilian Institute of Geography and Statistics, and the Brazilian Forum on Public Security.

## Abbreviations

APC: Annualized percent change
GBD: Global burden of diseases, injuries, and risk factors study
ICD: International classification of diseases
IHME: Institute for Health Metrics and Evaluation
SDGs: Sustainable development goals
SDI: Socio-demographic index
SIM: Mortality information system
UI: Uncertainty interval
