# Supplementary material for: Association between firearms and mortality in Brazil, 1990 to 2017: a global burden of disease Brazil study
Source: Popul Health Metr. 2020 Sep 30;18(Suppl 1):19. doi: 10.1186/s12963-020-00222-3 (PMC7525968; doi:10.1186/s12963-020-00222-3)

**Figure 1: Residual graphs for each outcome of violent firearms deaths in the federation units, Brazil, 2013–2016**

**Unintentional firearm injuries**


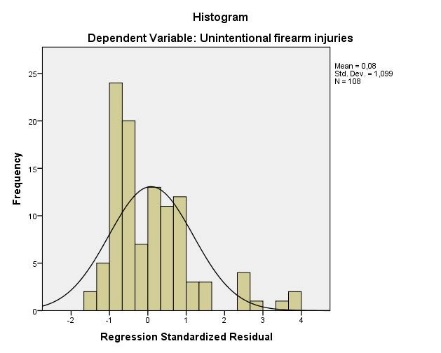

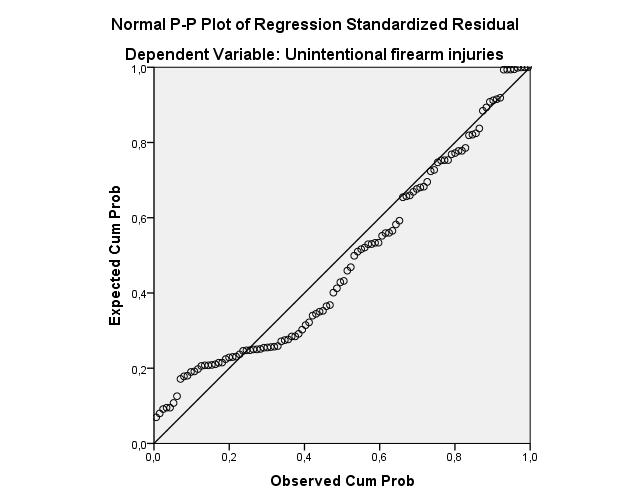

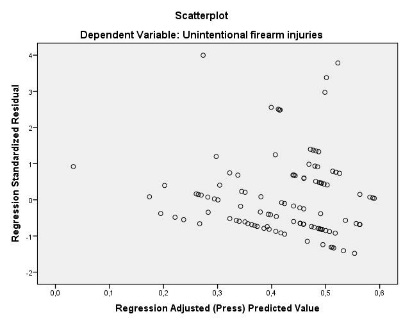


**All firearms injuries**


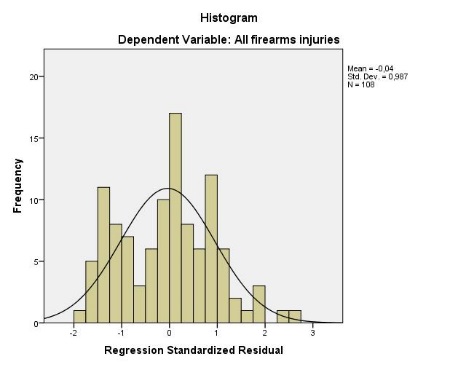

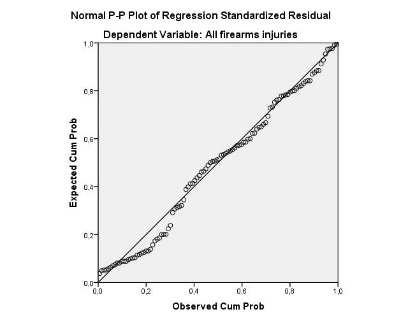

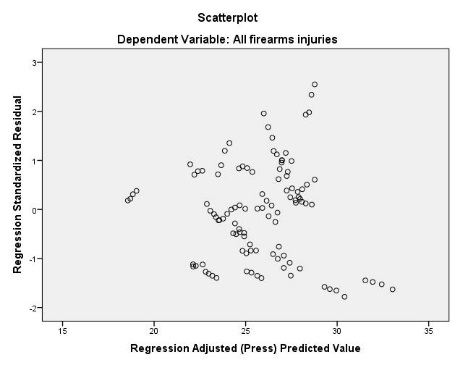


**Physical violence by firearm**


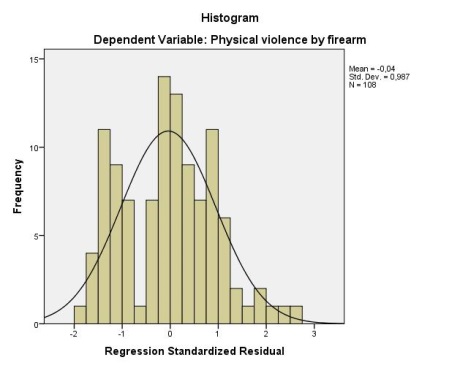

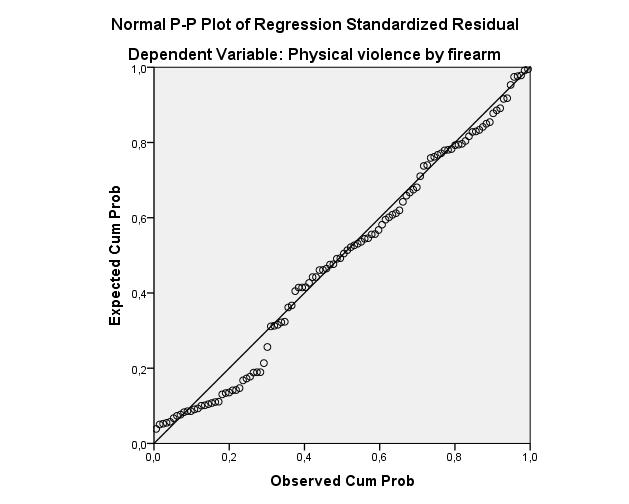

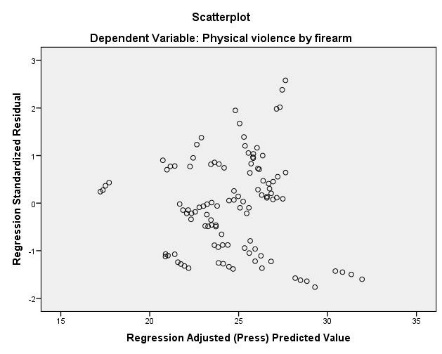


**All firearms injuries in the vulnerable subgroup (females, children, elderly)**


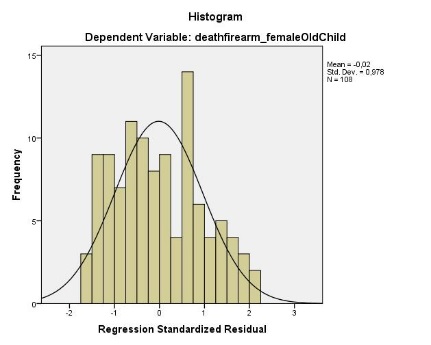

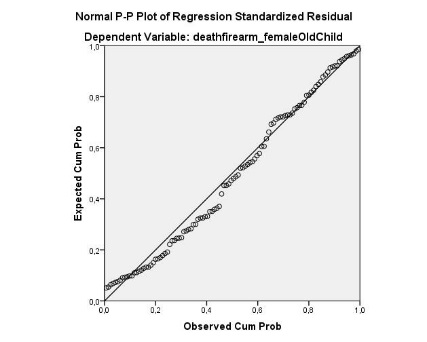

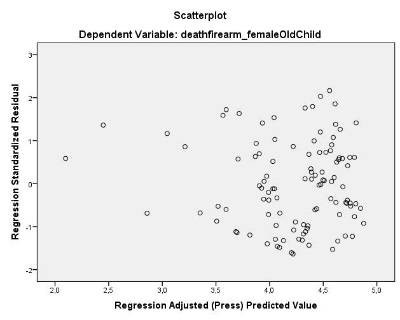

Supplement: Supplementary file 1 — Additional file 1: Figure S1. Residual graphs for each outcome of violent firearms deaths in the federation units, Brazil, 2013–2016. Residual graphs of multiple linear regression models for deaths due to unintentional, physical violence and all firearms injuries for all ages and for total population and for vulnerable subgroup (females, children, elderly) in Brazil, 2013–2016. [file 12963_2020_222_MOESM1_ESM.docx]
