# Supplementary material for: Association between firearms and mortality in Brazil, 1990 to 2017: a global burden of disease Brazil study
Source: Popul Health Metr. 2020 Sep 30;18(Suppl 1):19. doi: 10.1186/s12963-020-00222-3 (PMC7525968; doi:10.1186/s12963-020-00222-3)
Supplement: Supplementary file 2 — Additional file 2: Table S1. Mortality related to firearms in absolute numbers, age-standardised mortality rate (per 100,000), annualised rate of change between 1990 and 2017, for Brazil and by state, for total by firearm, homicide by firearm, suicide by firearm, and unintentional death by firearm, 1990–2017. [file 12963_2020_222_MOESM2_ESM.docx]

| **Supplemental table**  **1: Mortality related to firearms in absolute numbers, age-standardised mortality rate (per 100,000), annualised rate of change between 1990 and 2017, for Brazil and by state, for total by firearm, homicide by firearm, suicide by firearm, and unintentional death by firearm, 1990–2017** | | | | | | |
| --- | --- | --- | --- | --- | --- | --- |
| **Location** | **Cause** | **Number of deaths** | | **Age-standardised mortality rate per 100000** | | **Annualised percentage change (%)** |
|  |  | **1990** | **2017** | **1990** | **2017** | **1990–2017** |
| Brazil | Firearm death | 25819 | 48493 | 17·5 | 21·5 | 0·773 |
|  |  | (24750·3; 28736·1) | (42750·8; 50042·4) | (16·8; 19·3) | (19; 22·2) | (-0·044; 0·944) |
|  | Homicide by firearm | 22207 | 45927 | 14·8 | 20·4 | 1·189 |
|  |  | (21359; 25055) | (39887; 47521) | (14·3; 16·5) | (17·7; 21·1) | (0·248; 1·365) |
|  | Suicide by firearm | 2270 | 1628 | 1·7 | 0·7 | -3·27 |
|  |  | (1765; 2981) | (1263; 2435) | (1·4; 2·3) | (0·5; 1·1) | (-3·742; -2·722) |
|  | Unintentional firearm death | 1342 | 938 | 0·9 | 0·4 | -2·816 |
|  |  | (1222; 1442) | (846; 998) | (0·8; 1·0) | (0·4; 0·5) | (-3·179; -2·513) |
| Acre | Firearm death | 58 | 166 | 15 | 17·3 | 0·525 |
|  |  | (52·6; 66) | (147·1; 182·3) | (13·7; 17·3) | (15·4; 19) | (-0·266; 0·934) |
|  | Homicide by firearm | 45 | 152 | 11·4 | 15·7 | 1·199 |
|  |  | (40; 53) | (133; 168) | (10·3; 13·4) | (13·8; 17·4) | (0·246; 1·700) |
|  | Suicide by firearm | 7 | 8 | 2·3 | 1 | -3·054 |
|  |  | (5; 9) | (6; 13) | (1·7; 2·9) | (0·7; 1·5) | (-3·909; -2·178) |
|  | Unintentional firearm death | 5 | 5 | 1·4 | 0·6 | -2·984 |
|  |  | (5; 7) | (4; 6) | (1·2; 1·7) | (0·5; 0·7) | (-3·723; -2·354) |
| Alagoas | Firearm death | 594 | 1644 | 25·9 | 43·9 | 1·952 |
|  |  | (477·8; 644·8) | (1253·1; 1745·1) | (20·3; 28·2) | (33·4; 46·6) | (1·601; 2·305) |
|  | Homicide by firearm | 573 | 1628 | 25 | 43·4 | 2·071 |
|  |  | (462; 623) | (1243; 1727) | (19·5; 27·2) | (33·1; 46·1) | (1·713; 2·453) |
|  | Suicide by firearm | 11 | 10 | 0·5 | 0·3 | -2·25 |
|  |  | (6; 16) | (6; 13) | (0·3; 0·8) | (0·2; 0·4) | (-3·331; -1·342) |
|  | Unintentional firearm death | 11 | 6 | 0·4 | 0·2 | -3·168 |
|  |  | (5; 13) | (3; 7) | (0·2; 0·5) | (0·1; 0·2) | (-3·945; -2·280) |
| Amapá | Firearm death | 23 | 167 | 8·7 | 18·1 | 2·686 |
|  |  | (20·4; 32·9) | (146·6; 183·4) | (7·8; 12·4) | (16;19·7) | (1·059; 3·237) |
|  | Homicide by firearm | 20 | 161 | 7·3 | 17·3 | 3·227 |
|  |  | (17; 29) | (139; 177) | (6·5; 10·7) | (15·0; 19·0) | (1·353; 3·849) |
|  | Suicide by firearm | 2 | 4 | 0·9 | 0·5 | -2·466 |
|  |  | (1; 3) | (3; 6) | (0·6; 1·2) | (0·3; 0·7) | (-3·304; -1·612) |
|  | Unintentional firearm death | 1 | 3 | 0·5 | 0·3 | -1·702 |
|  |  | (1; 2) | (2; 3) | (0·5; 0·6) | (0·3; 0·4) | (-2·366; -1·093) |
| Amazonas | Firearm death | 246 | 997 | 12·6 | 22·5 | 2·135 |
|  |  | (223·7; 269·6) | (833·6; 1075·4) | (11·5; 13·8) | (18·8; 24·2) | (1·283; 2·538) |
|  | Homicide by firearm | 219 | 964 | 11·1 | 21·6 | 2·508 |
|  |  | (198; 241) | (793; 1044) | (10·1; 12·1) | (17·8; 23·4) | (1·582; 2·956) |
|  | Suicide by firearm | 15 | 21 | 0·9 | 0·5 | -2·141 |
|  |  | (11; 20) | (15; 36) | (0·7; 1·3) | (0·4; 0·9) | (-3·305; -0·967) |
|  | Unintentional firearm death | 12 | 12 | 0·6 | 0·3 | -2·519 |
|  |  | (11; 15) | (10; 16) | (0·5; 0·7) | (0·2; 0·4) | (-3·220; -1·914) |
| Bahia | Firearm death | 1459 | 4801 | 12·8 | 29·1 | 3·035 |
|  |  | (1320·7; 1648) | (4012·2; 5088·6) | (11·6; 14·5) | (24·3; 30·7) | (1·942; 3·477) |
|  | Homicide by firearm | 870 | 4478 | 7·8 | 27·1 | 4·727 |
|  |  | (765; 999) | (3709; 4758) | (6·9; 8·9) | (22·4; 28·7) | (3·475; 5·246) |
|  | Suicide by firearm | 51 | 112 | 0·5 | 0·7 | 0·85 |
|  |  | (33; 64) | (80; 170) | (0·3; 0·7) | (0·5; 1·0) | (-0·254; 2·068) |
|  | Unintentional firearm death | 538 | 211 | 4·5 | 1·3 | -4·491 |
|  |  | (482; 621) | (188; 230) | (4·0; 5·3) | (1·2; 1·4) | (-5·124; -4·004) |
| Ceará | Firearm death | 653 | 2907 | 10·9 | 28·2 | 3·527 |
|  |  | (539·3; 747·2) | (2404·1; 3097·7) | (9·2; 12·4) | (23·3; 30) | (2·843; 4·122) |
|  | Homicide by firearm | 574 | 2824 | 9·5 | 27·4 | 3·976 |
|  |  | (472; 664) | (2322; 3007) | (8·0; 11·0) | (22·5; 29·2) | (3·255; 4·628) |
|  | Suicide by firearm | 45 | 53 | 0·8 | 0·5 | -1·706 |
|  |  | (30; 64) | (39; 85) | (0·5; 1·1) | (0·4; 0·8) | (-3·031; -0·415) |
|  | Unintentional firearm death | 33 | 29 | 0·5 | 0·3 | -2·028 |
|  |  | (22; 40) | (19; 34) | (0·3; 0·6) | (0·2; 0·3) | (-2·763; -1·128) |
| Distrito Federal | Firearm death | 265 | 579 | 15·6 | 18 | 0·525 |
|  |  | (243·8; 286·9) | (491·8; 618·1) | (14·5; 16·8) | (15·3; 19·2) | (0·086; 0·858) |
|  | Homicide by firearm | 241 | 559 | 13·9 | 17·3 | 0·821 |
|  |  | (222; 261) | (476; 597) | (12·8; 15·0) | (14·7; 18·5) | (0·322; 1·179) |
|  | Suicide by firearm | 20 | 17 | 1·5 | 0·6 | -3·5 |
|  |  | (14; 25) | (12; 24) | (1·0; 1·9) | (0·4; 0·8) | (-4·179; -2·817) |
|  | Unintentional firearm death | 4 | 3 | 0·2 | 0·1 | -3·342 |
|  |  | (3; 4) | (2; 3) | (0·2; 0·3) | (0·1; 0·1) | (-3·865; -2·825) |
| Espírito Santo | Firearm death | 518 | 1148 | 20 | 28·1 | 1·250 |
|  |  | (483·5; 589·7) | (1001·6; 1218·3) | (18·8; 22·6) | (24·5; 29·8) | (0·293; 1·573) |
|  | Homicide by firearm | 482 | 1122 | 18·5 | 27·5 | 1·471 |
|  |  | (449; 555) | (976; 1193) | (17·3; 21·1) | (24·0; 29·2) | (0·424; 1·829) |
|  | Suicide by firearm | 23 | 17 | 1 | 0·4 | -3·481 |
|  |  | (16; 29) | (11; 22) | (0·7; 1·3) | (0·3; 0·5) | (-4·107; -2·848) |
|  | Unintentional firearm death | 12 | 9 | 0·5 | 0·2 | -2·993 |
|  |  | (9; 13) | (7; 10) | (0·4; 0·5) | (0·2; 0·2) | (-3·555; -2·485) |
| Goiás | Firearm death | 745 | 2103 | 18·5 | 28·6 | 1·616 |
|  |  | (689·3; 812·6) | (1763·8; 2240·9) | (17·2; 20·1) | (24; 30·4) | (0·844; 1·954) |
|  | Homicide by firearm | 618 | 2016 | 15 | 27·4 | 2·253 |
|  |  | (570; 683) | (1677; 2148) | (13·9; 16·4) | (22·8; 29·1) | (1·423; 2·614) |
|  | Suicide by firearm | 100 | 63 | 2·8 | 0·9 | -4·216 |
|  |  | (80; 136) | (49; 101) | (2·3; 3·8) | (0·7; 1·4) | (-4·835; -3·541) |
|  | Unintentional firearm death | 27 | 23 | 0·7 | 0·3 | -2·735 |
|  |  | (21; 30) | (18; 26) | (0·5; 0·8) | (0·3; 0·4) | (-3·260; -2·252) |
| Maranhão | Firearm death | 762 | 1279 | 18·4 | 16·1 | -0·499 |
|  |  | (640·9; 881·6) | (1084·2; 1391·8) | (15·6; 21·3) | (13·6; 17·5) | (-1·13; 0·124) |
|  | Homicide by firearm | 669 | 1198 | 16·2 | 15 | -0·285 |
|  |  | (561; 773) | (1004; 1309) | (13·7; 18·8) | (12·5; 16·5) | (-0·911; 0·368) |
|  | Suicide by firearm | 28 | 34 | 0·8 | 0·5 | -1·901 |
|  |  | (15; 44) | (23; 48) | (0·4; 1·2) | (0·3; 0·6) | (-3·838; -0·399) |
|  | Unintentional firearm death | 65 | 47 | 1·4 | 0·6 | -3·069 |
|  |  | (47; 80) | (38; 53) | (1·0; 1·7) | (0·5; 0·7) | (-3·820; -1·871) |
| Mato Grosso | Firearm death | 171 | 819 | 9·4 | 21·4 | 3·031 |
|  |  | (142·7; 397·9) | (753·3; 885·1) | (7·9; 20·8) | (19·7; 23·1) | (-0·228; 3·719) |
|  | Homicide by firearm | 75 | 756 | 4·3 | 19·6 | 5·827 |
|  |  | (57; 274) | (687; 818) | (3·3; 14·2) | (17·8; 21·2) | (0·802; 6·865) |
|  | Suicide by firearm | 23 | 29 | 1·5 | 0·8 | -2·268 |
|  |  | (16; 29) | (21; 44) | (1·0; 1·8) | (0·6; 1·2) | (-3·270; -1·343) |
|  | Unintentional firearm death | 73 | 34 | 3·7 | 1 | -4·867 |
|  |  | (61; 114) | (27; 59) | (3·1; 6·0) | (0·8; 1·7) | (-5·476; -4·372) |
| Mato Grosso do Sul | Firearm death | 294 | 461 | 17 | 15·4 | -0·363 |
|  |  | (251·7; 324·4) | (421·9; 499·3) | (14·9; 18·7) | (14·1; 16·7) | (-0·745; 0·058) |
|  | Homicide by firearm | 236 | 413 | 13·3 | 13·8 | 0·14 |
|  |  | (201; 261) | (375; 448) | (11·4; 14·7) | (12·6; 15·0) | (-0·286; 0·601) |
|  | Suicide by firearm | 42 | 36 | 2·8 | 1·2 | -3·014 |
|  |  | (33; 54) | (28; 53) | (2·2; 3·6) | (0·9; 1·8) | (-3·756; -2·287) |
|  | Unintentional firearm death | 16 | 12 | 1 | 0·4 | -3·063 |
|  |  | (13; 18) | (10; 14) | (0·8; 1·1) | (0·4; 0·5) | (-3·603; -2·426) |
| Minas Gerais | Firearm death | 1224 | 4031 | 8 | 18 | 2·989 |
|  |  | (1110·8; 1503·4) | (3697·8; 4295·4) | (7·3; 9·8) | (16·4; 19·1) | (1·998; 3·393) |
|  | Homicide by firearm | 925 | 3782 | 5·9 | 16·9 | 3·966 |
|  |  | (830; 1184) | (3424; 4035) | (5·3; 7·6) | (15·2; 18·1) | (2·628; 4·418) |
|  | Suicide by firearm | 233 | 179 | 1·6 | 0·7 | -2·915 |
|  |  | (187; 337) | (135; 292) | (1·3; 2·4) | (0·6; 1·2) | (-3·668; -2·235) |
|  | Unintentional firearm death | 67 | 70 | 0·5 | 0·3 | -1·409 |
|  |  | (48; 75) | (57; 78) | (0·3; 0·5) | (0·3; 0·3) | (-1·913; -0·702) |
| Pará | Firearm death | 687 | 2539 | 15·7 | 26·6 | 1·964 |
|  |  | (592·7; 756·8) | (1973·7; 2713·9) | (13·6; 17·2) | (20·5; 28·4) | (1·217; 2·394) |
|  | Homicide by firearm | 604 | 2437 | 13·7 | 25·5 | 2·319 |
|  |  | (519; 670) | (1881; 2606) | (11·8; 15·1) | (19·5; 27·2) | (1·517; 2·774) |
|  | Suicide by firearm | 34 | 40 | 0·9 | 0·5 | -2·332 |
|  |  | (23; 42) | (27; 58) | (0·6; 1·1) | (0·3; 0·7) | (-3·148; -1·546) |
|  | Unintentional firearm death | 50 | 62 | 1·1 | 0·7 | -1·635 |
|  |  | (42; 58) | (54; 76) | (0·9; 1·2) | (0·6; 0·8) | (-2·253; -1·003) |
| Paraíba | Firearm death | 530 | 1242 | 17·5 | 28·4 | 1·784 |
|  |  | (440·5; 585·8) | (1019·1; 1389) | (14·5; 19·4) | (23·3; 31·8) | (1·293; 2·361) |
|  | Homicide by firearm | 505 | 1215 | 16·7 | 27·8 | 1·914 |
|  |  | (417; 559) | (996; 1361) | (13·7; 18·5) | (22·7; 31·1) | (1·413; 2·558) |
|  | Suicide by firearm | 14 | 17 | 0·5 | 0·4 | -1 |
|  |  | (8; 19) | (12; 26) | (0·3; 0·7) | (0·3; 0·6) | (-2·525; 0·297) |
|  | Unintentional firearm death | 12 | 10 | 0·4 | 0·2 | -1·981 |
|  |  | (9; 15) | (7; 12) | (0·3; 0·5) | (0·2; 0·3) | (-2·909; -0·608) |
| Paraná | Firearm death | 924 | 2412 | 11·1 | 20·2 | 2·213 |
|  |  | (855·4; 1044·7) | (2194·2; 2570·6) | (10·3; 12·5) | (18·3; 21·6) | (1·498; 2·564) |
|  | Homicide by firearm | 738 | 2280 | 8·6 | 19·1 | 2·997 |
|  |  | (679; 839) | (2077; 2432) | (8·0; 9·8) | (17·3; 20·4) | (2·147; 3·369) |
|  | Suicide by firearm | 152 | 104 | 2·1 | 0·8 | -3·336 |
|  |  | (123; 214) | (80; 164) | (1·7; 3·0) | (0·6; 1·3) | (-3·899; -2·779) |
|  | Unintentional firearm death | 33 | 28 | 0·4 | 0·2 | -2·117 |
|  |  | (29; 37) | (24; 33) | (0·4; 0·5) | (0·2; 0·3) | (-2·623; -1·583) |
| Pernambuco | Firearm death | 2473 | 3748 | 36 | 36·1 | 0·011 |
|  |  | (2006·9; 2612·7) | (3078·3; 3977·8) | (28·6; 38) | (29·6; 38·3) | (-0·243; 0·302) |
|  | Homicide by firearm | 2363 | 3677 | 34·3 | 35·4 | 0·118 |
|  |  | (1907; 2503) | (3001; 3909) | (27·0; 36·3) | (28·9; 37·6) | (-0·143; 0·411) |
|  | Suicide by firearm | 75 | 51 | 1·2 | 0·5 | -3·344 |
|  |  | (53; 100) | (35; 72) | (0·9; 1·6) | (0·3; 0·7) | (-3·902; -2·830) |
|  | Unintentional firearm death | 34 | 21 | 0·5 | 0·2 | -3·045 |
|  |  | (25; 39) | (15; 24) | (0·3; 0·5) | (0·2; 0·2) | (-3·714; -2·398) |
| Piauí | Firearm death | 153 | 467 | 6·5 | 12·7 | 2·456 |
|  |  | (114·6; 190·3) | (417·4; 514·7) | (5·1; 8) | (11·3; 14) | (1·663; 3·289) |
|  | Homicide by firearm | 117 | 422 | 4·9 | 11·5 | 3·196 |
|  |  | (87; 148) | (376; 470) | (3·8; 6·2) | (10·2; 12·8) | (2·301; 4·172) |
|  | Suicide by firearm | 19 | 30 | 0·9 | 0·8 | -0·52 |
|  |  | (12; 26) | (21; 52) | (0·6; 1·2) | (0·6; 1·4) | (-2·467; 1·342) |
|  | Unintentional firearm death | 17 | 15 | 0·7 | 0·4 | -1·992 |
|  |  | (12; 21) | (12; 17) | (0·5; 0·8) | (0·3; 0·5) | (-2·745; -0·808) |
| Rio de Janeiro | Firearm death | 7142 | 5683 | 50·9 | 31·5 | -1·780 |
|  |  | (6271·8; 7468·3) | (4860·2; 5993·1) | (44·7; 53·2) | (26·9; 33·2) | (-2·022; -1·545) |
|  | Homicide by firearm | 6460 | 5448 | 45·7 | 30·2 | -1·515 |
|  |  | (5708; 6760) | (4660; 5748) | (40·2; 47·7) | (25·9; 31·9) | (-1·771; -1·279) |
|  | Suicide by firearm | 598 | 147 | 4·5 | 0·7 | -6·48 |
|  |  | (408; 742) | (93; 200) | (3·1; 5·6) | (0·5; 1·0) | (-7·023; -5·994) |
|  | Unintentional firearm death | 84 | 88 | 0·7 | 0·5 | -1·301 |
|  |  | (61; 92) | (70; 99) | (0·5; 0·7) | (0·4; 0·5) | (-1·756; -0·684) |
| Rio Grande do Norte | Firearm death | 250 | 1466 | 10·9 | 38·4 | 4·667 |
|  |  | (221·4; 279·2) | (1132·6; 1573·6) | (9·7; 12·2) | (29·6; 41·3) | (3·816; 5·136) |
|  | Homicide by firearm | 198 | 1401 | 8·5 | 36·7 | 5·55 |
|  |  | (174; 222) | (1076; 1510) | (7·5; 9·5) | (28·1; 39·6) | (4·612; 6·111) |
|  | Suicide by firearm | 27 | 34 | 1·3 | 0·9 | -1·527 |
|  |  | (20; 37) | (25; 55) | (1·0; 1·8) | (0·7; 1·4) | (-2·679; -0·325) |
|  | Unintentional firearm death | 25 | 31 | 1 | 0·8 | -0·819 |
|  |  | (19; 29) | (24; 35) | (0·8; 1·2) | (0·6; 0·9) | (-1·399; -0·240) |
| Rio Grande do Sul | Firearm death | 1455 | 2656 | 15·5 | 22·6 | 1·378 |
|  |  | (1358·4; 1604·5) | (2331·7; 2842·4) | (14·5; 17·2) | (19·7; 24·1) | (0·697; 1·698) |
|  | Homicide by firearm | 1121 | 2375 | 11·6 | 20·4 | 2·11 |
|  |  | (1045; 1200) | (1997; 2549) | (10·9; 12·4) | (17·1; 21·9) | (1·414; 2·422) |
|  | Suicide by firearm | 294 | 247 | 3·5 | 1·8 | -2·312 |
|  |  | (238; 414) | (183; 389) | (2·8; 4·9) | (1·4; 2·9) | (-2·929; -1·780) |
|  | Unintentional firearm death | 40 | 34 | 0·5 | 0·3 | -1·835 |
|  |  | (32; 44) | (28; 38) | (0·4; 0·5) | (0·2; 0·3) | (-2·346; -1·305) |
| Rondônia | Firearm death | 309 | 489 | 29·9 | 25·4 | -0·605 |
|  |  | (274·3; 345·6) | (396·7; 572·6) | (26·5; 33·5) | (20·6; 29·7) | (-1·378; 0·107) |
|  | Homicide by firearm | 270 | 462 | 25·7 | 23·9 | -0·279 |
|  |  | (238; 302) | (371; 540) | (22·6; 28·9) | (19·2; 27·9) | (-1·042; 0·441) |
|  | Suicide by firearm | 21 | 15 | 2·4 | 0·8 | -3·905 |
|  |  | (16; 30) | (10; 22) | (1·8; 3·4) | (0·6; 1·2) | (-4·684; -3·129) |
|  | Unintentional firearm death | 18 | 12 | 1·8 | 0·7 | -3·389 |
|  |  | (15; 30) | (9; 21) | (1·5; 3·0) | (0·5; 1·2) | (-4·082; -2·656) |
| Roraima | Firearm death | 22 | 69 | 10·7 | 11·5 | 0·274 |
|  |  | (18·2; 34·4) | (56·9; 91·8) | (8·9; 16·6) | (9·5; 15·4) | (-0·674; 1·095) |
|  | Homicide by firearm | 17 | 62 | 7·7 | 10·3 | 1·076 |
|  |  | (14; 27) | (51; 83) | (6·3; 12·7) | (8·4; 13·5) | (-0·022; 2·044) |
|  | Suicide by firearm | 3 | 4 | 1·8 | 0·7 | -3·437 |
|  |  | (2; 4) | (3; 6) | (1·3; 2·5) | (0·5; 1·1) | (-4·400; -2·489) |
|  | Unintentional firearm death | 2 | 3 | 1·2 | 0·6 | -2·807 |
|  |  | (2; 4) | (2; 6) | (1·0; 2·0) | (0·4; 1·0) | (-3·701; -1·921) |
| Santa Catarina | Firearm death | 311 | 679 | 7·1 | 8·9 | 0·843 |
|  |  | (281·5; 396·8) | (623·1; 755·7) | (6·4; 9·1) | (8·2; 10) | (0·217; 1·273) |
|  | Homicide by firearm | 218 | 600 | 4·7 | 7·9 | 1·926 |
|  |  | (199; 288) | (550; 655) | (4·3; 6·2) | (7·3; 8·7) | (1·026; 2·391) |
|  | Suicide by firearm | 76 | 66 | 2 | 0·8 | -3·157 |
|  |  | (60; 113) | (47; 109) | (1·6; 3·0) | (0·6; 1·4) | (-3·799; -2·579) |
|  | Unintentional firearm death | 17 | 13 | 0·4 | 0·2 | -2·865 |
|  |  | (15; 20) | (11; 18) | (0·4; 0·5) | (0·2; 0·2) | (-3·434; -2·163) |
| São Paulo | Firearm death | 4207 | 4656 | 12·5 | 9·7 | -0·937 |
|  |  | (3791·4; 7151·6) | (4324·8; 5170·6) | (11·3; 20·8) | (9; 10·7) | (-2·565; -0·49) |
|  | Homicide by firearm | 3755 | 4257 | 10·9 | 8·9 | -0·77 |
|  |  | (3346; 6794) | (3942; 4757) | (9·8; 19·6) | (8·2; 9·8) | (-2·613; -0·292) |
|  | Suicide by firearm | 328 | 260 | 1·1 | 0·5 | -2·85 |
|  |  | (235; 440) | (185; 368) | (0·8; 1·5) | (0·4; 0·7) | (-3·488; -1·886) |
|  | Unintentional firearm death | 124 | 139 | 0·4 | 0·3 | -1·271 |
|  |  | (95; 135) | (122; 156) | (0·3; 0·4) | (0·3; 0·3) | (-1·739; -0·360) |
| Sergipe | Firearm death | 255 | 1020 | 18·1 | 39·9 | 2·935 |
|  |  | (227·8; 278·8) | (816·1; 1105·2) | (16·1; 19·8) | (32; 43·2) | (2·189; 3·39) |
|  | Homicide by firearm | 227 | 996 | 16 | 38·9 | 3·343 |
|  |  | (200; 250) | (789; 1084) | (14·2; 17·6) | (30·8; 42·3) | (2·647; 3·854) |
|  | Suicide by firearm | 16 | 16 | 1·3 | 0·7 | -2·533 |
|  |  | (12; 22) | (12; 27) | (1·0; 1·8) | (0·5; 1·1) | (-3·488; -1·591) |
|  | Unintentional firearm death | 11 | 8 | 0·7 | 0·3 | -2·813 |
|  |  | (9; 13) | (7; 9) | (0·6; 0·9) | (0·3; 0·4) | (-3·560; -1·946) |
| Tocantins | Firearm death | 89 | 268 | 10·9 | 15·6 | 1·337 |
|  |  | (51·7; 117·9) | (232·5; 304·4) | (6·9; 14·2) | (13·6; 17·7) | (0·369; 2·831) |
|  | Homicide by firearm | 68 | 242 | 8·1 | 14 | 2·07 |
|  |  | (39; 91) | (208; 278) | (4·9; 10·6) | (12·0; 16·1) | (1·008; 3·755) |
|  | Suicide by firearm | 12 | 16 | 1·7 | 1 | -2·079 |
|  |  | (7; 17) | (12; 24) | (1·0; 2·4) | (0·7; 1·5) | (-3·571; -0·216) |
|  | Unintentional firearm death | 9 | 10 | 1·1 | 0·6 | -2·215 |
|  |  | (5; 12) | (8; 12) | (0·7; 1·4) | (0·5; 0·7) | (-3·225; -0·670) |
