# Supplementary material for: Association between firearms and mortality in Brazil, 1990 to 2017: a global burden of disease Brazil study
Source: Popul Health Metr. 2020 Sep 30;18(Suppl 1):19. doi: 10.1186/s12963-020-00222-3 (PMC7525968; doi:10.1186/s12963-020-00222-3)
Supplement: Supplementary file 3 — Additional file 3: Table S2. Median rates of total violent deaths related to firearms and firearms homicides, and median rates of voluntary return of firearms, according to two groups of states, the group with the highest rate of voluntary return of firearms and the group with the lowest rate of voluntary return of firearms, Brazil, 2000–2017. [file 12963_2020_222_MOESM3_ESM.docx]

| **Supplemental table  2**: Median rates of total violent deaths related to firearms and firearms homicides, and median rates of voluntary return of firearms, according to two groups of states, the group with the highest rate of voluntary return of firearms and the group with the lowest rate of voluntary return of firearms, Brazil, 2000–2017 | | | | | | |
| --- | --- | --- | --- | --- | --- | --- |
|  | **(group states median > 6)** | | | **group states median < 6** | | |
|  | **Median of rate Physical violence by firearm (group States > voluntary return of firearms)** | **Median of rate All firearms injuries (group States > voluntary return of firearms)** | **Median of rate of voluntary return of firearms** | **Median of rate Physical violence by firearm (group States < voluntary return of firearms)** | **Median of rate All firearms injuries (group States < voluntary return of firearms)** | **Median of rate of voluntary return of firearms** |
| 2000 | 27·9 | 29·3 |  | 14·0 | 15·8 |  |
| 2001 | 27·9 | 29·2 |  | 15·1 | 16·9 |  |
| 2002 | 26·9 | 28·1 |  | 16·6 | 18·0 |  |
| 2003 | 25·7 | 26·9 |  | 17·7 | 19·0 |  |
| 2004 | 23·6 | 24·6 |  | 18·7 | 20·0 |  |
| 2005 | 21,0 | 22·0 | 0·0 | 18·2 | 20·4 | 0·0 |
| 2006 | 20·3 | 21·3 | 2·0 | 17·8 | 19·9 | 0·0 |
| 2007 | 20·4 | 21·4 | 0·0 | 18·5 | 20·3 | 0·0 |
| 2008 | 20·4 | 22·0 | 5·0 | 19·5 | 21·5 | 1·0 |
| 2009 | 20·6 | 22·3 | 7·0 | 21·3 | 22·2 | 4·0 |
| 2010 | 20·5 | 22·0 | 7·0 | 23·9 | 25·1 | 2·0 |
| 2011 | 21·1 | 22·1 | 24·5 | 24·2 | 25·4 | 9·0 |
| 2012 | 21·3 | 22·6 | 13·0 | 23·8 | 25,0 | 5·0 |
| 2013 | 20·7 | 22·2 | 17·5 | 23·3 | 25·1 | 5·0 |
| 2014 | 20·7 | 22·2 | 12,0 | 24·1 | 25·5 | 2·0 |
| 2015 | 20·4 | 21·9 | 11·5 | 25·4 | 26·2 | 2·0 |
| 2016 | 20·7 | 22·3 | 8·0 | 25·0 | 25·8 | 1·0 |
| **2017** | **20·1** | **21·7** |  | **23·8** | **24·6** |  |
