# Supplementary material for: Association between firearms and mortality in Brazil, 1990 to 2017: a global burden of disease Brazil study
Source: Popul Health Metr. 2020 Sep 30;18(Suppl 1):19. doi: 10.1186/s12963-020-00222-3 (PMC7525968; doi:10.1186/s12963-020-00222-3)
Supplement: Supplementary file 4 — Additional file 4: Table S3. Rate per year of indicators, for Brazil and by state, 1990 to 2017. Legend: * [=1/(voluntary return of firearms+illegal carrying of firearms)]. [file 12963_2020_222_MOESM4_ESM.docx]

| **Supplemental table**  **3: Rate per year of indicators, for Brazil and by state, 1990 to 2017** | | | | | | | | | | | | | |
| --- | --- | --- | --- | --- | --- | --- | --- | --- | --- | --- | --- | --- | --- |
| Years | States | Unintentional firearm injuries | Self-harm | Self-harm by firearm | Interpersonal violence | Physical violence by firearm | All firearms injuries | SDI | Illegal carrying of firearms | Possession and use of narcotics | Narcotics trafficking | Voluntary return of firearms | Composite index* |
| 1990 | Acre | 1·3 | 5·4 | 1·7 | 25·7 | 10·8 | 13·9 | 0·376 | - | - | - | - | - |
| 1990 | Alagoas | 0·4 | 2·6 | 0·4 | 33·9 | 22·2 | 23·1 | 0·355 | - | - | - | - | - |
| 1990 | Amapá | 0·5 | 4·5 | 0·7 | 22·2 | 7·2 | 8·4 | 0·467 | - | - | - | - | - |
| 1990 | Amazonas | 0·6 | 3·9 | 0·7 | 22·5 | 10·5 | 11·8 | 0·438 | - | - | - | - | - |
| 1990 | Bahia | 4·4 | 2·2 | 0·4 | 12·7 | 7·1 | 12·0 | 0·402 | - | - | - | - | - |
| 1990 | Ceará | 0·5 | 4·5 | 0·7 | 17·2 | 8·8 | 10·0 | 0·411 | - | - | - | - | - |
| 1990 | Distrito Federal | 0·2 | 4·5 | 1·2 | 24·5 | 14·9 | 16·4 | 0·630 | - | - | - | - | - |
| 1990 | Espírito Santo | 0·4 | 4·3 | 0·9 | 35·7 | 18·3 | 19·6 | 0·499 | - | - | - | - | - |
| 1990 | Goiás | 0·7 | 8·5 | 2·4 | 27·5 | 15·1 | 18·2 | 0·460 | - | - | - | - | - |
| 1990 | Maranhão | 1·3 | 3·4 | 0·6 | 26·0 | 13·2 | 15·0 | 0·313 | - | - | - | - | - |
| 1990 | Mato Grosso | 3·6 | 4·4 | 1·2 | 25·3 | 3·8 | 8·6 | 0·475 | - | - | - | - | - |
| 1990 | Mato Grosso do Sul | 0·9 | 7·7 | 2·3 | 24·9 | 13·1 | 16·3 | 0·465 | - | - | - | - | - |
| 1990 | Minas Gerais | 0·4 | 6·7 | 1·5 | 11·8 | 5·8 | 7·6 | 0·491 | - | - | - | - | - |
| 1990 | Pará | 1·0 | 3·3 | 0·7 | 22·0 | 12·3 | 14·0 | 0·410 | - | - | - | - | - |
| 1990 | Paraíba | 0·4 | 3·2 | 0·4 | 22·4 | 15·4 | 16·2 | 0·399 | - | - | - | - | - |
| 1990 | Paraná | 0·4 | 7·9 | 1·8 | 17·1 | 8·6 | 10·8 | 0·513 | - | - | - | - | - |
| 1990 | Pernambuco | 0·5 | 5·3 | 1·0 | 48·9 | 32·2 | 33·7 | 0·416 | - | - | - | - | - |
| 1990 | Piauí | 0·7 | 4·0 | 0·7 | 10·1 | 4·4 | 5·7 | 0·365 | - | - | - | - | - |
| 1990 | Rio de Janeiro | 0·6 | 9·8 | 4·6 | 61·6 | 49·2 | 54·4 | 0·576 | - | - | - | - | - |
| 1990 | Rio Grande do Norte | 1·0 | 4·9 | 1·1 | 14·2 | 8·0 | 10·1 | 0·415 | - | - | - | - | - |
| 1990 | Rio Grande do Sul | 0·4 | 11·6 | 3·2 | 19·5 | 12·1 | 15·7 | 0·543 | - | - | - | - | - |
| 1990 | Rondônia | 1·6 | 7·2 | 1·9 | 44·9 | 24·2 | 27·7 | 0·423 | - | - | - | - | - |
| 1990 | Roraima | 1·2 | 7·4 | 1·4 | 32·6 | 8·0 | 10·5 | 0·428 | - | - | - | - | - |
| 1990 | Santa Catarina | 0·4 | 8·6 | 1·7 | 10·1 | 4·8 | 6·8 | 0·541 | - | - | - | - | - |
| 1990 | São Paulo | 0·4 | 6·1 | 1·0 | 31·7 | 11·7 | 13·1 | 0·558 | - | - | - | - | - |
| 1990 | Sergipe | 0·7 | 5·5 | 1·1 | 24·8 | 15·0 | 16·8 | 0·425 | - | - | - | - | - |
| 1990 | Tocantins | 1·0 | 4·7 | 1·3 | 17·5 | 7·4 | 9·7 | 0·396 | - | - | - | - | - |
| 1991 | Acre | 1·4 | 5·5 | 1·7 | 26·6 | 11·4 | 14·5 | 0·386 | - | - | - | - | - |
| 1991 | Alagoas | 0·4 | 2·6 | 0·4 | 32·6 | 21·4 | 22·2 | 0·363 | - | - | - | - | - |
| 1991 | Amapá | 0·5 | 4·6 | 0·7 | 24·2 | 8·1 | 9·4 | 0·475 | - | - | - | - | - |
| 1991 | Amazonas | 0·6 | 3·8 | 0·7 | 22·5 | 10·7 | 11·9 | 0·447 | - | - | - | - | - |
| 1991 | Bahia | 4·8 | 2·3 | 0·4 | 12·5 | 7·0 | 12·2 | 0·410 | - | - | - | - | - |
| 1991 | Ceará | 0·5 | 4·5 | 0·7 | 17·2 | 8·8 | 10·0 | 0·419 | - | - | - | - | - |
| 1991 | Distrito Federal | 0·2 | 4·7 | 1·3 | 25·5 | 15·9 | 17·4 | 0·636 | - | - | - | - | - |
| 1991 | Espírito Santo | 0·4 | 4·4 | 0·9 | 38·0 | 20·2 | 21·6 | 0·507 | - | - | - | - | - |
| 1991 | Goiás | 0·6 | 8·6 | 2·5 | 27·2 | 15·0 | 18·1 | 0·468 | - | - | - | - | - |
| 1991 | Maranhão | 1·2 | 3·2 | 0·5 | 23·9 | 12·2 | 14·0 | 0·322 | - | - | - | - | - |
| 1991 | Mato Grosso | 3·7 | 4·6 | 1·2 | 26·3 | 4·7 | 9·6 | 0·484 | - | - | - | - | - |
| 1991 | Mato Grosso do Sul | 0·9 | 7·4 | 2·2 | 25·4 | 13·7 | 16·9 | 0·473 | - | - | - | - | - |
| 1991 | Minas Gerais | 0·4 | 6·6 | 1·5 | 11·7 | 5·8 | 7·7 | 0·498 | - | - | - | - | - |
| 1991 | Pará | 1·0 | 3·3 | 0·7 | 22·0 | 12·4 | 14·0 | 0·418 | - | - | - | - | - |
| 1991 | Paraíba | 0·4 | 3·2 | 0·4 | 22·4 | 15·4 | 16·2 | 0·406 | - | - | - | - | - |
| 1991 | Paraná | 0·4 | 7·7 | 1·8 | 17·0 | 8·8 | 10·9 | 0·519 | - | - | - | - | - |
| 1991 | Pernambuco | 0·4 | 5·2 | 1·0 | 47·4 | 31·7 | 33·2 | 0·423 | - | - | - | - | - |
| 1991 | Piauí | 0·6 | 3·8 | 0·7 | 9·3 | 4·0 | 5·3 | 0·372 | - | - | - | - | - |
| 1991 | Rio de Janeiro | 0·6 | 9·4 | 4·4 | 59·5 | 48·0 | 53·0 | 0·581 | - | - | - | - | - |
| 1991 | Rio Grande do Norte | 1·0 | 5·2 | 1·1 | 14·9 | 8·3 | 10·5 | 0·422 | - | - | - | - | - |
| 1991 | Rio Grande do Sul | 0·4 | 11·4 | 3·1 | 19·3 | 12·3 | 15·8 | 0·549 | - | - | - | - | - |
| 1991 | Rondônia | 1·5 | 7·0 | 1·8 | 42·9 | 23·6 | 27·0 | 0·433 | - | - | - | - | - |
| 1991 | Roraima | 1·2 | 7·4 | 1·4 | 32·9 | 8·7 | 11·2 | 0·438 | - | - | - | - | - |
| 1991 | Santa Catarina | 0·4 | 8·5 | 1·7 | 9·8 | 4·7 | 6·8 | 0·548 | - | - | - | - | - |
| 1991 | São Paulo | 0·4 | 5·9 | 1·0 | 30·9 | 11·9 | 13·2 | 0·565 | - | - | - | - | - |
| 1991 | Sergipe | 0·7 | 6·0 | 1·2 | 27·0 | 16·4 | 18·3 | 0·433 | - | - | - | - | - |
| 1991 | Tocantins | 1·0 | 4·8 | 1·3 | 17·6 | 7·4 | 9·7 | 0·404 | - | - | - | - | - |
| 1992 | Acre | 1·3 | 5·6 | 1·7 | 26·8 | 11·7 | 14·7 | 0·395 | - | - | - | - | - |
| 1992 | Alagoas | 0·4 | 2·6 | 0·4 | 31·5 | 20·7 | 21·5 | 0·371 | - | - | - | - | - |
| 1992 | Amapá | 0·5 | 4·7 | 0·8 | 25·9 | 9·3 | 10·6 | 0·483 | - | - | - | - | - |
| 1992 | Amazonas | 0·5 | 3·9 | 0·7 | 22·2 | 10·5 | 11·8 | 0·457 | - | - | - | - | - |
| 1992 | Bahia | 4·1 | 2·3 | 0·4 | 13·8 | 8·1 | 12·6 | 0·419 | - | - | - | - | - |
| 1992 | Ceará | 0·5 | 4·6 | 0·7 | 17·2 | 8·8 | 10·0 | 0·426 | - | - | - | - | - |
| 1992 | Distrito Federal | 0·2 | 4·8 | 1·4 | 26·3 | 16·8 | 18·4 | 0·642 | - | - | - | - | - |
| 1992 | Espírito Santo | 0·4 | 4·4 | 0·9 | 39·9 | 22·0 | 23·4 | 0·515 | - | - | - | - | - |
| 1992 | Goiás | 0·6 | 8·6 | 2·5 | 26·6 | 14·9 | 17·9 | 0·476 | - | - | - | - | - |
| 1992 | Maranhão | 1·1 | 3·2 | 0·5 | 23·3 | 12·0 | 13·7 | 0·330 | - | - | - | - | - |
| 1992 | Mato Grosso | 3·5 | 4·7 | 1·3 | 26·3 | 5·5 | 10·2 | 0·492 | - | - | - | - | - |
| 1992 | Mato Grosso do Sul | 0·9 | 7·7 | 2·3 | 26·3 | 14·6 | 17·8 | 0·481 | - | - | - | - | - |
| 1992 | Minas Gerais | 0·4 | 6·8 | 1·5 | 11·9 | 6·0 | 7·9 | 0·506 | - | - | - | - | - |
| 1992 | Pará | 0·9 | 3·2 | 0·7 | 21·4 | 12·2 | 13·8 | 0·425 | - | - | - | - | - |
| 1992 | Paraíba | 0·4 | 3·3 | 0·4 | 22·4 | 15·4 | 16·2 | 0·413 | - | - | - | - | - |
| 1992 | Paraná | 0·4 | 7·7 | 1·8 | 16·9 | 9·0 | 11·1 | 0·525 | - | - | - | - | - |
| 1992 | Pernambuco | 0·4 | 5·0 | 1·0 | 43·9 | 29·8 | 31·2 | 0·430 | - | - | - | - | - |
| 1992 | Piauí | 0·6 | 3·9 | 0·7 | 9·2 | 4·0 | 5·2 | 0·379 | - | - | - | - | - |
| 1992 | Rio de Janeiro | 0·6 | 9·1 | 4·1 | 59·4 | 48·5 | 53·2 | 0·585 | - | - | - | - | - |
| 1992 | Rio Grande do Norte | 1·0 | 5·3 | 1·2 | 15·0 | 8·5 | 10·7 | 0·429 | - | - | - | - | - |
| 1992 | Rio Grande do Sul | 0·4 | 11·4 | 3·2 | 19·1 | 12·4 | 16·0 | 0·555 | - | - | - | - | - |
| 1992 | Rondônia | 1·4 | 6·8 | 1·7 | 40·2 | 22·8 | 26·0 | 0·441 | - | - | - | - | - |
| 1992 | Roraima | 1·1 | 7·0 | 1·3 | 32·4 | 9·3 | 11·7 | 0·447 | - | - | - | - | - |
| 1992 | Santa Catarina | 0·4 | 8·5 | 1·7 | 9·8 | 4·8 | 6·8 | 0·554 | - | - | - | - | - |
| 1992 | São Paulo | 0·4 | 5·8 | 1·0 | 30·6 | 12·2 | 13·6 | 0·572 | - | - | - | - | - |
| 1992 | Sergipe | 0·7 | 6·5 | 1·3 | 29·1 | 17·7 | 19·7 | 0·441 | - | - | - | - | - |
| 1992 | Tocantins | 0·9 | 4·9 | 1·3 | 17·6 | 7·4 | 9·6 | 0·412 | - | - | - | - | - |
| 1993 | Acre | 1·2 | 5·7 | 1·7 | 27·4 | 12·3 | 15·2 | 0·405 | - | - | - | - | - |
| 1993 | Alagoas | 0·3 | 2·6 | 0·4 | 31·0 | 20·4 | 21·2 | 0·379 | - | - | - | - | - |
| 1993 | Amapá | 0·5 | 4·8 | 0·8 | 27·8 | 10·5 | 11·8 | 0·491 | - | - | - | - | - |
| 1993 | Amazonas | 0·5 | 3·9 | 0·7 | 22·1 | 10·5 | 11·7 | 0·466 | - | - | - | - | - |
| 1993 | Bahia | 3·0 | 2·4 | 0·5 | 15·4 | 9·8 | 13·3 | 0·427 | - | - | - | - | - |
| 1993 | Ceará | 0·4 | 4·9 | 0·7 | 17·9 | 9·2 | 10·4 | 0·433 | - | - | - | - | - |
| 1993 | Distrito Federal | 0·2 | 5·0 | 1·4 | 29·0 | 18·9 | 20·5 | 0·649 | - | - | - | - | - |
| 1993 | Espírito Santo | 0·4 | 4·6 | 1·0 | 42·8 | 24·9 | 26·3 | 0·524 | - | - | - | - | - |
| 1993 | Goiás | 0·6 | 8·8 | 2·6 | 26·5 | 15·1 | 18·3 | 0·484 | - | - | - | - | - |
| 1993 | Maranhão | 1·1 | 3·1 | 0·5 | 22·3 | 11·6 | 13·1 | 0·339 | - | - | - | - | - |
| 1993 | Mato Grosso | 3·1 | 4·8 | 1·3 | 26·4 | 5·7 | 10·1 | 0·501 | - | - | - | - | - |
| 1993 | Mato Grosso do Sul | 0·8 | 8·1 | 2·5 | 28·4 | 16·3 | 19·6 | 0·489 | - | - | - | - | - |
| 1993 | Minas Gerais | 0·4 | 6·8 | 1·5 | 12·1 | 6·3 | 8·1 | 0·513 | - | - | - | - | - |
| 1993 | Pará | 0·9 | 3·2 | 0·6 | 20·7 | 11·9 | 13·4 | 0·432 | - | - | - | - | - |
| 1993 | Paraíba | 0·4 | 3·4 | 0·4 | 23·0 | 15·9 | 16·7 | 0·420 | - | - | - | - | - |
| 1993 | Paraná | 0·4 | 7·9 | 1·8 | 17·8 | 9·7 | 11·9 | 0·532 | - | - | - | - | - |
| 1993 | Pernambuco | 0·4 | 5·1 | 1·0 | 44·3 | 30·8 | 32·2 | 0·437 | - | - | - | - | - |
| 1993 | Piauí | 0·5 | 3·9 | 0·7 | 9·0 | 3·9 | 5·1 | 0·386 | - | - | - | - | - |
| 1993 | Rio de Janeiro | 0·7 | 8·7 | 3·7 | 67·5 | 49·6 | 53·9 | 0·590 | - | - | - | - | - |
| 1993 | Rio Grande do Norte | 0·9 | 5·7 | 1·2 | 15·5 | 9·0 | 11·2 | 0·436 | - | - | - | - | - |
| 1993 | Rio Grande do Sul | 0·4 | 11·8 | 3·4 | 19·3 | 12·7 | 16·6 | 0·561 | - | - | - | - | - |
| 1993 | Rondônia | 1·3 | 6·8 | 1·7 | 40·0 | 23·1 | 26·1 | 0·450 | - | - | - | - | - |
| 1993 | Roraima | 1·0 | 7·1 | 1·3 | 32·2 | 10·0 | 12·3 | 0·456 | - | - | - | - | - |
| 1993 | Santa Catarina | 0·3 | 8·8 | 1·7 | 10·1 | 5·1 | 7·1 | 0·560 | - | - | - | - | - |
| 1993 | São Paulo | 0·4 | 6·0 | 1·1 | 31·5 | 13·6 | 15·0 | 0·579 | - | - | - | - | - |
| 1993 | Sergipe | 0·7 | 6·6 | 1·2 | 27·6 | 16·8 | 18·7 | 0·448 | - | - | - | - | - |
| 1993 | Tocantins | 0·9 | 5·0 | 1·3 | 17·7 | 7·2 | 9·4 | 0·420 | - | - | - | - | - |
| 1994 | Acre | 1·1 | 5·8 | 1·7 | 27·4 | 12·5 | 15·4 | 0·415 | - | - | - | - | - |
| 1994 | Alagoas | 0·3 | 2·6 | 0·4 | 31·5 | 20·8 | 21·6 | 0·387 | - | - | - | - | - |
| 1994 | Amapá | 0·5 | 4·9 | 0·8 | 30·7 | 11·8 | 13·1 | 0·500 | - | - | - | - | - |
| 1994 | Amazonas | 0·5 | 3·9 | 0·7 | 22·3 | 10·6 | 11·8 | 0·475 | - | - | - | - | - |
| 1994 | Bahia | 2·3 | 2·5 | 0·5 | 16·8 | 11·2 | 13·9 | 0·435 | - | - | - | - | - |
| 1994 | Ceará | 0·4 | 5·1 | 0·7 | 18·3 | 9·4 | 10·6 | 0·440 | - | - | - | - | - |
| 1994 | Distrito Federal | 0·2 | 5·2 | 1·5 | 30·8 | 20·5 | 22·2 | 0·656 | - | - | - | - | - |
| 1994 | Espírito Santo | 0·4 | 4·6 | 1·0 | 44·9 | 27·8 | 29·2 | 0·532 | - | - | - | - | - |
| 1994 | Goiás | 0·6 | 9·3 | 2·6 | 26·9 | 15·7 | 18·9 | 0·493 | - | - | - | - | - |
| 1994 | Maranhão | 1·0 | 3·0 | 0·5 | 20·7 | 10·8 | 12·3 | 0·347 | - | - | - | - | - |
| 1994 | Mato Grosso | 2·8 | 5·0 | 1·3 | 26·9 | 7·8 | 11·9 | 0·509 | - | - | - | - | - |
| 1994 | Mato Grosso do Sul | 0·8 | 8·7 | 2·6 | 31·5 | 18·7 | 22·1 | 0·497 | - | - | - | - | - |
| 1994 | Minas Gerais | 0·4 | 6·6 | 1·4 | 11·8 | 6·3 | 8·1 | 0·521 | - | - | - | - | - |
| 1994 | Pará | 0·8 | 3·1 | 0·6 | 20·5 | 11·9 | 13·3 | 0·440 | - | - | - | - | - |
| 1994 | Paraíba | 0·4 | 3·5 | 0·4 | 23·8 | 16·5 | 17·3 | 0·427 | - | - | - | - | - |
| 1994 | Paraná | 0·4 | 8·0 | 1·9 | 18·5 | 10·4 | 12·7 | 0·539 | - | - | - | - | - |
| 1994 | Pernambuco | 0·4 | 5·0 | 1·0 | 43·9 | 31·4 | 32·7 | 0·444 | - | - | - | - | - |
| 1994 | Piauí | 0·5 | 3·8 | 0·6 | 8·9 | 3·9 | 5·0 | 0·393 | - | - | - | - | - |
| 1994 | Rio de Janeiro | 0·7 | 7·9 | 3·1 | 74·8 | 49·6 | 53·4 | 0·595 | - | - | - | - | - |
| 1994 | Rio Grande do Norte | 0·9 | 6·0 | 1·3 | 16·0 | 9·5 | 11·7 | 0·444 | - | - | - | - | - |
| 1994 | Rio Grande do Sul | 0·4 | 11·8 | 3·5 | 19·3 | 13·0 | 16·9 | 0·567 | - | - | - | - | - |
| 1994 | Rondônia | 1·2 | 6·8 | 1·7 | 39·3 | 23·2 | 26·1 | 0·458 | - | - | - | - | - |
| 1994 | Roraima | 1·0 | 7·2 | 1·3 | 32·6 | 10·7 | 13·0 | 0·465 | - | - | - | - | - |
| 1994 | Santa Catarina | 0·3 | 8·9 | 1·8 | 10·1 | 5·2 | 7·3 | 0·567 | - | - | - | - | - |
| 1994 | São Paulo | 0·4 | 6·2 | 1·1 | 33·8 | 15·6 | 17·2 | 0·587 | - | - | - | - | - |
| 1994 | Sergipe | 0·6 | 6·9 | 1·3 | 28·0 | 17·1 | 18·9 | 0·456 | - | - | - | - | - |
| 1994 | Tocantins | 0·9 | 5·0 | 1·3 | 17·5 | 7·5 | 9·7 | 0·428 | - | - | - | - | - |
| 1995 | Acre | 1·1 | 5·8 | 1·7 | 27·7 | 13·1 | 15·9 | 0·424 | - | - | - | - | - |
| 1995 | Alagoas | 0·3 | 2·8 | 0·4 | 32·5 | 21·4 | 22·1 | 0·395 | - | - | - | - | - |
| 1995 | Amapá | 0·5 | 5·1 | 0·8 | 32·8 | 13·0 | 14·3 | 0·508 | - | - | - | - | - |
| 1995 | Amazonas | 0·4 | 3·9 | 0·7 | 22·5 | 10·7 | 11·8 | 0·483 | - | - | - | - | - |
| 1995 | Bahia | 2·0 | 2·6 | 0·5 | 17·7 | 12·3 | 14·8 | 0·443 | - | - | - | - | - |
| 1995 | Ceará | 0·4 | 5·4 | 0·8 | 19·3 | 9·8 | 11·0 | 0·448 | - | - | - | - | - |
| 1995 | Distrito Federal | 0·2 | 5·2 | 1·5 | 31·4 | 21·5 | 23·2 | 0·663 | - | - | - | - | - |
| 1995 | Espírito Santo | 0·4 | 4·6 | 0·9 | 45·5 | 30·4 | 31·8 | 0·540 | - | - | - | - | - |
| 1995 | Goiás | 0·6 | 9·3 | 2·6 | 26·5 | 15·9 | 19·1 | 0·501 | - | - | - | - | - |
| 1995 | Maranhão | 0·9 | 2·9 | 0·5 | 19·5 | 10·3 | 11·6 | 0·355 | - | - | - | - | - |
| 1995 | Mato Grosso | 2·5 | 5·0 | 1·3 | 27·8 | 10·9 | 14·6 | 0·518 | - | - | - | - | - |
| 1995 | Mato Grosso do Sul | 0·9 | 8·9 | 2·6 | 34·5 | 21·5 | 25·0 | 0·506 | - | - | - | - | - |
| 1995 | Minas Gerais | 0·4 | 6·5 | 1·4 | 12·0 | 6·7 | 8·5 | 0·530 | - | - | - | - | - |
| 1995 | Pará | 0·8 | 3·2 | 0·6 | 20·1 | 11·8 | 13·2 | 0·447 | - | - | - | - | - |
| 1995 | Paraíba | 0·4 | 3·7 | 0·4 | 25·3 | 17·6 | 18·4 | 0·434 | - | - | - | - | - |
| 1995 | Paraná | 0·4 | 8·0 | 1·9 | 19·0 | 11·0 | 13·3 | 0·548 | - | - | - | - | - |
| 1995 | Pernambuco | 0·4 | 5·1 | 1·0 | 45·1 | 33·1 | 34·5 | 0·451 | - | - | - | - | - |
| 1995 | Piauí | 0·5 | 3·8 | 0·6 | 8·7 | 3·8 | 4·9 | 0·400 | - | - | - | - | - |
| 1995 | Rio de Janeiro | 0·6 | 6·7 | 2·3 | 84·5 | 46·8 | 49·7 | 0·601 | - | - | - | - | - |
| 1995 | Rio Grande do Norte | 1·0 | 6·5 | 1·3 | 17·2 | 10·4 | 12·7 | 0·451 | - | - | - | - | - |
| 1995 | Rio Grande do Sul | 0·4 | 11·7 | 3·5 | 19·2 | 13·2 | 17·2 | 0·574 | - | - | - | - | - |
| 1995 | Rondônia | 1·3 | 6·9 | 1·7 | 38·7 | 23·4 | 26·3 | 0·467 | - | - | - | - | - |
| 1995 | Roraima | 1·0 | 7·1 | 1·3 | 32·8 | 11·2 | 13·4 | 0·474 | - | - | - | - | - |
| 1995 | Santa Catarina | 0·3 | 9·2 | 1·8 | 10·6 | 5·6 | 7·7 | 0·574 | - | - | - | - | - |
| 1995 | São Paulo | 0·5 | 6·4 | 1·1 | 37·3 | 18·6 | 20·3 | 0·595 | - | - | - | - | - |
| 1995 | Sergipe | 0·6 | 7·5 | 1·3 | 28·9 | 17·6 | 19·5 | 0·464 | - | - | - | - | - |
| 1995 | Tocantins | 0·8 | 5·2 | 1·3 | 17·5 | 7·6 | 9·8 | 0·436 | - | - | - | - | - |
| 1996 | Acre | 1·2 | 5·8 | 1·6 | 28·0 | 13·7 | 16·4 | 0·435 | - | - | - | - | - |
| 1996 | Alagoas | 0·3 | 2·8 | 0·4 | 32·0 | 21·4 | 22·2 | 0·404 | - | - | - | - | - |
| 1996 | Amapá | 0·6 | 5·2 | 0·8 | 34·8 | 14·3 | 15·6 | 0·517 | - | - | - | - | - |
| 1996 | Amazonas | 0·4 | 3·8 | 0·6 | 21·8 | 10·5 | 11·5 | 0·492 | - | - | - | - | - |
| 1996 | Bahia | 1·8 | 2·8 | 0·5 | 18·9 | 13·8 | 16·1 | 0·451 | - | - | - | - | - |
| 1996 | Ceará | 0·5 | 5·6 | 0·8 | 20·0 | 10·3 | 11·6 | 0·455 | - | - | - | - | - |
| 1996 | Distrito Federal | 0·2 | 5·1 | 1·4 | 31·2 | 21·7 | 23·3 | 0·671 | - | - | - | - | - |
| 1996 | Espírito Santo | 0·4 | 4·8 | 0·9 | 48·8 | 36·2 | 37·5 | 0·549 | - | - | - | - | - |
| 1996 | Goiás | 0·6 | 8·8 | 2·4 | 25·2 | 15·6 | 18·6 | 0·510 | - | - | - | - | - |
| 1996 | Maranhão | 0·8 | 2·8 | 0·4 | 18·8 | 9·9 | 11·2 | 0·364 | - | - | - | - | - |
| 1996 | Mato Grosso | 2·2 | 4·9 | 1·2 | 28·2 | 13·4 | 16·8 | 0·527 | - | - | - | - | - |
| 1996 | Mato Grosso do Sul | 0·9 | 8·8 | 2·5 | 36·0 | 23·2 | 26·6 | 0·515 | - | - | - | - | - |
| 1996 | Minas Gerais | 0·5 | 6·5 | 1·4 | 12·6 | 7·4 | 9·2 | 0·538 | - | - | - | - | - |
| 1996 | Pará | 0·8 | 3·2 | 0·6 | 19·9 | 12·0 | 13·4 | 0·454 | - | - | - | - | - |
| 1996 | Paraíba | 0·4 | 3·7 | 0·4 | 25·5 | 17·9 | 18·7 | 0·441 | - | - | - | - | - |
| 1996 | Paraná | 0·4 | 8·1 | 1·9 | 19·5 | 11·6 | 13·8 | 0·556 | - | - | - | - | - |
| 1996 | Pernambuco | 0·4 | 5·1 | 1·0 | 48·2 | 36·3 | 37·7 | 0·458 | - | - | - | - | - |
| 1996 | Piauí | 0·5 | 3·8 | 0·6 | 8·5 | 3·8 | 4·9 | 0·408 | - | - | - | - | - |
| 1996 | Rio de Janeiro | 0·7 | 6·0 | 1·8 | 76·7 | 45·3 | 47·8 | 0·608 | - | - | - | - | - |
| 1996 | Rio Grande do Norte | 1·1 | 6·6 | 1·3 | 18·0 | 11·2 | 13·6 | 0·460 | - | - | - | - | - |
| 1996 | Rio Grande do Sul | 0·4 | 11·8 | 3·5 | 19·2 | 13·6 | 17·6 | 0·581 | - | - | - | - | - |
| 1996 | Rondônia | 1·3 | 6·7 | 1·6 | 37·6 | 23·6 | 26·5 | 0·475 | - | - | - | - | - |
| 1996 | Roraima | 1·0 | 7·1 | 1·2 | 32·8 | 11·4 | 13·6 | 0·483 | - | - | - | - | - |
| 1996 | Santa Catarina | 0·3 | 9·3 | 1·8 | 10·7 | 5·8 | 7·9 | 0·582 | - | - | - | - | - |
| 1996 | São Paulo | 0·5 | 6·5 | 1·1 | 39·2 | 21·4 | 23·1 | 0·603 | - | - | - | - | - |
| 1996 | Sergipe | 0·7 | 7·0 | 1·2 | 28·2 | 17·5 | 19·4 | 0·473 | - | - | - | - | - |
| 1996 | Tocantins | 0·9 | 5·2 | 1·2 | 17·9 | 8·2 | 10·3 | 0·445 | - | - | - | - | - |
| 1997 | Acre | 1·1 | 5·7 | 1·5 | 27·6 | 13·6 | 16·2 | 0·445 | - | - | - | - | - |
| 1997 | Alagoas | 0·3 | 2·9 | 0·4 | 31·9 | 21·6 | 22·4 | 0·412 | - | - | - | - | - |
| 1997 | Amapá | 0·6 | 5·2 | 0·7 | 35·6 | 14·6 | 15·9 | 0·526 | - | - | - | - | - |
| 1997 | Amazonas | 0·4 | 3·8 | 0·6 | 22·0 | 10·5 | 11·6 | 0·499 | - | - | - | - | - |
| 1997 | Bahia | 1·9 | 3·0 | 0·6 | 19·7 | 14·5 | 17·0 | 0·459 | - | - | - | - | - |
| 1997 | Ceará | 0·5 | 5·7 | 0·8 | 20·9 | 11·1 | 12·3 | 0·463 | - | - | - | - | - |
| 1997 | Distrito Federal | 0·2 | 5·0 | 1·3 | 31·7 | 22·5 | 24·0 | 0·679 | - | - | - | - | - |
| 1997 | Espírito Santo | 0·4 | 4·8 | 0·9 | 52·1 | 40·5 | 41·8 | 0·557 | - | - | - | - | - |
| 1997 | Goiás | 0·6 | 8·9 | 2·3 | 25·2 | 15·8 | 18·7 | 0·518 | - | - | - | - | - |
| 1997 | Maranhão | 0·8 | 2·9 | 0·4 | 18·8 | 9·9 | 11·2 | 0·371 | - | - | - | - | - |
| 1997 | Mato Grosso | 2·0 | 5·0 | 1·2 | 29·8 | 15·6 | 18·8 | 0·535 | - | - | - | - | - |
| 1997 | Mato Grosso do Sul | 0·8 | 8·5 | 2·4 | 35·8 | 23·5 | 26·7 | 0·523 | - | - | - | - | - |
| 1997 | Minas Gerais | 0·5 | 6·7 | 1·3 | 13·6 | 8·3 | 10·1 | 0·545 | - | - | - | - | - |
| 1997 | Pará | 0·9 | 3·3 | 0·6 | 20·5 | 12·6 | 14·0 | 0·461 | - | - | - | - | - |
| 1997 | Paraíba | 0·3 | 3·8 | 0·4 | 25·1 | 17·7 | 18·4 | 0·447 | - | - | - | - | - |
| 1997 | Paraná | 0·4 | 8·0 | 1·8 | 20·0 | 12·1 | 14·3 | 0·564 | - | - | - | - | - |
| 1997 | Pernambuco | 0·4 | 5·3 | 1·0 | 53·7 | 41·6 | 42·9 | 0·466 | - | - | - | - | - |
| 1997 | Piauí | 0·5 | 4·0 | 0·7 | 8·8 | 3·9 | 5·1 | 0·415 | - | - | - | - | - |
| 1997 | Rio de Janeiro | 0·7 | 5·5 | 1·5 | 75·5 | 44·5 | 46·7 | 0·614 | - | - | - | - | - |
| 1997 | Rio Grande do Norte | 1·1 | 6·8 | 1·3 | 18·2 | 11·5 | 13·9 | 0·467 | - | - | - | - | - |
| 1997 | Rio Grande do Sul | 0·4 | 11·8 | 3·5 | 19·0 | 13·7 | 17·6 | 0·587 | - | - | - | - | - |
| 1997 | Rondônia | 1·3 | 6·5 | 1·5 | 38·2 | 24·3 | 27·0 | 0·484 | - | - | - | - | - |
| 1997 | Roraima | 1·0 | 7·1 | 1·1 | 32·4 | 11·6 | 13·7 | 0·492 | - | - | - | - | - |
| 1997 | Santa Catarina | 0·3 | 9·2 | 1·7 | 10·4 | 5·7 | 7·8 | 0·589 | - | - | - | - | - |
| 1997 | São Paulo | 0·5 | 6·3 | 1·1 | 39·6 | 23·2 | 24·8 | 0·611 | - | - | - | - | - |
| 1997 | Sergipe | 0·6 | 6·8 | 1·1 | 27·1 | 17·0 | 18·8 | 0·481 | - | - | - | - | - |
| 1997 | Tocantins | 0·9 | 5·2 | 1·2 | 17·9 | 8·4 | 10·5 | 0·453 | - | - | - | - | - |
| 1998 | Acre | 1·1 | 5·7 | 1·5 | 27·4 | 13·5 | 16·1 | 0·453 | - | - | - | - | - |
| 1998 | Alagoas | 0·3 | 3·0 | 0·4 | 31·8 | 21·5 | 22·3 | 0·419 | - | - | - | - | - |
| 1998 | Amapá | 0·6 | 5·2 | 0·7 | 36·6 | 14·8 | 16·1 | 0·534 | - | - | - | - | - |
| 1998 | Amazonas | 0·4 | 3·9 | 0·6 | 22·4 | 10·6 | 11·6 | 0·505 | - | - | - | - | - |
| 1998 | Bahia | 2·5 | 3·4 | 0·7 | 20·2 | 14·9 | 18·0 | 0·465 | - | - | - | - | - |
| 1998 | Ceará | 0·5 | 6·0 | 0·8 | 21·4 | 11·5 | 12·8 | 0·469 | - | - | - | - | - |
| 1998 | Distrito Federal | 0·2 | 5·0 | 1·3 | 32·2 | 23·2 | 24·7 | 0·685 | - | - | - | - | - |
| 1998 | Espírito Santo | 0·4 | 4·9 | 0·9 | 54·7 | 43·6 | 44·9 | 0·564 | - | - | - | - | - |
| 1998 | Goiás | 0·7 | 8·8 | 2·2 | 25·2 | 16·1 | 18·9 | 0·526 | - | - | - | - | - |
| 1998 | Maranhão | 0·8 | 3·0 | 0·4 | 18·6 | 9·8 | 11·0 | 0·377 | - | - | - | - | - |
| 1998 | Mato Grosso | 1·9 | 5·4 | 1·3 | 32·2 | 18·2 | 21·3 | 0·543 | - | - | - | - | - |
| 1998 | Mato Grosso do Sul | 0·8 | 8·1 | 2·2 | 34·0 | 22·4 | 25·4 | 0·531 | - | - | - | - | - |
| 1998 | Minas Gerais | 0·6 | 6·6 | 1·2 | 14·5 | 9·2 | 11·0 | 0·551 | - | - | - | - | - |
| 1998 | Pará | 1·0 | 3·5 | 0·6 | 21·3 | 13·2 | 14·7 | 0·466 | - | - | - | - | - |
| 1998 | Paraíba | 0·3 | 3·8 | 0·5 | 25·0 | 17·5 | 18·3 | 0·453 | - | - | - | - | - |
| 1998 | Paraná | 0·4 | 7·9 | 1·7 | 20·3 | 12·5 | 14·6 | 0·572 | - | - | - | - | - |
| 1998 | Pernambuco | 0·4 | 5·3 | 0·9 | 57·9 | 45·7 | 47·0 | 0·472 | - | - | - | - | - |
| 1998 | Piauí | 0·5 | 4·4 | 0·7 | 9·4 | 4·2 | 5·4 | 0·420 | - | - | - | - | - |
| 1998 | Rio de Janeiro | 0·8 | 5·4 | 1·4 | 72·3 | 44·2 | 46·4 | 0·619 | - | - | - | - | - |
| 1998 | Rio Grande do Norte | 1·1 | 6·7 | 1·2 | 17·5 | 11·1 | 13·5 | 0·474 | - | - | - | - | - |
| 1998 | Rio Grande do Sul | 0·5 | 12·0 | 3·4 | 18·8 | 13·7 | 17·6 | 0·593 | - | - | - | - | - |
| 1998 | Rondônia | 1·3 | 6·5 | 1·4 | 39·9 | 25·9 | 28·7 | 0·491 | - | - | - | - | - |
| 1998 | Roraima | 1·1 | 7·2 | 1·1 | 32·8 | 11·9 | 14·1 | 0·499 | - | - | - | - | - |
| 1998 | Santa Catarina | 0·3 | 9·0 | 1·6 | 10·0 | 5·5 | 7·5 | 0·595 | - | - | - | - | - |
| 1998 | São Paulo | 0·5 | 6·2 | 1·0 | 40·7 | 25·9 | 27·4 | 0·618 | - | - | - | - | - |
| 1998 | Sergipe | 0·7 | 6·8 | 1·1 | 27·3 | 17·5 | 19·2 | 0·488 | - | - | - | - | - |
| 1998 | Tocantins | 0·9 | 5·4 | 1·2 | 18·2 | 8·8 | 10·9 | 0·460 | - | - | - | - | - |
| 1999 | Acre | 1·1 | 5·5 | 1·4 | 26·4 | 12·8 | 15·3 | 0·460 | - | - | - | - | - |
| 1999 | Alagoas | 0·3 | 3·0 | 0·4 | 31·9 | 21·7 | 22·4 | 0·425 | - | - | - | - | - |
| 1999 | Amapá | 0·6 | 5·3 | 0·7 | 37·9 | 14·7 | 16·0 | 0·540 | - | - | - | - | - |
| 1999 | Amazonas | 0·4 | 3·9 | 0·6 | 22·7 | 10·6 | 11·6 | 0·510 | - | - | - | - | - |
| 1999 | Bahia | 2·8 | 3·6 | 0·7 | 20·1 | 14·8 | 18·4 | 0·470 | - | - | - | - | - |
| 1999 | Ceará | 0·5 | 6·2 | 0·8 | 22·1 | 12·0 | 13·3 | 0·475 | - | - | - | - | - |
| 1999 | Distrito Federal | 0·2 | 4·8 | 1·2 | 32·8 | 23·7 | 25·1 | 0·691 | - | - | - | - | - |
| 1999 | Espírito Santo | 0·4 | 4·7 | 0·8 | 52·9 | 42·7 | 44·0 | 0·570 | - | - | - | - | - |
| 1999 | Goiás | 0·6 | 8·4 | 2·0 | 25·2 | 16·3 | 19·0 | 0·532 | - | - | - | - | - |
| 1999 | Maranhão | 0·7 | 3·0 | 0·4 | 18·1 | 9·6 | 10·8 | 0·380 | - | - | - | - | - |
| 1999 | Mato Grosso | 1·7 | 5·5 | 1·2 | 33·4 | 19·6 | 22·6 | 0·548 | - | - | - | - | - |
| 1999 | Mato Grosso do Sul | 0·7 | 8·1 | 2·1 | 32·0 | 21·0 | 23·8 | 0·537 | - | - | - | - | - |
| 1999 | Minas Gerais | 0·6 | 6·4 | 1·1 | 15·2 | 9·9 | 11·5 | 0·557 | - | - | - | - | - |
| 1999 | Pará | 1·0 | 3·5 | 0·6 | 21·7 | 13·5 | 15·1 | 0·470 | - | - | - | - | - |
| 1999 | Paraíba | 0·3 | 3·9 | 0·5 | 25·0 | 17·6 | 18·4 | 0·457 | - | - | - | - | - |
| 1999 | Paraná | 0·4 | 7·6 | 1·6 | 20·7 | 13·0 | 15·0 | 0·578 | - | - | - | - | - |
| 1999 | Pernambuco | 0·4 | 5·3 | 0·9 | 58·0 | 46·3 | 47·6 | 0·477 | - | - | - | - | - |
| 1999 | Piauí | 0·5 | 4·8 | 0·7 | 10·6 | 4·9 | 6·1 | 0·425 | - | - | - | - | - |
| 1999 | Rio de Janeiro | 0·8 | 5·2 | 1·2 | 68·1 | 43·2 | 45·3 | 0·624 | - | - | - | - | - |
| 1999 | Rio Grande do Norte | 1·1 | 6·7 | 1·2 | 17·6 | 11·3 | 13·7 | 0·480 | - | - | - | - | - |
| 1999 | Rio Grande do Sul | 0·5 | 11·8 | 3·3 | 18·6 | 13·6 | 17·3 | 0·598 | - | - | - | - | - |
| 1999 | Rondônia | 1·3 | 6·6 | 1·4 | 40·8 | 27·3 | 29·9 | 0·497 | - | - | - | - | - |
| 1999 | Roraima | 1·1 | 7·3 | 1·1 | 33·3 | 12·3 | 14·4 | 0·504 | - | - | - | - | - |
| 1999 | Santa Catarina | 0·3 | 8·8 | 1·5 | 9·8 | 5·5 | 7·3 | 0·601 | - | - | - | - | - |
| 1999 | São Paulo | 0·5 | 6·1 | 1·0 | 42·4 | 29·5 | 30·9 | 0·624 | - | - | - | - | - |
| 1999 | Sergipe | 0·7 | 6·6 | 1·0 | 28·6 | 18·8 | 20·5 | 0·494 | - | - | - | - | - |
| 1999 | Tocantins | 1·0 | 5·5 | 1·2 | 18·7 | 9·2 | 11·3 | 0·466 | - | - | - | - | - |
| 2000 | Acre | 1·0 | 5·5 | 1·3 | 27·3 | 13·0 | 15·3 | 0·466 | - | - | - | - | - |
| 2000 | Alagoas | 0·3 | 3·1 | 0·4 | 34·4 | 23·7 | 24·4 | 0·431 | - | - | - | - | - |
| 2000 | Amapá | 0·6 | 5·5 | 0·7 | 38·1 | 14·0 | 15·2 | 0·546 | - | - | - | - | - |
| 2000 | Amazonas | 0·4 | 4·1 | 0·6 | 23·5 | 10·9 | 11·9 | 0·514 | - | - | - | - | - |
| 2000 | Bahia | 2·5 | 4·0 | 0·8 | 21·9 | 16·3 | 19·6 | 0·475 | - | - | - | - | - |
| 2000 | Ceará | 0·5 | 6·4 | 0·7 | 22·6 | 12·4 | 13·6 | 0·480 | - | - | - | - | - |
| 2000 | Distrito Federal | 0·2 | 4·8 | 1·1 | 33·1 | 24·2 | 25·5 | 0·696 | - | - | - | - | - |
| 2000 | Espírito Santo | 0·4 | 4·7 | 0·8 | 51·5 | 41·4 | 42·6 | 0·576 | - | - | - | - | - |
| 2000 | Goiás | 0·5 | 8·0 | 1·8 | 24·9 | 16·3 | 18·6 | 0·538 | - | - | - | - | - |
| 2000 | Maranhão | 0·7 | 3·2 | 0·5 | 18·8 | 10·0 | 11·2 | 0·383 | - | - | - | - | - |
| 2000 | Mato Grosso | 1·6 | 5·7 | 1·3 | 34·6 | 21·0 | 23·8 | 0·554 | - | - | - | - | - |
| 2000 | Mato Grosso do Sul | 0·7 | 8·2 | 2·0 | 31·4 | 20·5 | 23·2 | 0·543 | - | - | - | - | - |
| 2000 | Minas Gerais | 0·5 | 6·2 | 1·0 | 16·0 | 10·8 | 12·4 | 0·562 | - | - | - | - | - |
| 2000 | Pará | 0·9 | 3·6 | 0·6 | 23·0 | 14·3 | 15·8 | 0·473 | - | - | - | - | - |
| 2000 | Paraíba | 0·3 | 4·1 | 0·5 | 25·4 | 18·0 | 18·8 | 0·461 | - | - | - | - | - |
| 2000 | Paraná | 0·4 | 7·5 | 1·5 | 21·7 | 13·9 | 15·8 | 0·585 | - | - | - | - | - |
| 2000 | Pernambuco | 0·4 | 5·3 | 0·8 | 59·1 | 47·4 | 48·7 | 0·481 | - | - | - | - | - |
| 2000 | Piauí | 0·6 | 5·4 | 0·8 | 12·2 | 5·7 | 7·1 | 0·429 | - | - | - | - | - |
| 2000 | Rio de Janeiro | 0·8 | 4·9 | 1·2 | 66·4 | 41·8 | 43·7 | 0·628 | - | - | - | - | - |
| 2000 | Rio Grande do Norte | 1·1 | 6·8 | 1·2 | 18·2 | 12·0 | 14·2 | 0·485 | - | - | - | - | - |
| 2000 | Rio Grande do Sul | 0·4 | 11·7 | 3·2 | 19·0 | 14·0 | 17·6 | 0·603 | - | - | - | - | - |
| 2000 | Rondônia | 1·3 | 6·8 | 1·4 | 42·4 | 28·2 | 30·8 | 0·502 | - | - | - | - | - |
| 2000 | Roraima | 1·0 | 7·3 | 1·0 | 32·9 | 12·2 | 14·2 | 0·509 | - | - | - | - | - |
| 2000 | Santa Catarina | 0·3 | 8·8 | 1·5 | 10·1 | 5·8 | 7·6 | 0·606 | - | - | - | - | - |
| 2000 | São Paulo | 0·5 | 6·2 | 1·0 | 42·6 | 31·5 | 33·0 | 0·630 | - | - | - | - | - |
| 2000 | Sergipe | 0·6 | 6·4 | 0·9 | 29·6 | 19·9 | 21·4 | 0·500 | - | - | - | - | - |
| 2000 | Tocantins | 0·9 | 5·6 | 1·2 | 19·2 | 9·5 | 11·6 | 0·471 | - | - | - | - | - |
| 2001 | Acre | 1·0 | 5·7 | 1·3 | 28·0 | 13·2 | 15·5 | 0·472 | - | - | - | - | - |
| 2001 | Alagoas | 0·3 | 3·2 | 0·4 | 37·5 | 26·4 | 27·0 | 0·436 | - | - | - | - | - |
| 2001 | Amapá | 0·5 | 5·6 | 0·7 | 38·0 | 13·6 | 14·7 | 0·552 | - | - | - | - | - |
| 2001 | Amazonas | 0·4 | 4·1 | 0·5 | 22·8 | 10·5 | 11·4 | 0·519 | - | - | - | - | - |
| 2001 | Bahia | 2·3 | 4·4 | 0·8 | 23·9 | 17·9 | 21·1 | 0·480 | - | - | - | - | - |
| 2001 | Ceará | 0·4 | 6·9 | 0·8 | 23·6 | 13·1 | 14·3 | 0·486 | - | - | - | - | - |
| 2001 | Distrito Federal | 0·2 | 4·8 | 1·0 | 33·4 | 24·4 | 25·7 | 0·702 | - | - | - | - | - |
| 2001 | Espírito Santo | 0·4 | 4·7 | 0·7 | 51·9 | 41·5 | 42·6 | 0·582 | - | - | - | - | - |
| 2001 | Goiás | 0·5 | 7·6 | 1·7 | 25·3 | 16·6 | 18·7 | 0·545 | - | - | - | - | - |
| 2001 | Maranhão | 0·8 | 3·4 | 0·5 | 19·9 | 10·6 | 11·8 | 0·386 | - | - | - | - | - |
| 2001 | Mato Grosso | 1·5 | 5·9 | 1·2 | 35·4 | 21·3 | 24·0 | 0·559 | - | - | - | - | - |
| 2001 | Mato Grosso do Sul | 0·6 | 8·0 | 1·9 | 30·5 | 19·6 | 22·1 | 0·549 | - | - | - | - | - |
| 2001 | Minas Gerais | 0·4 | 6·3 | 1·0 | 17·4 | 12·1 | 13·5 | 0·567 | - | - | - | - | - |
| 2001 | Pará | 0·9 | 3·6 | 0·6 | 24·8 | 15·5 | 16·9 | 0·476 | - | - | - | - | - |
| 2001 | Paraíba | 0·3 | 4·2 | 0·5 | 25·6 | 18·1 | 18·9 | 0·465 | - | - | - | - | - |
| 2001 | Paraná | 0·3 | 7·5 | 1·5 | 23·1 | 15·1 | 16·9 | 0·591 | - | - | - | - | - |
| 2001 | Pernambuco | 0·4 | 5·2 | 0·8 | 59·7 | 48·1 | 49·2 | 0·486 | - | - | - | - | - |
| 2001 | Piauí | 0·6 | 6·1 | 0·9 | 13·4 | 6·2 | 7·7 | 0·434 | - | - | - | - | - |
| 2001 | Rio de Janeiro | 0·7 | 4·8 | 1·1 | 65·1 | 41·8 | 43·6 | 0·632 | - | - | - | - | - |
| 2001 | Rio Grande do Norte | 1·0 | 6·9 | 1·2 | 18·7 | 12·6 | 14·8 | 0·491 | - | - | - | - | - |
| 2001 | Rio Grande do Sul | 0·4 | 11·6 | 3·0 | 19·5 | 14·3 | 17·7 | 0·608 | - | - | - | - | - |
| 2001 | Rondônia | 1·2 | 6·8 | 1·3 | 44·1 | 29·5 | 32·0 | 0·508 | - | - | - | - | - |
| 2001 | Roraima | 0·9 | 7·4 | 1·0 | 32·3 | 11·9 | 13·8 | 0·514 | - | - | - | - | - |
| 2001 | Santa Catarina | 0·3 | 8·9 | 1·4 | 10·7 | 6·3 | 8·0 | 0·612 | - | - | - | - | - |
| 2001 | São Paulo | 0·5 | 6·2 | 0·9 | 41·0 | 31·3 | 32·7 | 0·636 | - | - | - | - | - |
| 2001 | Sergipe | 0·5 | 6·1 | 0·8 | 30·5 | 20·7 | 22·1 | 0·506 | - | - | - | - | - |
| 2001 | Tocantins | 0·8 | 5·7 | 1·2 | 19·5 | 9·7 | 11·7 | 0·477 | - | - | - | - | - |
| 2002 | Acre | 0·9 | 5·7 | 1·3 | 28·8 | 13·5 | 15·7 | 0·479 | - | - | - | - | - |
| 2002 | Alagoas | 0·3 | 3·2 | 0·4 | 40·3 | 28·8 | 29·4 | 0·442 | - | - | - | - | - |
| 2002 | Amapá | 0·5 | 5·5 | 0·6 | 36·9 | 13·3 | 14·4 | 0·558 | - | - | - | - | - |
| 2002 | Amazonas | 0·4 | 4·1 | 0·5 | 22·8 | 10·5 | 11·4 | 0·523 | - | - | - | - | - |
| 2002 | Bahia | 2·3 | 4·7 | 0·9 | 26·0 | 19·7 | 22·8 | 0·485 | - | - | - | - | - |
| 2002 | Ceará | 0·4 | 7·2 | 0·8 | 24·7 | 14·0 | 15·1 | 0·492 | - | - | - | - | - |
| 2002 | Distrito Federal | 0·2 | 4·7 | 1·0 | 32·7 | 24·1 | 25·2 | 0·707 | - | - | - | - | - |
| 2002 | Espírito Santo | 0·4 | 4·7 | 0·7 | 52·2 | 41·7 | 42·8 | 0·588 | - | - | - | - | - |
| 2002 | Goiás | 0·5 | 7·6 | 1·6 | 26·6 | 17·4 | 19·4 | 0·551 | - | - | - | - | - |
| 2002 | Maranhão | 0·8 | 3·5 | 0·5 | 20·7 | 10·9 | 12·2 | 0·389 | - | - | - | - | - |
| 2002 | Mato Grosso | 1·5 | 6·1 | 1·2 | 35·6 | 21·4 | 24·1 | 0·564 | - | - | - | - | - |
| 2002 | Mato Grosso do Sul | 0·6 | 8·2 | 1·8 | 30·8 | 19·5 | 21·9 | 0·555 | - | - | - | - | - |
| 2002 | Minas Gerais | 0·4 | 6·4 | 1·0 | 19·4 | 13·8 | 15·2 | 0·573 | - | - | - | - | - |
| 2002 | Pará | 0·9 | 3·6 | 0·5 | 26·4 | 16·6 | 18·0 | 0·480 | - | - | - | - | - |
| 2002 | Paraíba | 0·3 | 4·3 | 0·5 | 26·4 | 18·7 | 19·5 | 0·469 | - | - | - | - | - |
| 2002 | Paraná | 0·3 | 7·3 | 1·3 | 24·7 | 16·6 | 18·3 | 0·597 | - | - | - | - | - |
| 2002 | Pernambuco | 0·3 | 5·1 | 0·7 | 58·1 | 46·7 | 47·8 | 0·492 | - | - | - | - | - |
| 2002 | Piauí | 0·6 | 6·4 | 0·9 | 14·4 | 6·7 | 8·2 | 0·439 | - | - | - | - | - |
| 2002 | Rio de Janeiro | 0·7 | 4·7 | 1·1 | 67·9 | 42·6 | 44·4 | 0·637 | - | - | - | - | - |
| 2002 | Rio Grande do Norte | 1·0 | 6·9 | 1·2 | 19·3 | 13·2 | 15·3 | 0·497 | - | - | - | - | - |
| 2002 | Rio Grande do Sul | 0·4 | 11·6 | 2·9 | 19·9 | 14·6 | 17·9 | 0·614 | - | - | - | - | - |
| 2002 | Rondônia | 1·1 | 6·6 | 1·2 | 44·1 | 29·5 | 31·8 | 0·515 | - | - | - | - | - |
| 2002 | Roraima | 0·8 | 7·4 | 1·0 | 31·7 | 11·6 | 13·4 | 0·521 | - | - | - | - | - |
| 2002 | Santa Catarina | 0·3 | 8·9 | 1·4 | 11·4 | 6·9 | 8·5 | 0·618 | - | - | - | - | - |
| 2002 | São Paulo | 0·4 | 6·2 | 0·9 | 38·4 | 29·6 | 30·9 | 0·641 | - | - | - | - | - |
| 2002 | Sergipe | 0·5 | 6·1 | 0·8 | 30·9 | 21·1 | 22·4 | 0·512 | - | - | - | - | - |
| 2002 | Tocantins | 0·8 | 5·7 | 1·1 | 18·9 | 9·2 | 11·0 | 0·484 | - | - | - | - | - |
| 2003 | Acre | 0·8 | 5·7 | 1·2 | 28·5 | 13·1 | 15·1 | 0·485 | - | - | - | - | - |
| 2003 | Alagoas | 0·2 | 3·2 | 0·4 | 41·9 | 30·6 | 31·2 | 0·448 | - | - | - | - | - |
| 2003 | Amapá | 0·4 | 5·5 | 0·6 | 36·2 | 13·4 | 14·4 | 0·564 | - | - | - | - | - |
| 2003 | Amazonas | 0·3 | 4·1 | 0·5 | 22·6 | 10·5 | 11·3 | 0·528 | - | - | - | - | - |
| 2003 | Bahia | 2·1 | 4·8 | 0·8 | 27·8 | 21·1 | 24·0 | 0·491 | - | - | - | - | - |
| 2003 | Ceará | 0·4 | 7·4 | 0·7 | 25·6 | 14·8 | 16·0 | 0·498 | - | - | - | - | - |
| 2003 | Distrito Federal | 0·2 | 4·6 | 0·9 | 33·1 | 24·5 | 25·5 | 0·713 | - | - | - | - | - |
| 2003 | Espírito Santo | 0·3 | 4·8 | 0·7 | 51·7 | 41·0 | 42·1 | 0·593 | - | - | - | - | - |
| 2003 | Goiás | 0·4 | 7·4 | 1·4 | 27·3 | 17·9 | 19·8 | 0·558 | - | - | - | - | - |
| 2003 | Maranhão | 0·8 | 3·6 | 0·5 | 21·0 | 11·1 | 12·3 | 0·392 | - | - | - | - | - |
| 2003 | Mato Grosso | 1·5 | 6·3 | 1·2 | 35·9 | 21·4 | 24·1 | 0·570 | - | - | - | - | - |
| 2003 | Mato Grosso do Sul | 0·6 | 8·4 | 1·8 | 31·0 | 19·4 | 21·7 | 0·560 | - | - | - | - | - |
| 2003 | Minas Gerais | 0·4 | 6·8 | 1·0 | 22·0 | 16·0 | 17·3 | 0·579 | - | - | - | - | - |
| 2003 | Pará | 0·8 | 3·5 | 0·5 | 27·8 | 17·7 | 19·0 | 0·483 | - | - | - | - | - |
| 2003 | Paraíba | 0·3 | 4·4 | 0·5 | 26·5 | 18·9 | 19·6 | 0·474 | - | - | - | - | - |
| 2003 | Paraná | 0·3 | 7·4 | 1·3 | 26·7 | 18·2 | 19·9 | 0·603 | - | - | - | - | - |
| 2003 | Pernambuco | 0·3 | 5·1 | 0·7 | 56·8 | 45·4 | 46·4 | 0·497 | - | - | - | - | - |
| 2003 | Piauí | 0·5 | 6·8 | 0·9 | 15·0 | 7·2 | 8·6 | 0·444 | - | - | - | - | - |
| 2003 | Rio de Janeiro | 0·7 | 4·5 | 1·0 | 64·6 | 41·7 | 43·4 | 0·641 | - | - | - | - | - |
| 2003 | Rio Grande do Norte | 0·9 | 6·9 | 1·1 | 20·2 | 14·1 | 16·1 | 0·503 | - | - | - | - | - |
| 2003 | Rio Grande do Sul | 0·3 | 11·8 | 2·8 | 20·5 | 15·1 | 18·3 | 0·619 | - | - | - | - | - |
| 2003 | Rondônia | 1·1 | 6·6 | 1·2 | 43·9 | 29·5 | 31·7 | 0·521 | - | - | - | - | - |
| 2003 | Roraima | 0·8 | 7·7 | 0·9 | 31·6 | 11·6 | 13·3 | 0·527 | - | - | - | - | - |
| 2003 | Santa Catarina | 0·3 | 8·9 | 1·3 | 12·1 | 7·4 | 9·0 | 0·623 | - | - | - | - | - |
| 2003 | São Paulo | 0·4 | 6·1 | 0·8 | 35·1 | 26·9 | 28·2 | 0·646 | - | - | - | - | - |
| 2003 | Sergipe | 0·4 | 6·0 | 0·8 | 29·9 | 20·3 | 21·5 | 0·518 | - | - | - | - | - |
| 2003 | Tocantins | 0·7 | 5·8 | 1·1 | 18·9 | 9·1 | 10·9 | 0·491 | - | - | - | - | - |
| 2004 | Acre | 0·8 | 5·6 | 1·2 | 27·1 | 12·2 | 14·1 | 0·492 | - | - | - | - | - |
| 2004 | Alagoas | 0·2 | 3·3 | 0·4 | 43·9 | 32·6 | 33·1 | 0·455 | - | - | - | - | - |
| 2004 | Amapá | 0·4 | 5·3 | 0·5 | 34·9 | 13·1 | 14·0 | 0·570 | - | - | - | - | - |
| 2004 | Amazonas | 0·3 | 4·2 | 0·5 | 22·6 | 11·0 | 11·8 | 0·533 | - | - | - | - | - |
| 2004 | Bahia | 1·9 | 4·9 | 0·8 | 29·4 | 22·3 | 25·0 | 0·496 | - | - | - | - | - |
| 2004 | Ceará | 0·4 | 7·5 | 0·7 | 26·0 | 15·5 | 16·6 | 0·505 | - | - | - | - | - |
| 2004 | Distrito Federal | 0·1 | 4·6 | 0·9 | 32·9 | 24·2 | 25·2 | 0·719 | - | - | - | - | - |
| 2004 | Espírito Santo | 0·3 | 4·8 | 0·7 | 51·1 | 40·4 | 41·4 | 0·599 | - | - | - | - | - |
| 2004 | Goiás | 0·4 | 7·3 | 1·3 | 28·6 | 18·8 | 20·5 | 0·564 | - | - | - | - | - |
| 2004 | Maranhão | 0·7 | 3·7 | 0·5 | 20·9 | 11·0 | 12·2 | 0·396 | - | - | - | - | - |
| 2004 | Mato Grosso | 1·4 | 6·3 | 1·1 | 34·9 | 20·4 | 22·9 | 0·576 | - | - | - | - | - |
| 2004 | Mato Grosso do Sul | 0·5 | 8·6 | 1·7 | 30·7 | 18·9 | 21·2 | 0·566 | - | - | - | - | - |
| 2004 | Minas Gerais | 0·4 | 7·0 | 1·0 | 23·8 | 17·5 | 18·8 | 0·585 | - | - | - | - | - |
| 2004 | Pará | 0·8 | 3·5 | 0·5 | 29·1 | 18·7 | 20·0 | 0·488 | - | - | - | - | - |
| 2004 | Paraíba | 0·3 | 4·5 | 0·5 | 27·5 | 19·6 | 20·3 | 0·480 | - | - | - | - | - |
| 2004 | Paraná | 0·3 | 7·5 | 1·3 | 28·8 | 19·9 | 21·4 | 0·609 | - | - | - | - | - |
| 2004 | Pernambuco | 0·3 | 5·1 | 0·6 | 55·2 | 43·7 | 44·7 | 0·503 | - | - | - | - | - |
| 2004 | Piauí | 0·5 | 7·0 | 0·9 | 15·1 | 7·2 | 8·6 | 0·450 | - | - | - | - | - |
| 2004 | Rio de Janeiro | 0·6 | 4·3 | 0·9 | 61·2 | 39·6 | 41·1 | 0·645 | - | - | - | - | - |
| 2004 | Rio Grande do Norte | 0·9 | 7·0 | 1·1 | 21·0 | 14·8 | 16·8 | 0·509 | - | - | - | - | - |
| 2004 | Rio Grande do Sul | 0·3 | 11·9 | 2·7 | 20·9 | 15·4 | 18·5 | 0·624 | - | - | - | - | - |
| 2004 | Rondônia | 1·0 | 6·6 | 1·1 | 43·0 | 28·9 | 31·0 | 0·528 | - | - | - | - | - |
| 2004 | Roraima | 0·7 | 7·9 | 0·9 | 30·9 | 11·2 | 12·8 | 0·534 | - | - | - | - | - |
| 2004 | Santa Catarina | 0·2 | 8·8 | 1·2 | 12·3 | 7·6 | 9·1 | 0·629 | - | - | - | - | - |
| 2004 | São Paulo | 0·4 | 6·0 | 0·8 | 30·5 | 22·9 | 24·0 | 0·652 | - | - | - | - | - |
| 2004 | Sergipe | 0·4 | 5·9 | 0·7 | 29·3 | 19·8 | 20·9 | 0·524 | - | - | - | - | - |
| 2004 | Tocantins | 0·7 | 6·0 | 1·1 | 19·1 | 9·0 | 10·8 | 0·498 | - | - | - | - | - |
| 2005 | Acre | 0·7 | 5·6 | 1·1 | 26·2 | 11·6 | 13·4 | 0·500 | - | - | - | 0·0 | - |
| 2005 | Alagoas | 0·2 | 3·3 | 0·3 | 46·2 | 34·9 | 35·4 | 0·462 | - | - | - | 0·0 | - |
| 2005 | Amapá | 0·3 | 5·2 | 0·5 | 34·1 | 12·6 | 13·5 | 0·576 | - | - | - | 0·0 | - |
| 2005 | Amazonas | 0·3 | 4·3 | 0·5 | 23·5 | 12·0 | 12·7 | 0·539 | - | - | - | 0·0 | - |
| 2005 | Bahia | 1·7 | 5·0 | 0·8 | 31·3 | 23·7 | 26·2 | 0·503 | - | - | - | 0·0 | - |
| 2005 | Ceará | 0·4 | 7·5 | 0·7 | 26·3 | 16·2 | 17·2 | 0·512 | - | - | - | 0·0 | - |
| 2005 | Distrito Federal | 0·1 | 4·5 | 0·8 | 31·5 | 23·0 | 24·0 | 0·725 | - | - | - | 0·0 | - |
| 2005 | Espírito Santo | 0·3 | 4·9 | 0·6 | 50·8 | 39·9 | 40·8 | 0·606 | - | - | - | 0·0 | - |
| 2005 | Goiás | 0·4 | 7·1 | 1·2 | 28·6 | 18·8 | 20·4 | 0·571 | - | - | - | 2·2 | - |
| 2005 | Maranhão | 0·7 | 3·7 | 0·5 | 21·0 | 11·1 | 12·3 | 0·402 | - | - | - | 0·4 | - |
| 2005 | Mato Grosso | 1·3 | 6·3 | 1·1 | 34·3 | 20·0 | 22·4 | 0·582 | - | - | - | 0·0 | - |
| 2005 | Mato Grosso do Sul | 0·5 | 8·7 | 1·7 | 30·1 | 18·2 | 20·4 | 0·573 | - | - | - | 1·8 | - |
| 2005 | Minas Gerais | 0·3 | 7·2 | 0·9 | 24·2 | 17·8 | 19·1 | 0·591 | - | - | - | 0·0 | - |
| 2005 | Pará | 0·8 | 3·5 | 0·5 | 30·7 | 19·8 | 21·1 | 0·493 | - | - | - | 0·1 | - |
| 2005 | Paraíba | 0·3 | 4·7 | 0·5 | 28·8 | 20·8 | 21·6 | 0·486 | - | - | - | 16·9 | - |
| 2005 | Paraná | 0·3 | 7·4 | 1·2 | 29·7 | 20·8 | 22·2 | 0·615 | - | - | - | 10·7 | - |
| 2005 | Pernambuco | 0·3 | 5·1 | 0·6 | 54·1 | 42·7 | 43·6 | 0·510 | - | - | - | 0·5 | - |
| 2005 | Piauí | 0·5 | 7·0 | 0·9 | 15·3 | 7·3 | 8·7 | 0·457 | - | - | - | 0·2 | - |
| 2005 | Rio de Janeiro | 0·6 | 4·3 | 0·9 | 58·0 | 37·8 | 39·3 | 0·650 | - | - | - | 9·7 | - |
| 2005 | Rio Grande do Norte | 0·8 | 7·0 | 1·1 | 22·0 | 15·7 | 17·6 | 0·516 | - | - | - | 0·1 | - |
| 2005 | Rio Grande do Sul | 0·3 | 11·8 | 2·6 | 20·9 | 15·5 | 18·4 | 0·630 | - | - | - | 6·0 | - |
| 2005 | Rondônia | 0·9 | 6·4 | 1·1 | 41·1 | 27·7 | 29·6 | 0·535 | - | - | - | 0·0 | - |
| 2005 | Roraima | 0·7 | 8·1 | 0·9 | 30·3 | 10·5 | 12·1 | 0·543 | - | - | - | 0·0 | - |
| 2005 | Santa Catarina | 0·2 | 8·8 | 1·2 | 12·4 | 7·8 | 9·2 | 0·635 | - | - | - | 0·5 | - |
| 2005 | São Paulo | 0·3 | 5·9 | 0·7 | 26·0 | 19·0 | 20·0 | 0·657 | - | - | - | 0·3 | - |
| 2005 | Sergipe | 0·4 | 5·9 | 0·7 | 29·3 | 19·7 | 20·8 | 0·531 | - | - | - | 0·0 | - |
| 2005 | Tocantins | 0·7 | 6·2 | 1·1 | 19·5 | 9·0 | 10·7 | 0·507 | - | - | - | 0·0 | - |
| 2006 | Acre | 0·7 | 5·5 | 1·0 | 25·9 | 11·4 | 13·1 | 0·508 | - | - | - | 0·0 | - |
| 2006 | Alagoas | 0·2 | 3·5 | 0·3 | 52·6 | 40·8 | 41·4 | 0·470 | - | - | - | 0·0 | - |
| 2006 | Amapá | 0·3 | 5·0 | 0·5 | 33·6 | 12·8 | 13·5 | 0·583 | - | - | - | 0·0 | - |
| 2006 | Amazonas | 0·3 | 4·4 | 0·5 | 24·8 | 13·3 | 14·0 | 0·546 | - | - | - | 0·0 | - |
| 2006 | Bahia | 1·6 | 5·1 | 0·8 | 33·4 | 25·5 | 27·8 | 0·510 | - | - | - | 0·0 | - |
| 2006 | Ceará | 0·4 | 7·2 | 0·6 | 26·8 | 17·0 | 18·0 | 0·520 | - | - | - | 0·0 | - |
| 2006 | Distrito Federal | 0·1 | 4·4 | 0·7 | 30·7 | 22·4 | 23·3 | 0·731 | - | - | - | 946·9 | - |
| 2006 | Espírito Santo | 0·3 | 4·9 | 0·6 | 52·1 | 40·8 | 41·7 | 0·612 | - | - | - | 0·0 | - |
| 2006 | Goiás | 0·4 | 6·7 | 1·1 | 28·7 | 19·0 | 20·5 | 0·579 | - | - | - | 0·0 | - |
| 2006 | Maranhão | 0·7 | 3·7 | 0·5 | 20·9 | 11·2 | 12·4 | 0·409 | - | - | - | 0·0 | - |
| 2006 | Mato Grosso | 1·2 | 6·0 | 1·0 | 32·7 | 19·1 | 21·3 | 0·589 | - | - | - | 0·0 | - |
| 2006 | Mato Grosso do Sul | 0·5 | 8·5 | 1·6 | 29·7 | 17·8 | 19·9 | 0·580 | - | - | - | 0·0 | - |
| 2006 | Minas Gerais | 0·3 | 7·3 | 0·9 | 24·7 | 18·1 | 19·3 | 0·598 | - | - | - | 2·1 | - |
| 2006 | Pará | 0·8 | 3·5 | 0·5 | 32·3 | 21·0 | 22·2 | 0·499 | - | - | - | 0·1 | - |
| 2006 | Paraíba | 0·3 | 4·9 | 0·5 | 30·9 | 22·8 | 23·5 | 0·493 | - | - | - | 0·0 | - |
| 2006 | Paraná | 0·3 | 7·2 | 1·1 | 30·6 | 21·7 | 23·0 | 0·622 | - | - | - | 3·3 | - |
| 2006 | Pernambuco | 0·3 | 5·1 | 0·6 | 53·7 | 42·1 | 43·0 | 0·517 | - | - | - | 0·0 | - |
| 2006 | Piauí | 0·5 | 7·1 | 0·8 | 15·5 | 7·6 | 8·9 | 0·465 | - | - | - | 0·0 | - |
| 2006 | Rio de Janeiro | 0·6 | 4·3 | 0·9 | 55·7 | 37·0 | 38·5 | 0·655 | - | - | - | 1·3 | - |
| 2006 | Rio Grande do Norte | 0·8 | 6·9 | 1·0 | 23·5 | 17·1 | 18·9 | 0·524 | - | - | - | 0·0 | - |
| 2006 | Rio Grande do Sul | 0·3 | 11·7 | 2·5 | 21·3 | 15·8 | 18·6 | 0·635 | - | - | - | 2·8 | - |
| 2006 | Rondônia | 0·9 | 6·2 | 1·0 | 39·7 | 26·7 | 28·6 | 0·543 | - | - | - | 0·0 | - |
| 2006 | Roraima | 0·7 | 8·0 | 0·8 | 30·0 | 10·3 | 11·9 | 0·552 | - | - | - | 0·0 | - |
| 2006 | Santa Catarina | 0·2 | 8·7 | 1·1 | 12·8 | 8·0 | 9·3 | 0·641 | - | - | - | 0·0 | - |
| 2006 | São Paulo | 0·3 | 5·8 | 0·7 | 23·3 | 16·4 | 17·4 | 0·663 | - | - | - | 2·6 | - |
| 2006 | Sergipe | 0·3 | 5·8 | 0·7 | 30·3 | 20·6 | 21·6 | 0·538 | - | - | - | 0·0 | - |
| 2006 | Tocantins | 0·7 | 6·2 | 1·0 | 20·2 | 9·2 | 10·8 | 0·517 | - | - | - | 0·0 | - |
| 2007 | Acre | 0·6 | 5·5 | 1·0 | 25·5 | 11·4 | 13·1 | 0·517 | - | - | - | 0·0 | - |
| 2007 | Alagoas | 0·2 | 3·6 | 0·3 | 58·0 | 45·9 | 46·4 | 0·478 | - | - | - | 0·0 | - |
| 2007 | Amapá | 0·3 | 5·0 | 0·4 | 33·0 | 12·7 | 13·5 | 0·591 | - | - | - | 0·0 | - |
| 2007 | Amazonas | 0·3 | 4·7 | 0·5 | 26·6 | 14·8 | 15·6 | 0·553 | - | - | - | 0·4 | - |
| 2007 | Bahia | 1·5 | 5·1 | 0·7 | 35·0 | 26·8 | 29·1 | 0·518 | - | - | - | 0·0 | - |
| 2007 | Ceará | 0·3 | 7·0 | 0·6 | 27·5 | 18·0 | 18·9 | 0·528 | - | - | - | 0·1 | - |
| 2007 | Distrito Federal | 0·1 | 4·5 | 0·7 | 30·9 | 22·7 | 23·5 | 0·738 | - | - | - | 47·5 | - |
| 2007 | Espírito Santo | 0·3 | 5·0 | 0·6 | 53·9 | 42·2 | 43·0 | 0·618 | - | - | - | 0·4 | - |
| 2007 | Goiás | 0·4 | 6·7 | 1·1 | 30·1 | 20·1 | 21·6 | 0·586 | - | - | - | 0·0 | - |
| 2007 | Maranhão | 0·7 | 3·6 | 0·5 | 21·2 | 11·5 | 12·7 | 0·418 | - | - | - | 0·0 | - |
| 2007 | Mato Grosso | 1·1 | 5·9 | 1·0 | 31·8 | 18·6 | 20·7 | 0·596 | - | - | - | 0·0 | - |
| 2007 | Mato Grosso do Sul | 0·5 | 8·4 | 1·5 | 29·3 | 17·5 | 19·5 | 0·587 | - | - | - | 0·0 | - |
| 2007 | Minas Gerais | 0·3 | 7·4 | 0·9 | 24·7 | 18·0 | 19·3 | 0·604 | - | - | - | 0·0 | - |
| 2007 | Pará | 0·8 | 3·5 | 0·5 | 35·8 | 23·7 | 24·9 | 0·506 | - | - | - | 0·0 | - |
| 2007 | Paraíba | 0·3 | 5·1 | 0·5 | 32·5 | 24·3 | 25·0 | 0·501 | - | - | - | 0·1 | - |
| 2007 | Paraná | 0·3 | 7·1 | 1·1 | 31·6 | 22·5 | 23·9 | 0·628 | - | - | - | 1·6 | - |
| 2007 | Pernambuco | 0·3 | 5·2 | 0·6 | 53·6 | 41·8 | 42·6 | 0·525 | - | - | - | 0·0 | - |
| 2007 | Piauí | 0·5 | 7·1 | 0·8 | 15·3 | 7·6 | 8·9 | 0·473 | - | - | - | 0·0 | - |
| 2007 | Rio de Janeiro | 0·6 | 4·4 | 0·9 | 50·6 | 37·2 | 38·7 | 0·660 | - | - | - | 0·8 | - |
| 2007 | Rio Grande do Norte | 0·8 | 6·8 | 1·0 | 25·1 | 18·5 | 20·3 | 0·532 | - | - | - | 0·0 | - |
| 2007 | Rio Grande do Sul | 0·3 | 11·8 | 2·4 | 22·2 | 16·6 | 19·3 | 0·641 | - | - | - | 0·0 | - |
| 2007 | Rondônia | 0·8 | 6·0 | 0·9 | 37·3 | 25·2 | 26·9 | 0·551 | - | - | - | 0·0 | - |
| 2007 | Roraima | 0·7 | 7·9 | 0·8 | 29·4 | 9·9 | 11·4 | 0·562 | - | - | - | 0·0 | - |
| 2007 | Santa Catarina | 0·2 | 8·9 | 1·1 | 13·1 | 8·2 | 9·5 | 0·647 | - | - | - | 0·1 | - |
| 2007 | São Paulo | 0·3 | 5·8 | 0·6 | 20·7 | 14·1 | 15·0 | 0·669 | - | - | - | 0·1 | - |
| 2007 | Sergipe | 0·3 | 5·8 | 0·6 | 30·6 | 20·8 | 21·8 | 0·546 | - | - | - | 0·0 | - |
| 2007 | Tocantins | 0·6 | 6·2 | 1·0 | 20·3 | 9·1 | 10·7 | 0·527 | - | - | - | 0·0 | - |
| 2008 | Acre | 0·6 | 5·6 | 1·0 | 25·8 | 11·5 | 13·1 | 0·527 | 59·6 | 11·5 | 46·5 | 6·6 | 0·015 |
| 2008 | Alagoas | 0·2 | 3·6 | 0·3 | 59·0 | 47·2 | 47·8 | 0·487 | 12·1 | 4·2 | 20·6 | 0·0 | 0·082 |
| 2008 | Amapá | 0·3 | 5·0 | 0·4 | 33·2 | 13·1 | 13·8 | 0·598 | 17·1 | 11·7 | - | 2·3 | 0·052 |
| 2008 | Amazonas | 0·3 | 5·0 | 0·5 | 28·8 | 16·6 | 17·3 | 0·561 | 16·4 | 23·6 | 9·8 | 1·8 | 0·055 |
| 2008 | Bahia | 1·5 | 5·1 | 0·7 | 36·6 | 28·1 | 30·3 | 0·526 | 19·1 | 17·4 | 19·7 | 0·2 | 0·052 |
| 2008 | Ceará | 0·3 | 6·9 | 0·6 | 28·6 | 19·3 | 20·3 | 0·536 | 0·6 | 0·4 | - | 0·4 | 1·004 |
| 2008 | Distrito Federal | 0·1 | 4·5 | 0·7 | 31·7 | 23·3 | 24·1 | 0·744 | 39·8 | 106·1 | 49·7 | 379·1 | 0·002 |
| 2008 | Espírito Santo | 0·3 | 5·0 | 0·6 | 54·8 | 42·9 | 43·7 | 0·625 | 46·8 | 22·6 | 33·4 | 4·2 | 0·020 |
| 2008 | Goiás | 0·4 | 6·9 | 1·0 | 32·9 | 22·1 | 23·5 | 0·594 | 22·7 | 35·7 | 18·4 | 4·6 | 0·037 |
| 2008 | Maranhão | 0·7 | 3·6 | 0·4 | 21·7 | 12·0 | 13·1 | 0·427 | 4·2 | 3 | 8·5 | 0·3 | 0·223 |
| 2008 | Mato Grosso | 1·1 | 6·0 | 0·9 | 33·1 | 19·5 | 21·5 | 0·604 | 34·2 | 30·4 | 36·3 | 0·0 | 0·029 |
| 2008 | Mato Grosso do Sul | 0·5 | 8·3 | 1·4 | 29·1 | 17·3 | 19·2 | 0·594 | 44·7 | 27·5 | 33·3 | 0·2 | 0·022 |
| 2008 | Minas Gerais | 0·3 | 7·3 | 0·9 | 24·1 | 17·5 | 18·6 | 0·611 | 21·1 | 33·4 | 7·1 | 4·2 | 0·039 |
| 2008 | Pará | 0·8 | 3·6 | 0·5 | 38·6 | 25·9 | 27·1 | 0·514 | 17·9 | 14 | 15·6 | 0·8 | 0·053 |
| 2008 | Paraíba | 0·3 | 5·2 | 0·5 | 35·4 | 26·7 | 27·4 | 0·509 | 9·4 | 6·6 | 23·1 | 8·8 | 0·055 |
| 2008 | Paraná | 0·3 | 7·2 | 1·0 | 33·3 | 23·8 | 25·1 | 0·634 | 2·4 | 5·3 | 1·7 | 2·7 | 0·197 |
| 2008 | Pernambuco | 0·2 | 5·1 | 0·5 | 52·1 | 40·1 | 40·9 | 0·533 | 13·4 | 6·6 | 55·7 | 2·7 | 0·062 |
| 2008 | Piauí | 0·4 | 7·0 | 0·8 | 15·1 | 7·7 | 9·0 | 0·482 | 6 | 7·3 | 14·5 | 2·0 | 0·125 |
| 2008 | Rio de Janeiro | 0·6 | 4·5 | 0·9 | 44·1 | 36·2 | 37·6 | 0·665 | 20·4 | 23·8 | 19·1 | 2·6 | 0·044 |
| 2008 | Rio Grande do Norte | 0·8 | 6·7 | 0·9 | 26·6 | 19·8 | 21·6 | 0·541 | 13·3 | 7·6 | 19·9 | 0·2 | 0·074 |
| 2008 | Rio Grande do Sul | 0·3 | 11·7 | 2·3 | 22·8 | 17·2 | 19·8 | 0·647 | 43·8 | 65·4 | 37·8 | 9·2 | 0·019 |
| 2008 | Rondônia | 0·8 | 6·1 | 0·9 | 36·8 | 24·5 | 26·2 | 0·559 | 67·2 | 77·1 | 45·3 | 0·1 | 0·015 |
| 2008 | Roraima | 0·6 | 7·8 | 0·8 | 29·2 | 9·8 | 11·2 | 0·572 | 6·1 | 8·2 | 15 | 0·2 | 0·158 |
| 2008 | Santa Catarina | 0·2 | 9·0 | 1·1 | 13·6 | 8·6 | 9·9 | 0·653 | 34·3 | 43·4 | 20·8 | 5·8 | 0·025 |
| 2008 | São Paulo | 0·3 | 5·9 | 0·6 | 19·6 | 12·9 | 13·9 | 0·674 | 60·9 | 53·9 | 16·7 | 6·2 | 0·015 |
| 2008 | Sergipe | 0·3 | 6·0 | 0·6 | 32·0 | 21·9 | 22·9 | 0·554 | - | - | - | 5·2 | - |
| 2008 | Tocantins | 0·6 | 6·3 | 1·0 | 21·0 | 9·4 | 11·0 | 0·537 | 9·9 | 15·2 | 30·5 | 3·5 | 0·075 |
| 2009 | Acre | 0·6 | 5·7 | 1·0 | 26·2 | 11·9 | 13·4 | 0·536 | 19·5 | 43·3 | - | 4·8 | 0·041 |
| 2009 | Alagoas | 0·2 | 3·7 | 0·3 | 61·6 | 49·6 | 50·2 | 0·496 | 10·7 | 2·7 | 20 | 0·1 | 0·092 |
| 2009 | Amapá | 0·3 | 5·1 | 0·4 | 33·6 | 13·6 | 14·3 | 0·605 | 2·1 | 8·3 | 6·4 | 2·5 | 0·215 |
| 2009 | Amazonas | 0·3 | 5·0 | 0·5 | 29·7 | 17·7 | 18·4 | 0·568 | 20·9 | 34 | 19·2 | 1·7 | 0·044 |
| 2009 | Bahia | 1·5 | 5·0 | 0·7 | 37·5 | 28·9 | 31·1 | 0·534 | 23·9 | 20·2 | 17·9 | 0·1 | 0·042 |
| 2009 | Ceará | 0·3 | 6·8 | 0·6 | 30·4 | 21·3 | 22·2 | 0·544 | 0·8 | 0·8 | - | 3·3 | 0·246 |
| 2009 | Distrito Federal | 0·1 | 4·5 | 0·7 | 31·9 | 23·6 | 24·4 | 0·750 | 44·2 | 113·8 | 57·8 | 71·9 | 0·009 |
| 2009 | Espírito Santo | 0·3 | 4·9 | 0·5 | 53·9 | 42·2 | 43·0 | 0·631 | 17·3 | 11·4 | 13·7 | 3·5 | 0·048 |
| 2009 | Goiás | 0·4 | 6·8 | 1·0 | 34·5 | 23·4 | 24·7 | 0·601 | 31·9 | 35·7 | 18·6 | 8·3 | 0·025 |
| 2009 | Maranhão | 0·7 | 3·6 | 0·4 | 22·2 | 12·6 | 13·7 | 0·436 | 11·5 | 3·5 | 10·8 | 0·1 | 0·087 |
| 2009 | Mato Grosso | 1·1 | 6·1 | 0·9 | 34·3 | 20·2 | 22·2 | 0·611 | 53·4 | 49·3 | 44·9 | 0·5 | 0·019 |
| 2009 | Mato Grosso do Sul | 0·5 | 8·4 | 1·4 | 29·0 | 17·1 | 18·9 | 0·600 | 41·3 | 22·9 | 26·4 | 8·4 | 0·020 |
| 2009 | Minas Gerais | 0·3 | 7·5 | 0·9 | 24·1 | 17·3 | 18·5 | 0·618 | 54·1 | 0·7 | 8·2 | 11·1 | 0·015 |
| 2009 | Pará | 0·8 | 3·6 | 0·5 | 40·9 | 27·6 | 28·9 | 0·521 | 27·8 | 17·7 | 12·7 | 1·5 | 0·034 |
| 2009 | Paraíba | 0·3 | 5·4 | 0·5 | 39·3 | 30·2 | 31·0 | 0·517 | 8·6 | 5·6 | 20·6 | 11·6 | 0·050 |
| 2009 | Paraná | 0·3 | 7·3 | 1·0 | 34·4 | 24·6 | 25·9 | 0·640 | 2·7 | 5·9 | 1·3 | 4·9 | 0·132 |
| 2009 | Pernambuco | 0·2 | 5·0 | 0·5 | 48·8 | 37·3 | 38·0 | 0·540 | 23·7 | 12·5 | 21·6 | 2·4 | 0·038 |
| 2009 | Piauí | 0·4 | 6·9 | 0·8 | 15·2 | 7·9 | 9·1 | 0·491 | 6·7 | 7·2 | 11 | 5·4 | 0·083 |
| 2009 | Rio de Janeiro | 0·5 | 4·5 | 0·9 | 40·5 | 34·6 | 36·0 | 0·670 | 44·4 | 24·6 | 19·9 | 3·8 | 0·021 |
| 2009 | Rio Grande do Norte | 0·8 | 6·7 | 0·9 | 28·7 | 21·7 | 23·4 | 0·549 | 15·6 | 6·5 | 18·2 | 7·7 | 0·043 |
| 2009 | Rio Grande do Sul | 0·3 | 11·7 | 2·3 | 23·4 | 17·6 | 20·2 | 0·653 | 57·2 | 75·2 | 23·6 | 23·9 | 0·012 |
| 2009 | Rondônia | 0·8 | 6·3 | 0·9 | 37·9 | 25·3 | 27·0 | 0·567 | 74·5 | 80·1 | 39·8 | 2·3 | 0·013 |
| 2009 | Roraima | 0·6 | 7·6 | 0·7 | 28·9 | 9·7 | 11·0 | 0·581 | 5·5 | 26·1 | 102·3 | 10·6 | 0·062 |
| 2009 | Santa Catarina | 0·2 | 9·2 | 1·0 | 14·1 | 8·9 | 10·2 | 0·659 | 18·8 | 21·9 | 14·5 | 5·4 | 0·041 |
| 2009 | São Paulo | 0·3 | 6·1 | 0·6 | 19·1 | 12·3 | 13·2 | 0·680 | 67·4 | 56·2 | 17·7 | 3·4 | 0·014 |
| 2009 | Sergipe | 0·3 | 6·2 | 0·6 | 34·5 | 23·9 | 24·9 | 0·562 | 0·6 | 3·4 | 1·3 | 4·8 | 0·185 |
| 2009 | Tocantins | 0·6 | 6·4 | 1·0 | 22·3 | 10·1 | 11·7 | 0·547 | 19·5 | 14·2 | 23·3 | 5·2 | 0·040 |
| 2010 | Acre | 0·6 | 5·7 | 0·9 | 26·9 | 12·3 | 13·8 | 0·546 | 19·4 | 323·2 | 4·8 | 5·4 | 0·040 |
| 2010 | Alagoas | 0·2 | 3·8 | 0·3 | 65·6 | 53·2 | 53·7 | 0·505 | 15·4 | 4·2 | 26 | 2·7 | 0·055 |
| 2010 | Amapá | 0·3 | 5·1 | 0·4 | 34·5 | 14·3 | 15·0 | 0·613 | 3·3 | 2·5 | 3·4 | 1·0 | 0·231 |
| 2010 | Amazonas | 0·3 | 5·2 | 0·5 | 31·7 | 19·3 | 20·0 | 0·577 | 29·1 | 47·8 | 26·3 | 4·5 | 0·030 |
| 2010 | Bahia | 1·4 | 4·9 | 0·7 | 37·6 | 28·9 | 30·9 | 0·542 | 27·5 | 21·8 | 16·9 | 0·2 | 0·036 |
| 2010 | Ceará | 0·3 | 6·9 | 0·5 | 33·5 | 24·2 | 25·1 | 0·553 | 16·8 | 15·6 | - | 4·2 | 0·048 |
| 2010 | Distrito Federal | 0·1 | 4·5 | 0·6 | 31·7 | 23·4 | 24·1 | 0·756 | 60·7 | 134·1 | 53·5 | 21·2 | 0·012 |
| 2010 | Espírito Santo | 0·3 | 4·9 | 0·5 | 51·7 | 40·3 | 41·1 | 0·638 | 57·8 | 34·2 | 38·4 | 1·3 | 0·017 |
| 2010 | Goiás | 0·3 | 6·8 | 0·9 | 36·6 | 25·0 | 26·3 | 0·608 | 27·3 | 32·9 | 15 | 2·3 | 0·034 |
| 2010 | Maranhão | 0·6 | 3·6 | 0·4 | 22·7 | 13·0 | 14·1 | 0·446 | 6·9 | 2·7 | 5·7 | 0·0 | 0·144 |
| 2010 | Mato Grosso | 1·0 | 6·1 | 0·9 | 34·8 | 20·6 | 22·6 | 0·618 | 53·5 | 44·1 | 47·3 | 5·3 | 0·017 |
| 2010 | Mato Grosso do Sul | 0·4 | 8·5 | 1·3 | 29·0 | 16·9 | 18·7 | 0·607 | 57·5 | 37·6 | 29·7 | 5·8 | 0·016 |
| 2010 | Minas Gerais | 0·3 | 7·6 | 0·8 | 24·6 | 17·6 | 18·8 | 0·624 | 89 | 100·8 | 8·3 | 7·5 | 0·010 |
| 2010 | Pará | 0·8 | 3·6 | 0·5 | 43·9 | 30·0 | 31·2 | 0·529 | 31·7 | 18·7 | 28·7 | 1·6 | 0·030 |
| 2010 | Paraíba | 0·3 | 5·5 | 0·5 | 42·1 | 32·9 | 33·6 | 0·526 | 7·9 | 5 | 17·4 | 3·1 | 0·091 |
| 2010 | Paraná | 0·3 | 7·2 | 1·0 | 34·5 | 24·6 | 25·8 | 0·646 | 45·4 | 50·6 | 37·3 | 4·2 | 0·020 |
| 2010 | Pernambuco | 0·2 | 4·8 | 0·5 | 45·6 | 34·4 | 35·1 | 0·548 | 36·8 | 16·8 | 21·2 | 7·2 | 0·023 |
| 2010 | Piauí | 0·4 | 6·9 | 0·7 | 15·5 | 8·3 | 9·5 | 0·500 | 8·8 | 4·3 | 7·6 | 0·7 | 0·105 |
| 2010 | Rio de Janeiro | 0·5 | 4·3 | 0·8 | 38·0 | 31·5 | 32·8 | 0·675 | 29·7 | 29·5 | 21·1 | 3·7 | 0·030 |
| 2010 | Rio Grande do Norte | 0·7 | 6·5 | 0·9 | 31·2 | 23·9 | 25·5 | 0·558 | 18·7 | 7·2 | 18·6 | 4·3 | 0·044 |
| 2010 | Rio Grande do Sul | 0·3 | 11·4 | 2·2 | 23·2 | 17·4 | 19·8 | 0·659 | 68·1 | 93·5 | 23·9 | 17·7 | 0·012 |
| 2010 | Rondônia | 0·8 | 6·4 | 0·9 | 38·3 | 25·6 | 27·2 | 0·575 | 89·9 | 85·2 | 40·6 | 2·4 | 0·011 |
| 2010 | Roraima | 0·6 | 7·6 | 0·7 | 29·3 | 9·9 | 11·1 | 0·591 | 7·1 | 18·4 | 85·5 | 1·5 | 0·116 |
| 2010 | Santa Catarina | 0·2 | 9·2 | 1·0 | 14·0 | 8·8 | 10·0 | 0·665 | 51·5 | 57·4 | 59·6 | 5·0 | 0·018 |
| 2010 | São Paulo | 0·3 | 6·2 | 0·6 | 18·3 | 11·5 | 12·4 | 0·685 | 73·7 | 18·8 | 5 | 6·8 | 0·012 |
| 2010 | Sergipe | 0·3 | 6·3 | 0·6 | 36·6 | 25·7 | 26·7 | 0·570 | 1·2 | 3·2 | 5·2 | 10·4 | 0·086 |
| 2010 | Tocantins | 0·6 | 6·6 | 1·0 | 23·6 | 10·8 | 12·4 | 0·558 | 27·3 | 16 | 18·9 | 1·9 | 0·034 |
| 2011 | Acre | 0·6 | 5·8 | 0·9 | 27·6 | 12·7 | 14·2 | 0·556 | 45·3 | 13·9 | 11·9 | 23·9 | 0·014 |
| 2011 | Alagoas | 0·2 | 3·8 | 0·3 | 67·1 | 54·9 | 55·4 | 0·514 | 20·5 | 5·4 | 26·4 | 8·0 | 0·035 |
| 2011 | Amapá | 0·3 | 5·3 | 0·4 | 35·3 | 15·0 | 15·7 | 0·621 | 9·1 | 1·3 | 1·9 | 5·1 | 0·070 |
| 2011 | Amazonas | 0·3 | 5·6 | 0·5 | 34·9 | 21·7 | 22·5 | 0·585 | 37·4 | 45·6 | 26 | 7·5 | 0·022 |
| 2011 | Bahia | 1·3 | 4·9 | 0·6 | 36·8 | 28·2 | 30·1 | 0·551 | 33 | 29·7 | 16·8 | 15·1 | 0·021 |
| 2011 | Ceará | 0·3 | 7·1 | 0·5 | 36·9 | 27·3 | 28·2 | 0·561 | 43·7 | - | - | 8·0 | 0·019 |
| 2011 | Distrito Federal | 0·1 | 4·6 | 0·6 | 32·1 | 23·7 | 24·4 | 0·763 | 79·4 | 160·3 | 49·7 | 20·6 | 0·010 |
| 2011 | Espírito Santo | 0·2 | 4·9 | 0·5 | 49·7 | 38·8 | 39·5 | 0·644 | 65·2 | 50·2 | 38·3 | 9·6 | 0·013 |
| 2011 | Goiás | 0·3 | 7·1 | 0·9 | 39·5 | 27·4 | 28·7 | 0·616 | 30·9 | 39·8 | 16·9 | 9·7 | 0·025 |
| 2011 | Maranhão | 0·6 | 3·7 | 0·4 | 23·3 | 13·8 | 14·8 | 0·456 | 7·9 | 3·6 | 6·9 | 2·8 | 0·093 |
| 2011 | Mato Grosso | 1·0 | 6·1 | 0·9 | 35·7 | 21·4 | 23·4 | 0·626 | 41·7 | 42·4 | 40·7 | 15·3 | 0·018 |
| 2011 | Mato Grosso do Sul | 0·4 | 8·6 | 1·3 | 28·8 | 16·6 | 18·3 | 0·614 | 64·3 | 49·6 | 27·4 | 18·4 | 0·012 |
| 2011 | Minas Gerais | 0·3 | 7·9 | 0·9 | 25·6 | 18·4 | 19·6 | 0·631 | 105·1 | 109·6 | 19·2 | 16·5 | 0·008 |
| 2011 | Pará | 0·7 | 3·6 | 0·5 | 43·2 | 29·4 | 30·6 | 0·538 | 7·7 | 22·4 | 6·4 | 5·6 | 0·075 |
| 2011 | Paraíba | 0·3 | 5·5 | 0·4 | 43·4 | 34·3 | 35·0 | 0·535 | 15·1 | 7·2 | 24·4 | 11·6 | 0·037 |
| 2011 | Paraná | 0·2 | 7·3 | 0·9 | 34·1 | 24·2 | 25·4 | 0·652 | 51·8 | 65·1 | 56·7 | 15·6 | 0·015 |
| 2011 | Pernambuco | 0·2 | 4·9 | 0·5 | 44·5 | 33·3 | 34·0 | 0·556 | 46·9 | 28 | 25·4 | 24·7 | 0·014 |
| 2011 | Piauí | 0·4 | 7·2 | 0·8 | 16·5 | 9·1 | 10·3 | 0·510 | 12·5 | 8·1 | 8·2 | 5·8 | 0·055 |
| 2011 | Rio de Janeiro | 0·4 | 4·2 | 0·8 | 34·4 | 29·3 | 30·5 | 0·681 | 28·7 | 38·5 | 22·8 | 34·0 | 0·016 |
| 2011 | Rio Grande do Norte | 0·8 | 6·9 | 0·9 | 35·8 | 27·9 | 29·5 | 0·567 | 7·4 | 4·1 | 5·5 | 9·6 | 0·059 |
| 2011 | Rio Grande do Sul | 0·3 | 11·4 | 2·1 | 23·2 | 17·5 | 19·8 | 0·665 | 79·7 | 103 | 22·5 | 43·2 | 0·008 |
| 2011 | Rondônia | 0·7 | 6·4 | 0·8 | 37·5 | 25·0 | 26·5 | 0·584 | 84·7 | 97·6 | 40·4 | 5·5 | 0·011 |
| 2011 | Roraima | 0·6 | 7·7 | 0·7 | 29·5 | 10·1 | 11·3 | 0·601 | 17·2 | 20 | 53·9 | 7·8 | 0·040 |
| 2011 | Santa Catarina | 0·2 | 9·2 | 1·0 | 14·0 | 8·8 | 9·9 | 0·672 | - | 68 | 49·1 | 15·4 | - |
| 2011 | São Paulo | 0·3 | 6·3 | 0·6 | 18·0 | 11·0 | 11·9 | 0·691 | 85·6 | 62·8 | 15·9 | 25·0 | 0·009 |
| 2011 | Sergipe | 0·3 | 6·4 | 0·6 | 39·0 | 28·0 | 28·9 | 0·578 | 0·7 | 3·2 | 2·4 | 14·1 | 0·067 |
| 2011 | Tocantins | 0·6 | 6·8 | 1·0 | 24·7 | 11·5 | 13·0 | 0·568 | 29·3 | 22·3 | 26·4 | 5·1 | 0·029 |
| 2012 | Acre | 0·6 | 5·9 | 0·9 | 28·8 | 13·7 | 15·2 | 0·565 | 244·6 | - | 72·1 | 30·8 | 0·004 |
| 2012 | Alagoas | 0·2 | 3·8 | 0·3 | 65·7 | 53·9 | 54·4 | 0·523 | 19·2 | 4·2 | 52·5 | 8·1 | 0·037 |
| 2012 | Amapá | 0·3 | 5·5 | 0·4 | 36·8 | 16·3 | 17·0 | 0·629 | 20·2 | 14·3 | 12·7 | 3·5 | 0·042 |
| 2012 | Amazonas | 0·3 | 6·0 | 0·5 | 37·4 | 23·5 | 24·2 | 0·594 | 60·6 | 53·7 | 22·3 | 3·0 | 0·016 |
| 2012 | Bahia | 1·3 | 4·9 | 0·6 | 36·6 | 28·1 | 30·0 | 0·559 | 30·7 | 24·4 | 13·9 | 30·6 | 0·016 |
| 2012 | Ceará | 0·3 | 7·4 | 0·6 | 42·7 | 32·6 | 33·4 | 0·569 | 33 | 2·6 | 28·9 | 3·5 | 0·027 |
| 2012 | Distrito Federal | 0·1 | 4·6 | 0·6 | 32·1 | 23·7 | 24·4 | 0·769 | 73 | 159·1 | 56·6 | 12·6 | 0·012 |
| 2012 | Espírito Santo | 0·2 | 4·9 | 0·5 | 48·6 | 37·9 | 38·7 | 0·651 | 111·1 | 89·6 | 47·4 | 7·2 | 0·008 |
| 2012 | Goiás | 0·3 | 7·4 | 1·0 | 43·2 | 30·3 | 31·6 | 0·623 | 44·4 | 62·2 | 23·8 | 5·1 | 0·020 |
| 2012 | Maranhão | 0·6 | 3·7 | 0·4 | 23·7 | 14·4 | 15·4 | 0·467 | 12·1 | 4·3 | 9·8 | 3·6 | 0·064 |
| 2012 | Mato Grosso | 1·1 | 6·3 | 0·9 | 36·9 | 22·3 | 24·3 | 0·633 | 75 | 120·3 | 33 | 5·6 | 0·012 |
| 2012 | Mato Grosso do Sul | 0·4 | 8·6 | 1·3 | 28·2 | 16·1 | 17·8 | 0·622 | 100·3 | 94·8 | 47·1 | 12·6 | 0·009 |
| 2012 | Minas Gerais | 0·3 | 8·0 | 0·8 | 26·2 | 18·9 | 20·1 | 0·637 | 122·2 | 114·7 | 24·9 | 13·3 | 0·007 |
| 2012 | Pará | 0·7 | 3·7 | 0·5 | 42·8 | 29·1 | 30·3 | 0·546 | 55·9 | 25·3 | 18·5 | 1·9 | 0·017 |
| 2012 | Paraíba | 0·3 | 5·6 | 0·4 | 43·6 | 34·5 | 35·2 | 0·543 | 14·3 | 6·2 | 24·2 | 7·8 | 0·045 |
| 2012 | Paraná | 0·2 | 7·4 | 0·9 | 33·6 | 23·8 | 25·0 | 0·658 | 58 | 68 | 51·9 | 8·9 | 0·015 |
| 2012 | Pernambuco | 0·2 | 4·9 | 0·5 | 42·9 | 32·0 | 32·7 | 0·564 | 48·8 | 26·1 | 24·4 | 9·6 | 0·017 |
| 2012 | Piauí | 0·4 | 7·5 | 0·8 | 17·9 | 10·2 | 11·4 | 0·518 | 14·9 | 8·6 | 17·8 | 4·7 | 0·051 |
| 2012 | Rio de Janeiro | 0·4 | 4·2 | 0·7 | 33·7 | 28·7 | 29·8 | 0·686 | 38·4 | 49·2 | 23 | 13·6 | 0·019 |
| 2012 | Rio Grande do Norte | 0·8 | 6·9 | 0·9 | 38·4 | 30·2 | 31·9 | 0·575 | 10·4 | 5·6 | 11·2 | 5·3 | 0·063 |
| 2012 | Rio Grande do Sul | 0·3 | 11·6 | 2·1 | 24·2 | 18·3 | 20·7 | 0·670 | 86·4 | 122·5 | 22·2 | 28·8 | 0·009 |
| 2012 | Rondônia | 0·7 | 6·4 | 0·8 | 37·2 | 24·9 | 26·4 | 0·592 | 74·1 | 87·7 | 44·9 | 3·7 | 0·013 |
| 2012 | Roraima | 0·6 | 7·8 | 0·7 | 30·4 | 10·4 | 11·7 | 0·611 | 21·5 | 14·7 | 7 | 1·2 | 0·044 |
| 2012 | Santa Catarina | 0·2 | 9·1 | 0·9 | 13·8 | 8·7 | 9·8 | 0·678 | 66·9 | 69·7 | 64·2 | 11·4 | 0·013 |
| 2012 | São Paulo | 0·3 | 6·4 | 0·6 | 17·8 | 10·9 | 11·7 | 0·697 | 67·8 | 67·8 | 16·6 | 17·9 | 0·012 |
| 2012 | Sergipe | 0·3 | 6·5 | 0·6 | 42·1 | 30·9 | 31·9 | 0·586 | 7·9 | 5·8 | 11·8 | 7·5 | 0·065 |
| 2012 | Tocantins | 0·6 | 6·9 | 1·0 | 25·6 | 12·1 | 13·7 | 0·577 | 34·4 | 27·9 | 24·2 | 3·5 | 0·026 |
| 2013 | Acre | 0·6 | 6·0 | 0·9 | 29·5 | 14·5 | 16·0 | 0·575 | 38·6 | 43·4 | 69·8 | 19·5 | 0·017 |
| 2013 | Alagoas | 0·2 | 3·9 | 0·3 | 65·6 | 54·0 | 54·5 | 0·531 | 29·1 | 4·4 | 23·4 | 18·7 | 0·021 |
| 2013 | Amapá | 0·3 | 5·8 | 0·4 | 38·0 | 17·5 | 18·2 | 0·636 | 25·0 | 26·7 | 40·0 | 2·6 | 0·036 |
| 2013 | Amazonas | 0·3 | 6·1 | 0·5 | 37·1 | 23·3 | 24·1 | 0·602 | 24·6 | 47·1 | 83·3 | 2·9 | 0·036 |
| 2013 | Bahia | 1·2 | 4·9 | 0·6 | 36·0 | 27·7 | 29·6 | 0·567 | - | 33·9 | - | 24·8 | - |
| 2013 | Ceará | 0·3 | 7·7 | 0·6 | 46·9 | 36·4 | 37·3 | 0·577 | 30·7 | 16·9 | 37·8 | 5·0 | 0·028 |
| 2013 | Distrito Federal | 0·1 | 4·4 | 0·6 | 30·2 | 22·3 | 23·0 | 0·775 | 52·1 | 174·0 | 75·0 | 15·4 | 0·015 |
| 2013 | Espírito Santo | 0·2 | 4·8 | 0·5 | 47·1 | 36·7 | 37·5 | 0·657 | 43·5 | 109·0 | 144·6 | 11·6 | 0·018 |
| 2013 | Goiás | 0·3 | 7·6 | 1·0 | 44·9 | 31·6 | 32·9 | 0·630 | 36·5 | 68·7 | 50·1 | 3·6 | 0·025 |
| 2013 | Maranhão | 0·6 | 3·7 | 0·4 | 24·4 | 15·0 | 16·0 | 0·477 | 13·9 | 7·6 | 15·4 | 3·5 | 0·058 |
| 2013 | Mato Grosso | 1·0 | 6·3 | 0·9 | 37·8 | 23·2 | 25·1 | 0·641 | 36·8 | 92·5 | 89·1 | 5·7 | 0·024 |
| 2013 | Mato Grosso do Sul | 0·4 | 8·6 | 1·3 | 27·5 | 15·6 | 17·3 | 0·629 | 61·7 | 96·4 | 110·9 | 12·9 | 0·013 |
| 2013 | Minas Gerais | 0·3 | 8·0 | 0·8 | 26·3 | 19·1 | 20·2 | 0·643 | 34·7 | 124·9 | 156·7 | 16·1 | 0·020 |
| 2013 | Pará | 0·7 | 3·6 | 0·4 | 41·9 | 28·4 | 29·6 | 0·554 | 27·4 | 23·7 | 54·0 | 2·7 | 0·033 |
| 2013 | Paraíba | 0·2 | 5·6 | 0·4 | 42·8 | 34·0 | 34·7 | 0·551 | - | - | - | 8·1 | - |
| 2013 | Paraná | 0·2 | 7·3 | 0·9 | 31·3 | 21·9 | 23·1 | 0·664 | 48·8 | 80·6 | 69·4 | 12·6 | 0·016 |
| 2013 | Pernambuco | 0·2 | 4·8 | 0·4 | 41·3 | 30·8 | 31·4 | 0·572 | 22·5 | 37·0 | 54·9 | 32·8 | 0·018 |
| 2013 | Piauí | 0·4 | 7·8 | 0·8 | 19·2 | 11·2 | 12·4 | 0·527 | 17·2 | 7·9 | 16·1 | 4·0 | 0·047 |
| 2013 | Rio de Janeiro | 0·4 | 4·3 | 0·7 | 34·5 | 29·3 | 30·5 | 0·692 | 29·8 | 57·6 | 107·6 | 11·4 | 0·024 |
| 2013 | Rio Grande do Norte | 0·8 | 6·9 | 0·9 | 41·7 | 33·3 | 34·9 | 0·583 | 6·2 | 2·1 | 8·6 | 4·6 | 0·093 |
| 2013 | Rio Grande do Sul | 0·3 | 11·7 | 2·1 | 24·9 | 18·9 | 21·3 | 0·676 | 20·1 | 138·0 | 90·3 | 32·0 | 0·019 |
| 2013 | Rondônia | 0·7 | 6·5 | 0·8 | 36·7 | 24·6 | 26·1 | 0·599 | 42·2 | 77·8 | 65·0 | 9·3 | 0·019 |
| 2013 | Roraima | 0·6 | 7·8 | 0·7 | 31·0 | 11·0 | 12·2 | 0·620 | - | 13·5 | 8·2 | 3·0 | - |
| 2013 | Santa Catarina | 0·2 | 9·0 | 0·9 | 13·3 | 8·4 | 9·5 | 0·684 | 52·1 | 72·4 | 93·2 | 14·5 | 0·015 |
| 2013 | São Paulo | 0·3 | 6·4 | 0·6 | 17·4 | 10·5 | 11·4 | 0·703 | 15·7 | 69·7 | 99·8 | 19·2 | 0·029 |
| 2013 | Sergipe | 0·3 | 6·7 | 0·6 | 45·4 | 34·0 | 35·0 | 0·594 | 26·3 | 11·2 | 24·6 | 7·0 | 0·030 |
| 2013 | Tocantins | 0·6 | 7·0 | 1·0 | 26·2 | 12·6 | 14·1 | 0·586 | 22·9 | 34·2 | 43·5 | 3·6 | 0·038 |
| 2014 | Acre | 0·6 | 6·1 | 0·9 | 30·1 | 15·3 | 16·8 | 0·583 | - | - | - | 4·3 | - |
| 2014 | Alagoas | 0·2 | 3·8 | 0·3 | 62·7 | 51·7 | 52·1 | 0·539 | 35·3 | 7·9 | 35·3 | 2·6 | 0·026 |
| 2014 | Amapá | 0·3 | 6·1 | 0·4 | 39·4 | 18·8 | 19·5 | 0·643 | 39·0 | 49·9 | 39·0 | 2·4 | 0·024 |
| 2014 | Amazonas | 0·3 | 6·3 | 0·5 | 38·4 | 24·1 | 24·9 | 0·611 | 71·9 | 34·8 | 71·9 | 1·6 | 0·014 |
| 2014 | Bahia | 1·2 | 5·0 | 0·6 | 35·9 | 27·7 | 29·6 | 0·575 | 33·8 | 25·4 | 33·8 | 2·1 | 0·028 |
| 2014 | Ceará | 0·3 | 7·7 | 0·6 | 48·0 | 37·7 | 38·6 | 0·584 | 45·6 | 33·8 | 45·6 | 1·7 | 0·021 |
| 2014 | Distrito Federal | 0·1 | 4·4 | 0·6 | 29·1 | 21·4 | 22·1 | 0·780 | 81·7 | 185·7 | 81·7 | 11·2 | 0·011 |
| 2014 | Espírito Santo | 0·2 | 4·8 | 0·5 | 44·9 | 35·0 | 35·7 | 0·663 | 150·6 | 123·1 | 150·6 | 11·9 | 0·006 |
| 2014 | Goiás | 0·3 | 7·6 | 0·9 | 45·3 | 32·1 | 33·4 | 0·636 | 106·7 | 127·3 | 106·7 | 2·1 | 0·009 |
| 2014 | Maranhão | 0·6 | 3·7 | 0·4 | 25·0 | 15·6 | 16·6 | 0·486 | 20·7 | 12·0 | 20·7 | 2·5 | 0·043 |
| 2014 | Mato Grosso | 1·0 | 6·2 | 0·9 | 38·3 | 23·6 | 25·5 | 0·648 | 90·1 | 89·1 | 90·1 | 4·6 | 0·011 |
| 2014 | Mato Grosso do Sul | 0·4 | 8·4 | 1·3 | 26·9 | 15·3 | 17·0 | 0·636 | 114·8 | 100·9 | 114·8 | 5·2 | 0·008 |
| 2014 | Minas Gerais | 0·3 | 8·0 | 0·8 | 26·0 | 18·9 | 20·1 | 0·649 | 126·8 | 88·9 | 126·8 | 8·2 | 0·007 |
| 2014 | Pará | 0·7 | 3·6 | 0·4 | 41·7 | 28·3 | 29·5 | 0·562 | 56·9 | 27·6 | 56·9 | 2·4 | 0·017 |
| 2014 | Paraíba | 0·2 | 5·4 | 0·4 | 41·3 | 32·8 | 33·4 | 0·559 | 14·9 | 21·3 | 14·9 | 4·4 | 0·052 |
| 2014 | Paraná | 0·2 | 7·1 | 0·9 | 29·7 | 20·7 | 21·9 | 0·670 | 81·0 | 99·4 | 81·0 | 7·4 | 0·011 |
| 2014 | Pernambuco | 0·2 | 4·8 | 0·4 | 41·6 | 31·1 | 31·8 | 0·579 | 57·4 | 39·0 | 57·4 | 17·8 | 0·013 |
| 2014 | Piauí | 0·4 | 8·0 | 0·8 | 20·3 | 12·1 | 13·3 | 0·535 | 15·3 | 10·3 | 15·3 | 1·8 | 0·058 |
| 2014 | Rio de Janeiro | 0·4 | 4·3 | 0·7 | 34·2 | 29·0 | 30·2 | 0·697 | 74·6 | 71·4 | 74·6 | 7·4 | 0·012 |
| 2014 | Rio Grande do Norte | 0·8 | 6·9 | 0·9 | 43·9 | 35·3 | 36·9 | 0·590 | 10·7 | 3·5 | 10·7 | 3·0 | 0·073 |
| 2014 | Rio Grande do Sul | 0·3 | 11·6 | 2·1 | 26·0 | 19·9 | 22·3 | 0·681 | 88·4 | 120·7 | 88·4 | 14·2 | 0·010 |
| 2014 | Rondônia | 0·7 | 6·6 | 0·8 | 37·9 | 25·6 | 27·2 | 0·606 | 59·0 | 74·9 | 59·0 | 3·8 | 0·016 |
| 2014 | Roraima | 0·6 | 7·8 | 0·6 | 30·8 | 11·1 | 12·3 | 0·628 | 21·1 | 16·5 | 21·1 | 4·5 | 0·039 |
| 2014 | Santa Catarina | 0·2 | 9·1 | 0·9 | 13·7 | 8·6 | 9·7 | 0·690 | 84·2 | 72·2 | 84·2 | 7·2 | 0·011 |
| 2014 | São Paulo | 0·3 | 6·4 | 0·6 | 17·1 | 10·3 | 11·2 | 0·708 | 94·4 | - | 94·4 | 13·6 | 0·009 |
| 2014 | Sergipe | 0·3 | 6·8 | 0·6 | 48·8 | 37·4 | 38·3 | 0·601 | 24·6 | 10·8 | 24·6 | 2·8 | 0·037 |
| 2014 | Tocantins | 0·6 | 7·1 | 1·0 | 27·9 | 13·7 | 15·3 | 0·594 | 44·6 | 31·5 | 44·6 | 2·5 | 0·021 |
| 2015 | Acre | 0·6 | 6·2 | 0·9 | 31·2 | 16·4 | 17·9 | 0·591 | - | - | - | 7·6 | - |
| 2015 | Alagoas | 0·2 | 3·7 | 0·3 | 58·3 | 47·8 | 48·3 | 0·546 | 26·6 | 12·2 | 37·4 | 5·7 | 0·031 |
| 2015 | Amapá | 0·3 | 6·3 | 0·4 | 41·2 | 19·9 | 20·7 | 0·650 | 23·6 | 37·4 | 37·2 | 1·5 | 0·040 |
| 2015 | Amazonas | 0·3 | 6·6 | 0·5 | 40·3 | 25·4 | 26·2 | 0·618 | 18·0 | 25·3 | 49·1 | 1·6 | 0·051 |
| 2015 | Bahia | 1·3 | 5·2 | 0·7 | 36·2 | 28·0 | 29·9 | 0·582 | 18·0 | 33·2 | 36·5 | 1·9 | 0·050 |
| 2015 | Ceará | 0·3 | 7·6 | 0·6 | 45·3 | 35·5 | 36·4 | 0·591 | 41·5 | 33·5 | 52·1 | 2·0 | 0·023 |
| 2015 | Distrito Federal | 0·1 | 4·4 | 0·6 | 27·9 | 20·3 | 21·0 | 0·785 | 52·9 | 250·5 | 99·9 | 12·0 | 0·015 |
| 2015 | Espírito Santo | 0·2 | 4·7 | 0·5 | 41·5 | 32·0 | 32·6 | 0·669 | 55·5 | 86·4 | 152·5 | 6·7 | 0·016 |
| 2015 | Goiás | 0·4 | 7·6 | 0·9 | 45·3 | 32·2 | 33·5 | 0·642 | 82·2 | 123·4 | 101·7 | 2·3 | 0·012 |
| 2015 | Maranhão | 0·6 | 3·7 | 0·4 | 24·8 | 15·7 | 16·7 | 0·495 | 23·3 | 16·2 | 27·4 | 2·0 | 0·039 |
| 2015 | Mato Grosso | 1·0 | 6·1 | 0·8 | 37·2 | 23·0 | 24·8 | 0·654 | 58·0 | 120·0 | 81·6 | 5·0 | 0·016 |
| 2015 | Mato Grosso do Sul | 0·4 | 8·4 | 1·3 | 25·9 | 14·6 | 16·2 | 0·642 | 42·0 | 118·7 | 124·9 | 5·7 | 0·021 |
| 2015 | Minas Gerais | 0·3 | 8·0 | 0·8 | 25·4 | 18·4 | 19·5 | 0·654 | 25·4 | 92·7 | 127·9 | 5·7 | 0·032 |
| 2015 | Pará | 0·7 | 3·8 | 0·4 | 42·4 | 28·9 | 30·1 | 0·569 | 27·5 | 31·3 | 59·9 | 2·3 | 0·034 |
| 2015 | Paraíba | 0·2 | 5·4 | 0·4 | 39·7 | 31·5 | 32·2 | 0·565 | 25·7 | 10·0 | 13·7 | 3·5 | 0·034 |
| 2015 | Paraná | 0·2 | 7·3 | 0·9 | 29·1 | 20·2 | 21·4 | 0·675 | 47·5 | 106·1 | 79·4 | 7·3 | 0·018 |
| 2015 | Pernambuco | 0·2 | 5·0 | 0·5 | 44·2 | 33·4 | 34·0 | 0·585 | 23·7 | 61·6 | 51·0 | 11·7 | 0·028 |
| 2015 | Piauí | 0·4 | 8·1 | 0·8 | 20·2 | 12·0 | 13·3 | 0·542 | 17·5 | 12·4 | 22·0 | 1·3 | 0·053 |
| 2015 | Rio de Janeiro | 0·5 | 4·3 | 0·7 | 33·2 | 28·1 | 29·3 | 0·702 | 23·0 | 71·2 | 83·2 | 7·4 | 0·033 |
| 2015 | Rio Grande do Norte | 0·8 | 6·9 | 0·9 | 44·8 | 36·1 | 37·8 | 0·597 | 5·8 | 6·3 | 8·1 | 2·0 | 0·129 |
| 2015 | Rio Grande do Sul | 0·3 | 11·6 | 2·1 | 26·4 | 20·4 | 22·8 | 0·686 | 47·7 | 102·6 | 81·3 | 12·9 | 0·017 |
| 2015 | Rondônia | 0·7 | 6·8 | 0·9 | 39·0 | 26·4 | 27·9 | 0·613 | 43·0 | 67·2 | 56·8 | 1·8 | 0·022 |
| 2015 | Roraima | 0·6 | 8·0 | 0·7 | 31·3 | 11·4 | 12·6 | 0·636 | 8·9 | 45·1 | 27·1 | 3·6 | 0·080 |
| 2015 | Santa Catarina | 0·2 | 9·3 | 0·9 | 13·8 | 8·8 | 9·9 | 0·695 | 15·4 | 80·0 | 104·0 | 6·0 | 0·047 |
| 2015 | São Paulo | 0·3 | 6·3 | 0·6 | 16·2 | 9·8 | 10·7 | 0·713 | 14·8 | 72·7 | 99·9 | 13·4 | 0·035 |
| 2015 | Sergipe | 0·3 | 7·1 | 0·7 | 53·7 | 41·8 | 42·8 | 0·607 | 34·0 | 10·9 | 20·4 | 3·4 | 0·027 |
| 2015 | Tocantins | 0·6 | 7·3 | 1·0 | 29·8 | 15·1 | 16·8 | 0·601 | 21·1 | 21·1 | 38·9 | 1·9 | 0·043 |
| 2016 | Acre | 0·6 | 6·4 | 0·9 | 33·4 | 17·8 | 19·4 | 0·597 | - | - | - | 2·0 | - |
| 2016 | Alagoas | 0·2 | 3·8 | 0·3 | 57·6 | 47·2 | 47·6 | 0·552 | 21·9 | 10·7 | 36·5 | 6·2 | 0·036 |
| 2016 | Amapá | 0·4 | 6·3 | 0·4 | 42·3 | 20·8 | 21·6 | 0·655 | 21·5 | 25·6 | 44·7 | 2·1 | 0·042 |
| 2016 | Amazonas | 0·3 | 6·5 | 0·5 | 40·1 | 25·0 | 25·8 | 0·625 | 6·5 | 17·0 | 57·8 | 0·8 | 0·137 |
| 2016 | Bahia | 1·4 | 5·5 | 0·7 | 37·4 | 28·9 | 31·0 | 0·587 | - | 34·5 | - | 0·9 | - |
| 2016 | Ceará | 0·3 | 7·4 | 0·6 | 41·1 | 32·0 | 32·9 | 0·596 | 33·4 | 26·6 | 44·7 | 1·2 | 0·029 |
| 2016 | Distrito Federal | 0·1 | 4·4 | 0·6 | 27·3 | 19·8 | 20·5 | 0·789 | 47·2 | 202·8 | 77·3 | 8·7 | 0·018 |
| 2016 | Espírito Santo | 0·2 | 4·7 | 0·5 | 39·1 | 30·0 | 30·6 | 0·673 | 45·4 | 68·3 | 128·9 | 3·8 | 0·020 |
| 2016 | Goiás | 0·4 | 7·6 | 0·9 | 44·5 | 31·6 | 32·9 | 0·647 | 54·9 | 73·6 | 72·2 | 1·2 | 0·018 |
| 2016 | Maranhão | 0·6 | 3·8 | 0·4 | 24·5 | 15·6 | 16·7 | 0·502 | 23·9 | 16·7 | 26·6 | 3·5 | 0·037 |
| 2016 | Mato Grosso | 1·0 | 6·1 | 0·8 | 36·4 | 22·4 | 24·2 | 0·659 | 61·8 | 120·3 | 92·1 | 3·3 | 0·015 |
| 2016 | Mato Grosso do Sul | 0·4 | 8·6 | 1·3 | 26·3 | 14·8 | 16·5 | 0·647 | 58·6 | 98·5 | 116·6 | 8·3 | 0·015 |
| 2016 | Minas Gerais | 0·3 | 8·1 | 0·8 | 25·4 | 18·3 | 19·5 | 0·658 | 25·3 | 86·6 | 121·7 | 6·7 | 0·031 |
| 2016 | Pará | 0·7 | 3·8 | 0·4 | 41·0 | 28·0 | 29·1 | 0·575 | 28·3 | 26·0 | 49·4 | 1·2 | 0·034 |
| 2016 | Paraíba | 0·2 | 5·4 | 0·4 | 37·9 | 29·8 | 30·4 | 0·571 | 22·1 | 9·1 | 13·1 | 1·9 | 0·042 |
| 2016 | Paraná | 0·3 | 7·6 | 0·9 | 29·7 | 20·7 | 21·8 | 0·679 | 47·7 | 109·5 | 76·3 | 4·6 | 0·019 |
| 2016 | Pernambuco | 0·2 | 5·4 | 0·5 | 47·8 | 36·2 | 36·9 | 0·590 | 22·6 | 49·2 | 41·0 | 9·1 | 0·032 |
| 2016 | Piauí | 0·4 | 8·3 | 0·8 | 20·4 | 12·2 | 13·5 | 0·548 | 21·6 | 15·4 | 28·1 | 0·7 | 0·045 |
| 2016 | Rio de Janeiro | 0·5 | 4·7 | 0·8 | 35·1 | 29·9 | 31·2 | 0·706 | 24·1 | 58·6 | 78·7 | 8·5 | 0·031 |
| 2016 | Rio Grande do Norte | 0·8 | 7·4 | 0·9 | 48·4 | 39·2 | 40·9 | 0·602 | 6·6 | 6·5 | 9·2 | 3·0 | 0·104 |
| 2016 | Rio Grande do Sul | 0·3 | 12·1 | 2·1 | 27·8 | 21·5 | 24·0 | 0·690 | 48·9 | 93·3 | 78·4 | 8·6 | 0·017 |
| 2016 | Rondônia | 0·7 | 6·9 | 0·9 | 40·4 | 27·3 | 29·0 | 0·618 | 44·9 | 61·4 | 57·0 | 1·3 | 0·022 |
| 2016 | Roraima | 0·6 | 8·1 | 0·7 | 30·9 | 11·3 | 12·6 | 0·642 | 10·3 | 61·6 | 42·2 | 1·3 | 0·086 |
| 2016 | Santa Catarina | 0·2 | 9·6 | 0·9 | 14·1 | 9·0 | 10·1 | 0·699 | 15·0 | 65·8 | 97·8 | 3·5 | 0·054 |
| 2016 | São Paulo | 0·3 | 6·2 | 0·6 | 15·8 | 9·4 | 10·4 | 0·717 | 14·4 | 73·5 | 101·8 | 13·9 | 0·035 |
| 2016 | Sergipe | 0·4 | 7·2 | 0·7 | 56·4 | 44·3 | 45·3 | 0·612 | 30·4 | 10·0 | 32·1 | 1·4 | 0·031 |
| 2016 | Tocantins | 0·6 | 7·4 | 1·0 | 30·8 | 15·9 | 17·5 | 0·607 | 18·1 | 16·2 | 38·1 | 0·4 | 0·054 |
| 2017 | Acre | 0·6 | 6·5 | 0·9 | 32·9 | 17·3 | 18·8 | 0·602 | - | - | - | - | - |
| 2017 | Alagoas | 0·2 | 3·8 | 0·3 | 56·3 | 45·9 | 46·4 | 0·556 | - | - | - | - | - |
| 2017 | Amapá | 0·3 | 6·5 | 0·4 | 41·5 | 20·0 | 20·8 | 0·659 | - | - | - | - | - |
| 2017 | Amazonas | 0·3 | 6·4 | 0·5 | 38·3 | 23·8 | 24·6 | 0·629 | - | - | - | - | - |
| 2017 | Bahia | 1·3 | 5·6 | 0·7 | 37·1 | 28·6 | 30·6 | 0·591 | - | - | - | - | - |
| 2017 | Ceará | 0·3 | 7·2 | 0·5 | 37·7 | 29·0 | 29·9 | 0·600 | - | - | - | - | - |
| 2017 | Distrito Federal | 0·1 | 4·4 | 0·6 | 26·3 | 19·0 | 19·6 | 0·792 | - | - | - | - | - |
| 2017 | Espírito Santo | 0·2 | 4·6 | 0·4 | 37·8 | 28·8 | 29·5 | 0·677 | - | - | - | - | - |
| 2017 | Goiás | 0·3 | 7·6 | 0·9 | 42·3 | 29·9 | 31·1 | 0·650 | - | - | - | - | - |
| 2017 | Maranhão | 0·6 | 3·8 | 0·4 | 23·9 | 15·1 | 16·2 | 0·507 | - | - | - | - | - |
| 2017 | Mato Grosso | 1·0 | 6·1 | 0·8 | 35·4 | 21·6 | 23·4 | 0·662 | - | - | - | - | - |
| 2017 | Mato Grosso do Sul | 0·4 | 8·8 | 1·3 | 26·6 | 14·9 | 16·7 | 0·650 | - | - | - | - | - |
| 2017 | Minas Gerais | 0·3 | 8·2 | 0·8 | 24·8 | 17·8 | 18·9 | 0·661 | - | - | - | - | - |
| 2017 | Pará | 0·7 | 3·9 | 0·4 | 40·0 | 27·2 | 28·4 | 0·579 | - | - | - | - | - |
| 2017 | Paraíba | 0·2 | 5·4 | 0·4 | 36·3 | 28·4 | 29·1 | 0·574 | - | - | - | - | - |
| 2017 | Paraná | 0·2 | 7·7 | 0·9 | 29·5 | 20·4 | 21·6 | 0·682 | - | - | - | - | - |
| 2017 | Pernambuco | 0·2 | 5·7 | 0·5 | 48·8 | 36·9 | 37·6 | 0·594 | - | - | - | - | - |
| 2017 | Piauí | 0·4 | 8·2 | 0·8 | 19·9 | 11·8 | 13·0 | 0·552 | - | - | - | - | - |
| 2017 | Rio de Janeiro | 0·5 | 4·9 | 0·8 | 36·8 | 31·3 | 32·6 | 0·709 | - | - | - | - | - |
| 2017 | Rio Grande do Norte | 0·9 | 7·8 | 0·9 | 47·9 | 38·5 | 40·3 | 0·605 | - | - | - | - | - |
| 2017 | Rio Grande do Sul | 0·3 | 12·6 | 2·2 | 27·6 | 21·2 | 23·7 | 0·693 | - | - | - | - | - |
| 2017 | Rondônia | 0·7 | 6·9 | 0·9 | 39·5 | 26·7 | 28·3 | 0·622 | - | - | - | - | - |
| 2017 | Roraima | 0·6 | 8·0 | 0·6 | 30·3 | 11·1 | 12·3 | 0·646 | - | - | - | - | - |
| 2017 | Santa Catarina | 0·2 | 9·7 | 0·9 | 13·7 | 8·6 | 9·8 | 0·702 | - | - | - | - | - |
| 2017 | São Paulo | 0·3 | 6·2 | 0·6 | 15·9 | 9·5 | 10·4 | 0·720 | - | - | - | - | - |
| 2017 | Sergipe | 0·3 | 7·3 | 0·7 | 54·3 | 42·2 | 43·2 | 0·616 | - | - | - | - | - |
| 2017 | Tocantins | 0·6 | 7·5 | 1·0 | 29·7 | 15·2 | 16·8 | 0·611 | - | - | - | - | - |
| * [=1/(voluntary return of firearms+illegal carrying of firearms)] | | | |  |  |  |  |  |  |  |  |  |  |
